# Asymmetric Catalytic Ketimine Mannich Reactions and Related Transformations

**DOI:** 10.3390/catal11060712

**Published:** 2021-06-07

**Authors:** Changgong Xu, Carlyn Reep, Jamielyn Jarvis, Brandon Naumann, Burjor Captain, Norito Takenaka

**Affiliations:** 1 Chemistry Program, Department of Biomedical and Chemical Engineering and Sciences, Florida Institute of Technology, 150 West University Boulevard, Melbourne, FL 32901-6975, USA;; 2 Department of Chemistry, University of Miami, 1301 Memorial Drive, Coral Gables, FL 33146-0431, USA;

**Keywords:** ketimine Mannich, ketimine allylation, aza-Morita–Baylis–Hillman, asymmetric catalysis, α-tertiary amine, β-amino carbonyl

## Abstract

The catalytic enantioselective ketimine Mannich and its related reactions provide direct access to chiral building blocks bearing an α-tertiary amine stereogenic center, a ubiquitous structural motif in nature. Although ketimines are often viewed as challenging electrophiles, various approaches/strategies to circumvent or overcome the adverse properties of ketimines have been developed for these transformations. This review showcases the selected examples that highlight the benefits and utilities of various ketimines and remaining challenges associated with them in the context of Mannich, allylation, and aza-Morita-Baylis-Hillman reactions as well as their variants.

## 1. Introduction

The condensation reaction between in situ generated imines and enols to form β-amino carbonyl compounds was reported by Carl U. F. Mannich for the first time in 1912 [1], This is the transformation that is widely known as the Mannich reaction today. Since it provides direct access to synthetically useful chiral building blocks from readily available carbonyls and amines, it attracted huge attention from the synthetic community and is now recognized as one of the most important chemical transformations (selected reviews; [2–12]). In order to circumvent the inherent difficulties associated with the classical Mannich reaction such as regio-, stereo-, and product-selectivities, preformed imines and/or enolates have often been employed. These nonclassical Mannich variants are broadly regarded as the Mannich reaction while they are alternatively called the Mannich-type reaction, aza- or imino-aldol reaction to be more specific. However, distinction between these terminologies largely remains dependent on an individual author; thus, the term “Mannich reaction” is used for all Mannich-type transformations covered in this review for clarity unless otherwise stated.

**Figure 1.**
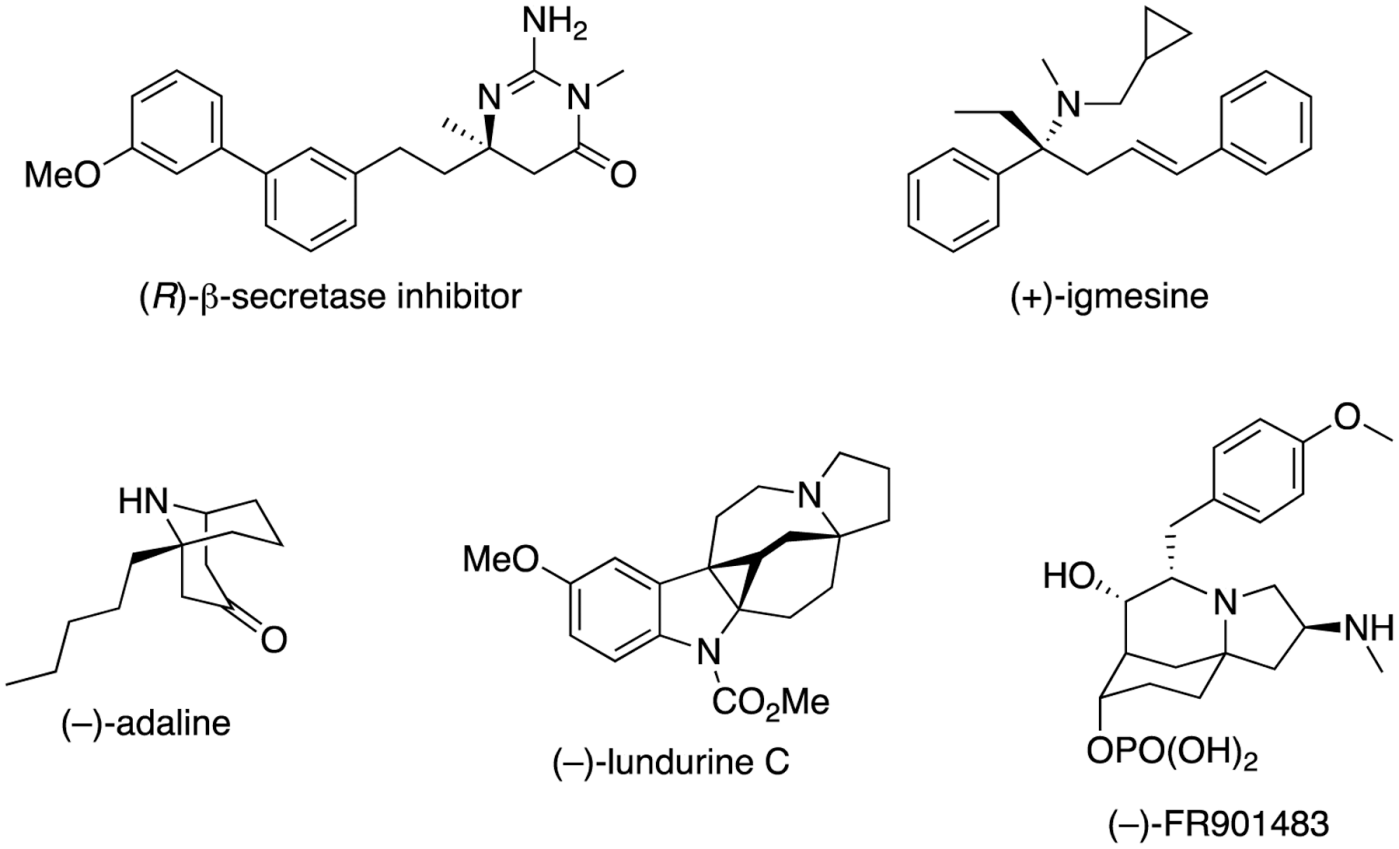
Examples of bioactive compounds and natural products bearing an α-tertiary amine stereogenic center.

**Figure 2.**
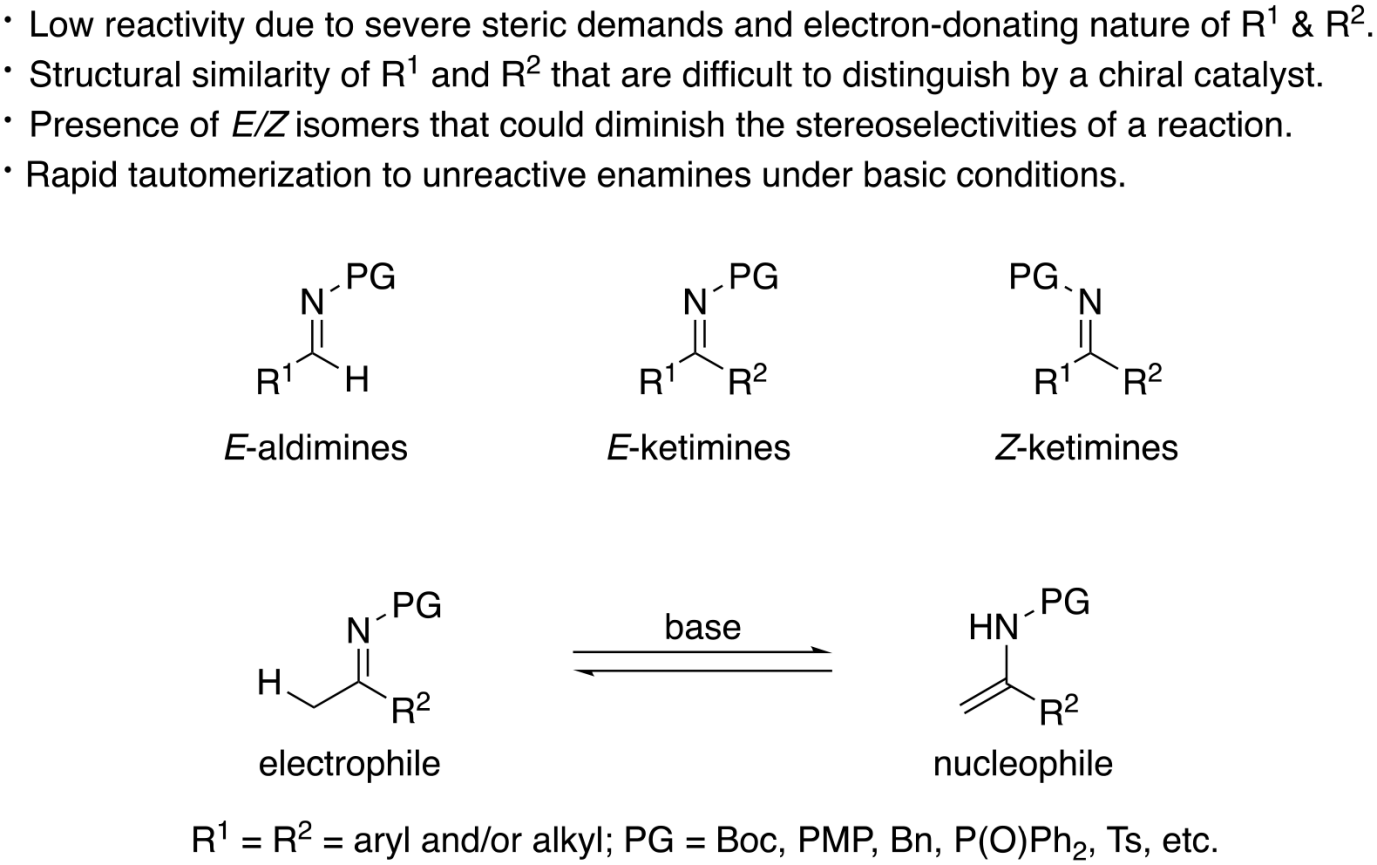
Notorious properties of unmodified ketimines as electrophiles.

**Scheme 1.**
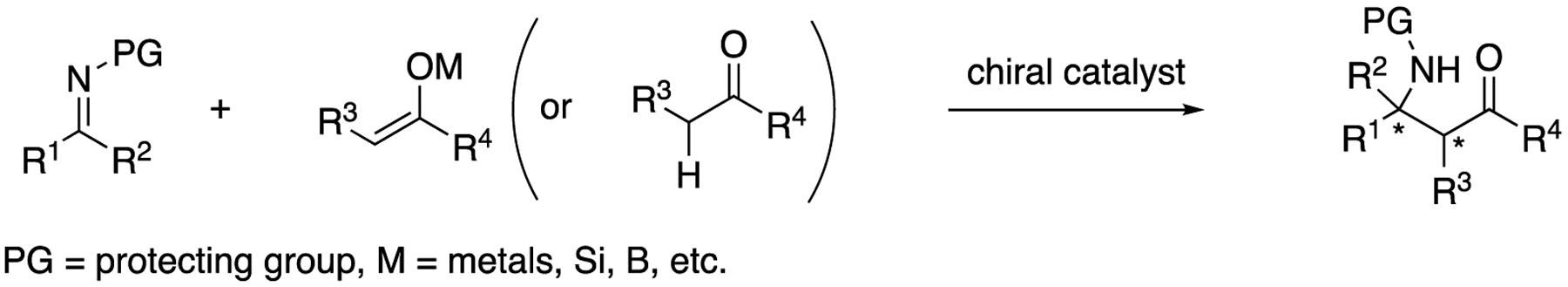
Asymmetric catalytic ketimine Mannich reaction.

Over the years, the asymmetric catalytic Mannich reaction of ketimines (Scheme 1) has been of significant interest to synthetic and medicinal chemists as a way to access synthetically versatile compounds bearing an α-tertiary amine stereogenic center (selected reviews; [13–23]). It is because optically pure chiral α-tertiary amines are a key structural motif found in a large number of biologically relevant molecules and natural products (Figure 1) [24–28]. However, ketimines are much more challenging electrophiles than aldimines for the Mannich and its related reactions (e.g., allylation) due to some notorious structural properties of the formers (Figure 2). In general, diaryl, aryl alkyl, and dialkyl ketimines (i.e., unmodified ketimines) are poor electrophiles because of the severe steric demands and the electron-donating nature of the two groups flanking a C=N bond. Furthermore, these two substituents on a prochiral center are structurally similar thus difficult to distinguish by a chiral catalyst, often leading to low stereoselectivities.

**Scheme 2.**
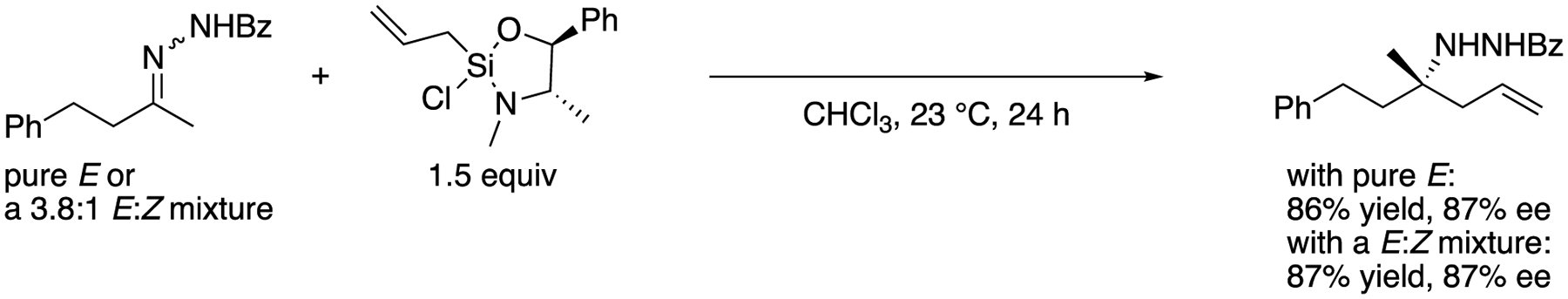
Selected example of in situ *E/Z* isomerization of ketimine.

Nonendocyclic ketimines can equilibrate between the *E* and Z forms in solution at room temperature by an inversion, or rotation, and furthermore, through ketimine-enamine tautomerization, if they have an α-hydrogen atom [29]. Therefore, ketimines are often available only as a mixture of *E* and *Z* isomers that could lead to diminished stereoselectivities in the asymmetric transformations. An interesting observation regarding the *E/Z* isomerism was reported by Leighton and coworkers in 2004 (Scheme 2) [30]. Their chiral allylation reagent provided the homoallylic amine in the same yield and ee regardless of the *E/Z* isomeric ratio of the ketimine used. It was presumed that the ketimine isomerized under the reaction conditions, and one isomer selectively underwent the allylation reaction. Despite this observation, it remains largely elusive how to logically develop such catalytic enantioselective methods. Alternatively catalytic methods that tolerate *N*-unsubstituted ketimine salts, thus obviating the *E/Z* isomerism, have been developed in recent years [31]. In 2009, Gosselin, Zhang, and coworkers reported the first examples of catalytic hydrogenation of *N*-unsubstituted ketimine salts (not shown) [32], These reported examples bode well for the further development of ketimine Mannich and related transformations.

The majority of the work published on the ketimine Mannich reaction and its related transformations employed ketimines where some notorious properties of unmodified ketimines summarized in Figure 2 were absent. In this mini-review, unmodified ketimines refer to nonendocyclic ketimines in which no electron-withdrawing groups and/or alkynes are attached to the carbon atom of a C=N bond (sp hybridized carbon atoms are electronegative; for example, the p*K*_*a*_ value of acetylene is 24 in H_2_O). Therefore, this review is organized based on the structures of ketimines under each reaction class. It is arguable whether ketimines bearing strongly electron-withdrawing *N*-protecting groups such as *N*-tosyl ketimines should be categorized as unmodified ketimines or not; however, they are also included as part of unmodified ketimines as long as those ketimines are tautomerizable. Where appropriate, each ketimine class is further classified based on the kind of nucleophiles and/or the methods to generate them. The objective of this review is to highlight the benefits and utilities of different ketimines and remaining challenges associated with them in the context of Mannich, allylation, and aza-Morita-Baylis-Hillman reactions as well as their variants. We hope the information provided herein promotes the study and use of ketimines that are in general considered problematic substrates. Only a summary of the results is shown in each case to focus on the objective, unless otherwise noted. Interested readers may want to refer to the corresponding literature for the detailed catalytic mechanisms and the stereochemical models that are usually provided therein.

In this review, selected examples published over the past decade or so and some pioneering works are covered. With respect to the transformations related to the ketimine Mannich reaction, only methods that employ enolates or its equivalents (e.g., silyl enol ethers and allyl metals) as nucleophiles to provide β-amino carbonyl compounds or its equivalents are covered, while vinylogous Mannich reactions are included. The asymmetric catalytic methods of ketimines that provide α-amino carbonyl compounds such as Strecker reaction (selected review; [33]), nitro-Mannich reaction (selected review; [34]), aza-benzoin reaction (selected references [35,36]) are not included. The asymmetric umpolung addition of ketimines to electrophiles (selected references; [37–44]) is beyond the scope of this review. Oxidative asymmetric Mannich reaction is an approach to avoid some drawbacks of preformed ketimines by in situ oxidation of corresponding amines, which is recently reviewed [45]. Interested readers may wish to refer to these selected articles.

## 2. Activated and/or Cyclic Ketimines in the Mannich Reaction

### 2.1. Endocyclic Ketimines with Electron-Withdrawing Substituents

#### 2.1.1. The First Examples of Catalytic Enantioselective Ketimine Mannich Reactions

**Scheme 3.**
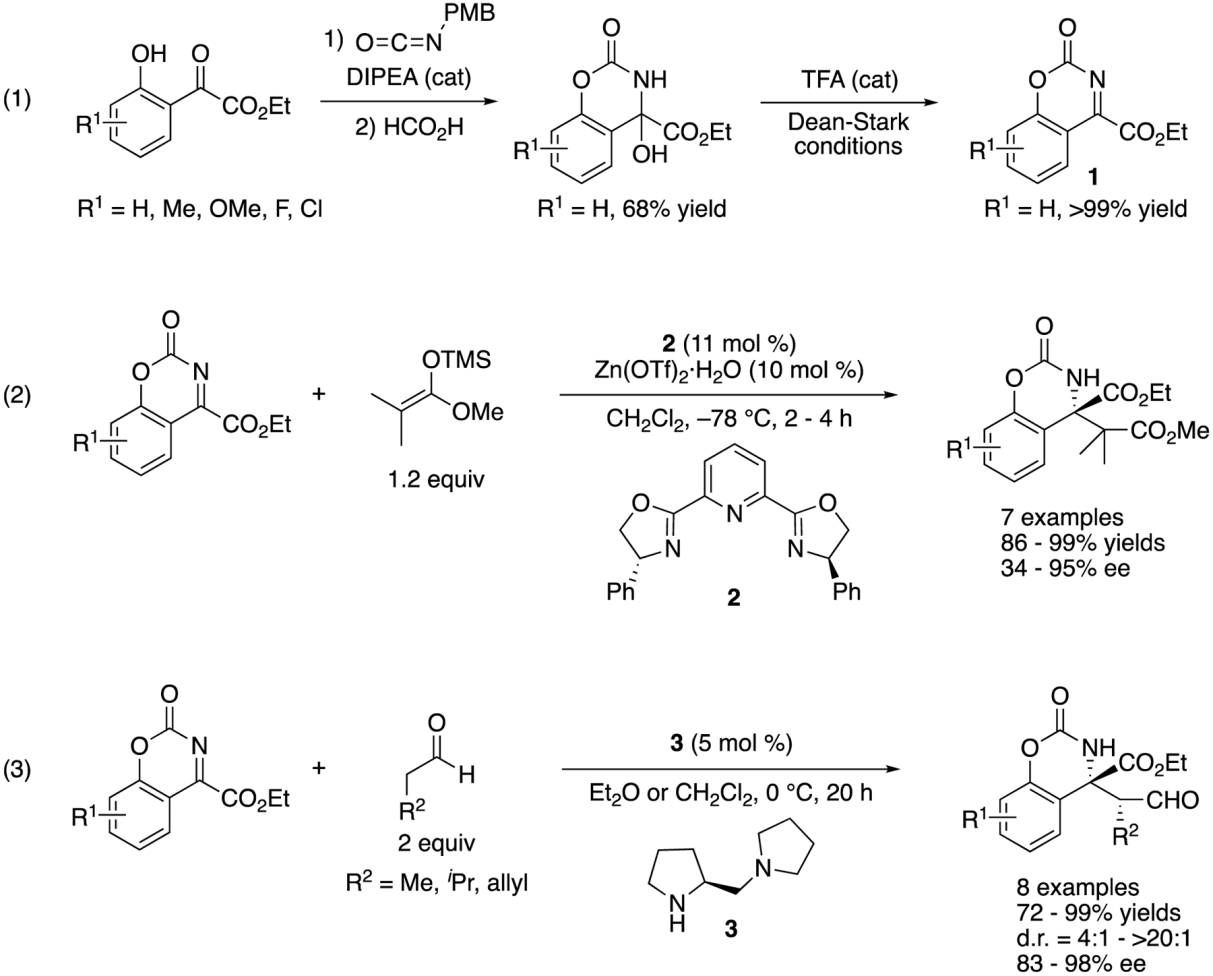
The first asymmetric catalytic ketimine Mannich reactions.

In 2003, Jørgensen and coworkers described their approach toward the first catalytic enantioselective ketimine Mannich reaction (Scheme 3) [46], They designed and developed the endocyclic aryl ketiminoesters (**1**) that circumvented the poor electrophilicity the structural similarity of two groups flanking a C=N bond, the tautomerization, and the *E/Z* isomerism associated with unmodified ketimines. They evaluated their ketiminoesters for the Mukaiyama-Mannich reaction (2) and obtained the products in 86–99% yields with 34–95% ee. In a subsequent year, they reported the application of the same ketiminoesters for direct organocatalytic enantioselective Mannich reaction that provided the corresponding products in 82–99% yields with 4:1–>20:1 diastereoselectivities and 72–98% ee (3) [47]. It is worthy of note that these ketimines reacted well with catalytically generated enamines in the presence of small aldehydes such as propionaldehyde (i.e., good electrophiles). The two consecutive reports clearly demonstrated that these endocyclic tautomerization-free ketiminoesters were viable substrates for the enantioselective catalytic Mannich reactions via either the electrophile activation (i.e., Lewis acid catalysis) or the nucleophile activation (i.e., Lewis base catalysis) methods to access chiral synthetic building blocks bearing an α-tertiary amine stereogenic center, inspiring the later developments.

#### 2.1.2. Direct Mannich

**Scheme 4.**
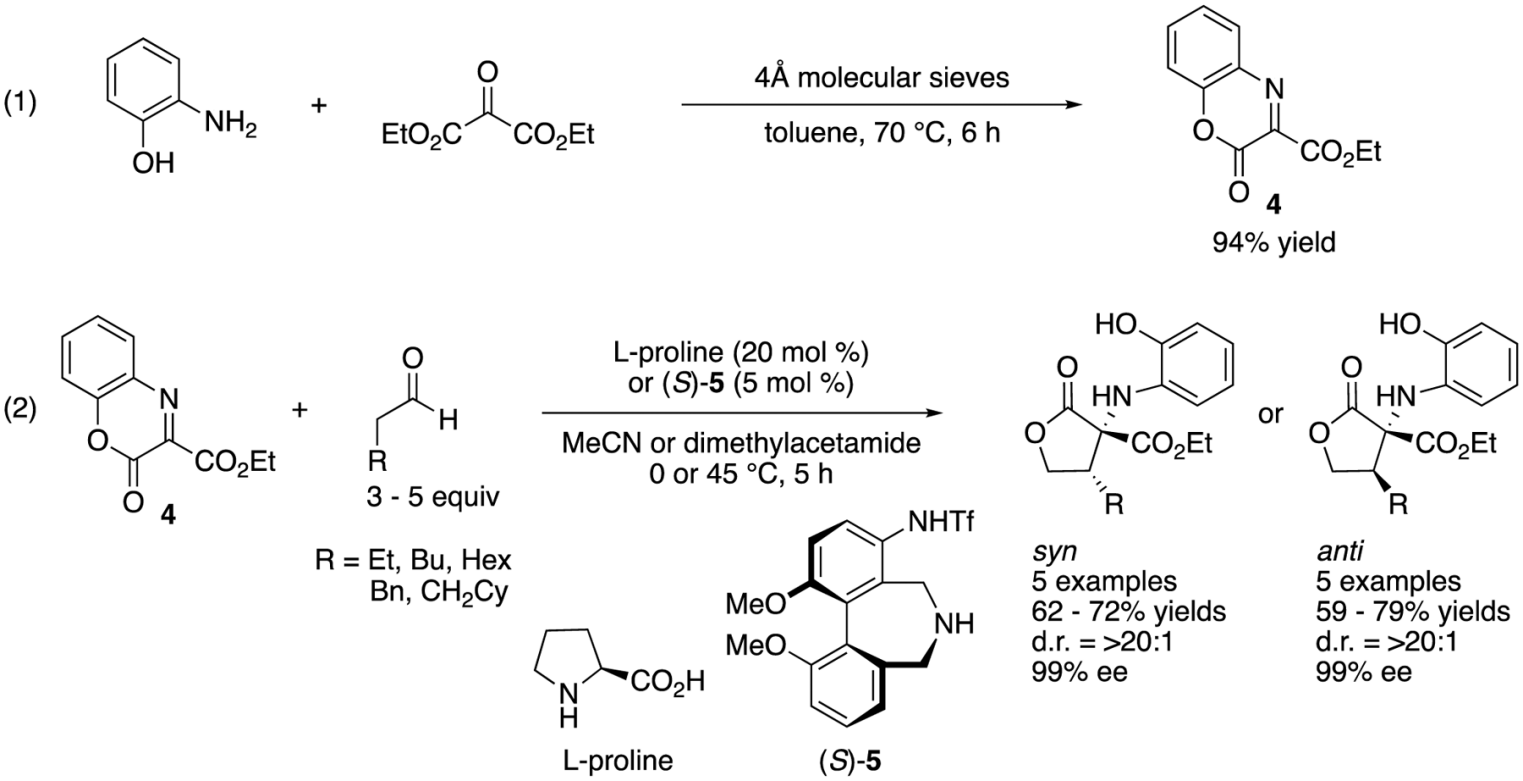
Stereodivergent direct catalytic asymmetric Mannich reaction of an endocyclic ketiminoester.

In 2012, Kano, Maruoka, and coworkers developed synthetically flexible ketiminoester **4** and demonstrated its utility in direct organocatalytic enantioselective ketimine Mannich reactions (Scheme 4) [48]. More specifically, they designed an easy-to-prepare nonaromatic ketiminoester that provides relatively versatile synthetic building blocks because an ester group is synthetically more flexible than an aryl group in general. As **4** is relatively reactive, it is a convenient electrophile to build a chiral α-tertiary amine motif into compounds. It is notable that their method is stereodivergent and afforded either syn- or anti-γ-lactones after subsequent NaBH_4_ reduction of the aldehyde products in 59–79% yields with excellent diastereo- and enantio-selectivities (>20:1 and 99% ee, respectively). l-Proline and axial chiral anime **5** afforded syn- and anti-γ-lactones, respectively.

**Scheme 5.**
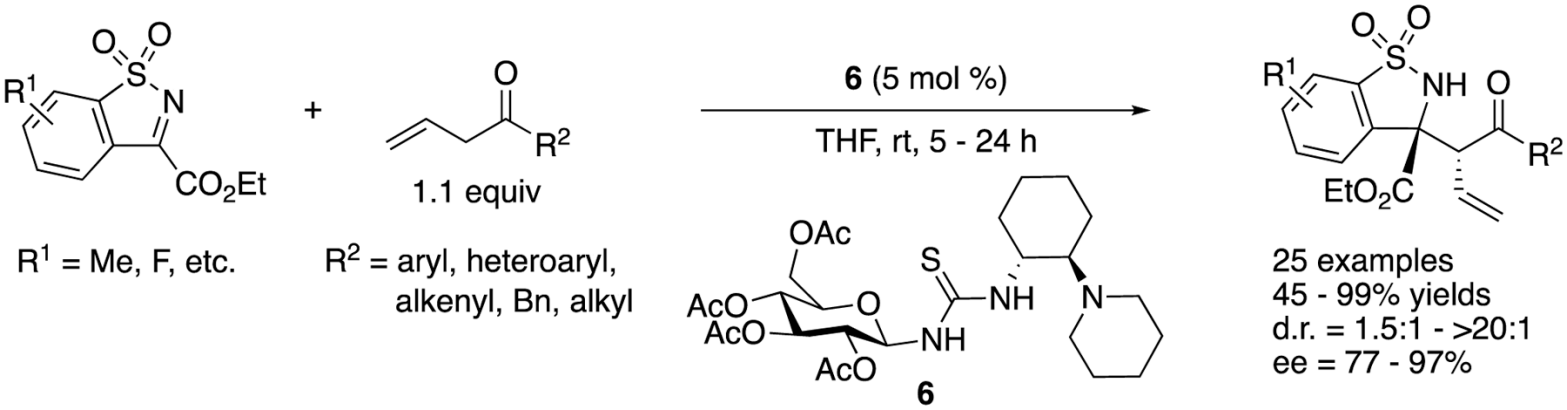
Regioselective asymmetric Mannich reaction of allylic ketones with *N*-sulfonyl ketiminoester.

Endocyclic *N*-sulfonyl ketiminoesters have often been employed in catalytic asymmetric Mannich reactions because resulting chiral benzosultams are an important structural motif present in many medicinally important molecules, and this class of ketimines is relatively easy substrates to work with (highly electrophilic, no tautomerization, and no *E/Z* isomer issues). As such, we discuss selected examples in Scheme 5 and 10 (for other selected examples; see [49–53]).

In 2015, Ma and coworkers developed a highly regio-, diastereo- and enantio-selective Mannich reaction of (β, γ-unsaturated ketones with *N*-sulfonyl ketiminoesters by employing a saccharide-derived tertiary amino-thiourea catalyst (Scheme 5) [54]. The reaction scope was found broad in allylic ketones and included aryl, heteroaryl, alkenyl, benzyl, and alkyl allyl ketones, affording the corresponding products in 45–99% yields with 1.5:1–>20:1 diastereoselectivities and 77–97% ee. The β, γ-unsaturated-ketone products did not isomerize to the corresponding α,β-unsaturated ketones, which highlighted the mildness of the reaction conditions. It is noteworthy that benzyl allyl ketone provided a desired regioisomer in 64% yield.

Chiral dihydroquinazolinones are one of the important motifs found in biologically relevant molecules. Since the catalytic asymmetric Mannich reaction of dihydroquinazolines provides direct access to enantio-enriched dihydroquinazolinones and dihydroquinazolines are relatively reactive ketimines, many contributions have been reported, which highlight the usefulness of the asymmetric Mannich reaction of dihydroquinazolines for medicinal chemistry. We discuss two selected examples herein (for other selected examples, see [55–57]).

**Scheme 6.**
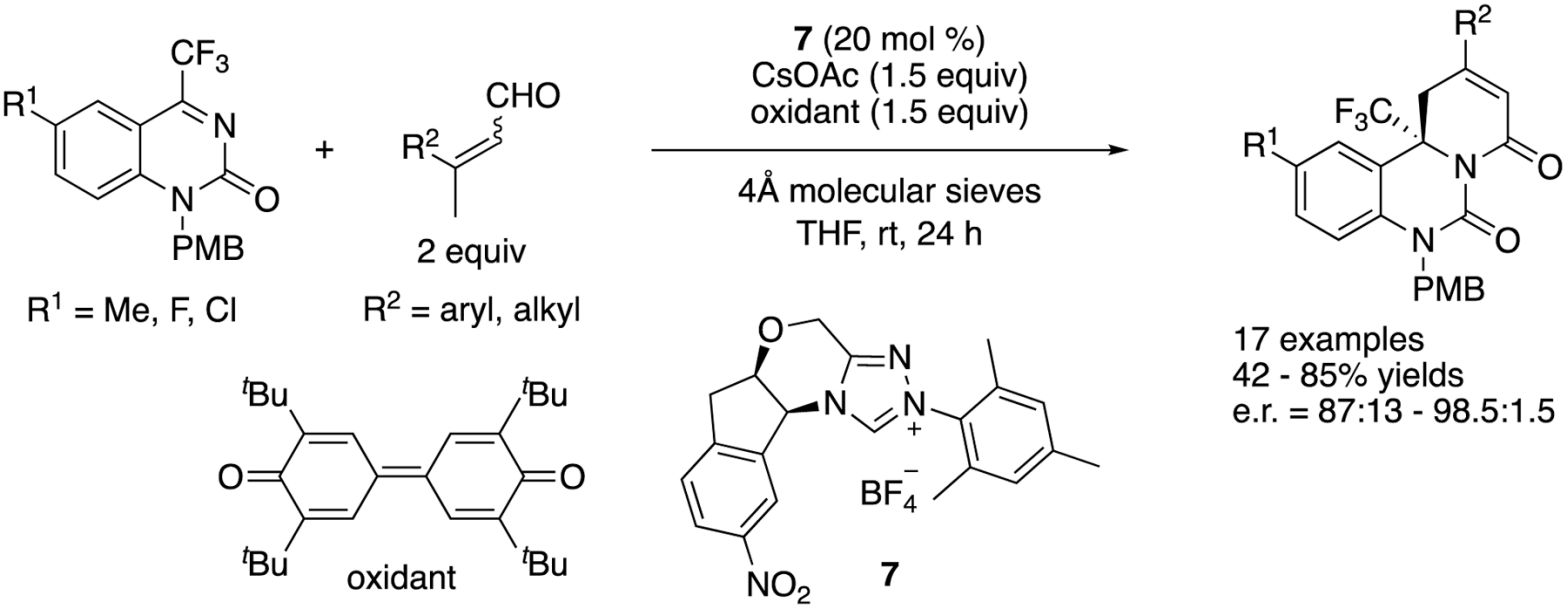
The oxidative NHC-catalyze [4 + 2] annulation reaction of enals and dihydroquinazolines.

In 2017, Enders and coworkers developed an enantioselective oxidative NHC-catalyzed [4 + 2] annulation reaction of β-methyl enals and trifluoromethyl dihydroquinazolines (Scheme 6) [58]. The catalytically generated dienolate proved highly effective for addition to trifluoromethyl dihydroquinazolines, providing heterocyclic dihydroquinazolinone derivatives bearing a trifluoromethyl group and a tetrasubstituted stereogenic center in 42–85% yields with 87:13–98.5:1.5 enantiomeric ratios.

**Scheme 7.**
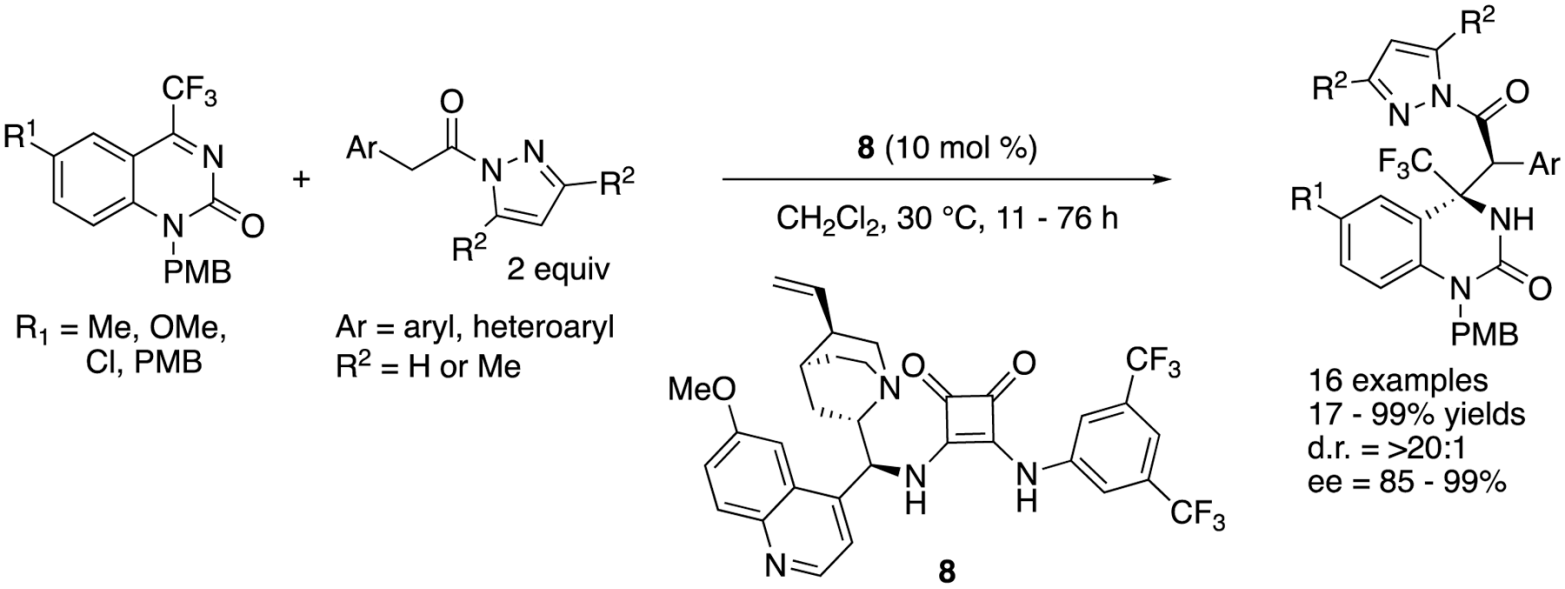
Asymmetric Mannich reaction of pyrazoleanides and dihydroquinazolines.

In 2018, Xu, Yuan, and coworkers developed a diastereo- and enantio-selective catalytic Mannich reaction of pyrazoleamides with trifluoromethyl dihydroquinazolines by employing a bifunctional cinchona-derived squaramide catalyst (Scheme 7) [59]. The method provided a wide range of trifluoromethyl dihydroquinazolinone derivatives bearing adjacent tertiary and quaternary stereogenic centers in 17–99% yields with >20:1 diastereoselectivity and 85–99% ee. It is noteworthy that they successfully demonstrated a multi-mmol scale reaction that provided 1.21 g of the desired product with no loss in yields and stereoselectivities (R^1^ = Cl, Ar = Ph, R^2^ = H, 91% yield, >20:1 d.r., 99% ee).

Indole derivatives are privileged structures and thus found in numerous pharmaceutical compounds. Moreover, 2-Aryl-3*H*-indol-3-ones are valuable synthons for the synthesis of complex indole-derived molecules. This class of ketimines was demonstrated as viable electrophiles in the proline-catalyzed Mannich reactions with aldehydes and ketones by the groups of Xie and Rueping in 2011 and 2012, respectively [60,61] (not shown), highlighting their sufficient electrophilicity.

**Scheme 8.**
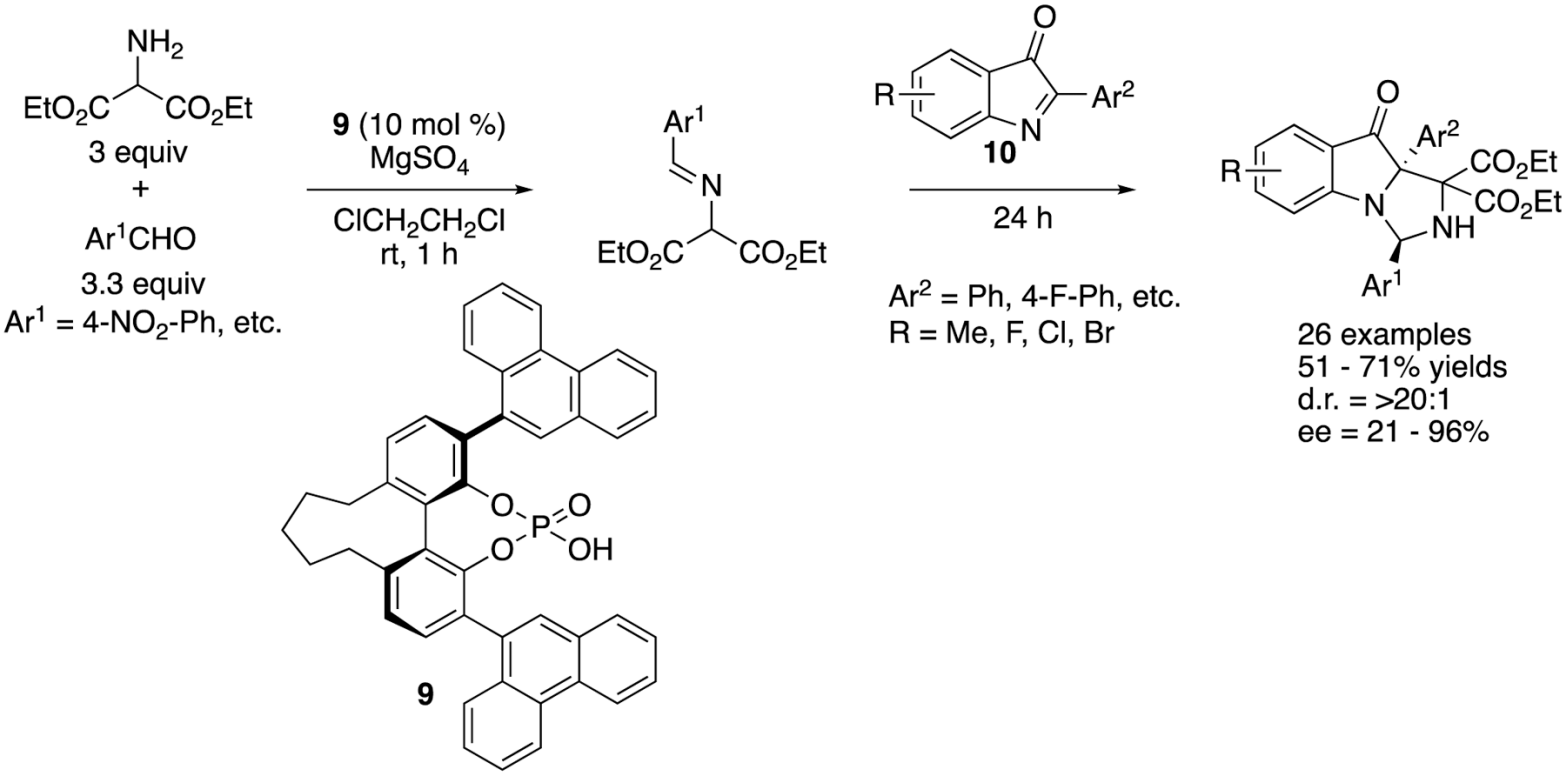
Chiral phosphoric acid-catalyzed Mannich reaction of 2-aryl-3*H*-indol-3-ones.

In 2019, Fu and coworkers developed a chiral phosphoric acid-catalyzed Mannich reaction of 2-aryl-3*H*-indol-3-ones (**10**) with Schiff bases generated in situ from aromatic aldehydes and diethyl 2-aminomalonate (Scheme 8) [62]. This method afforded complex heterocycles bearing a nitrogen-substituted quaternary and aminal stereogenic centers that are not trivial to make in 51–71% yields with >20:1 diastereoselectivities and 21–96% ee.

**Scheme 9.**
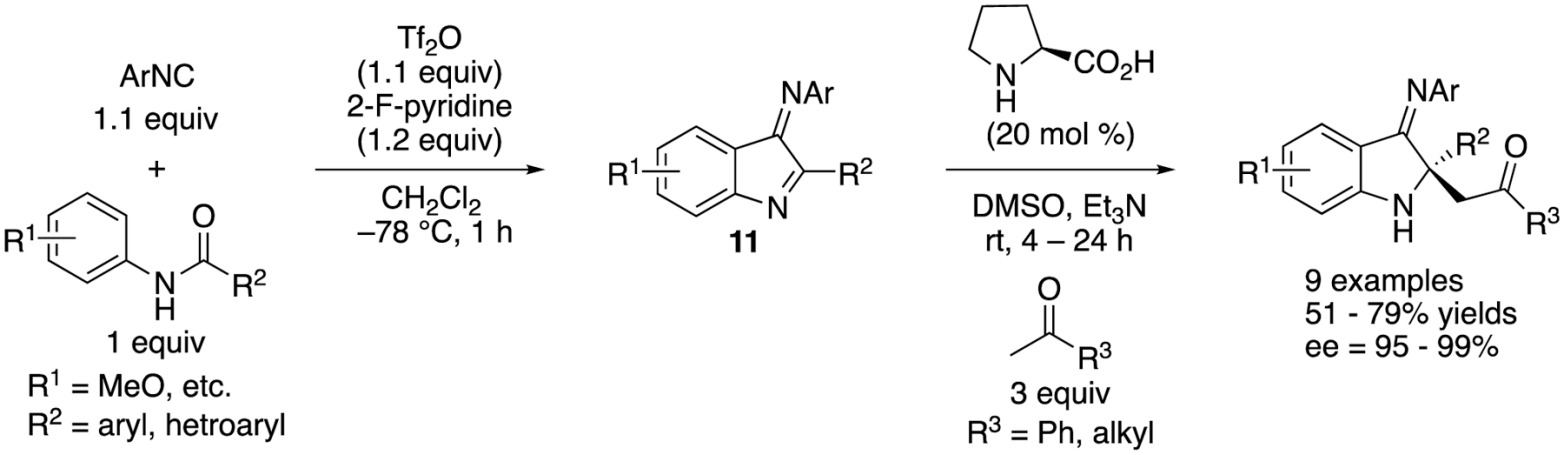
Proline-catalyzed Mannich reaction of 3-iminoindoles generated in situ from amides and isocyanides.

In 2019, Zheng, Ye, Huang, and coworkers reported a one-pot construction of chiral 2,2-disubstituted 3-iminoindolines (Scheme 9) [63]. In addition, 3-Iminoindoles (**11**) were generated in situ from amides and isocyanides in CH_2_CI_2_ for 1 h, and then the resulting reaction mixtures were treated with a DMSO solution of ketones, proline and Et_3_N to provide the Mannich products in 51–79% yields with 95–99% ee. This method constructed 3 carbon-carbon bonds, 1 ring, and 1 nitrogen-substituted quaternary stereogenic center in one pot.

#### 2.1.3. Preformed Enolate Equivalents

**Scheme 10.**
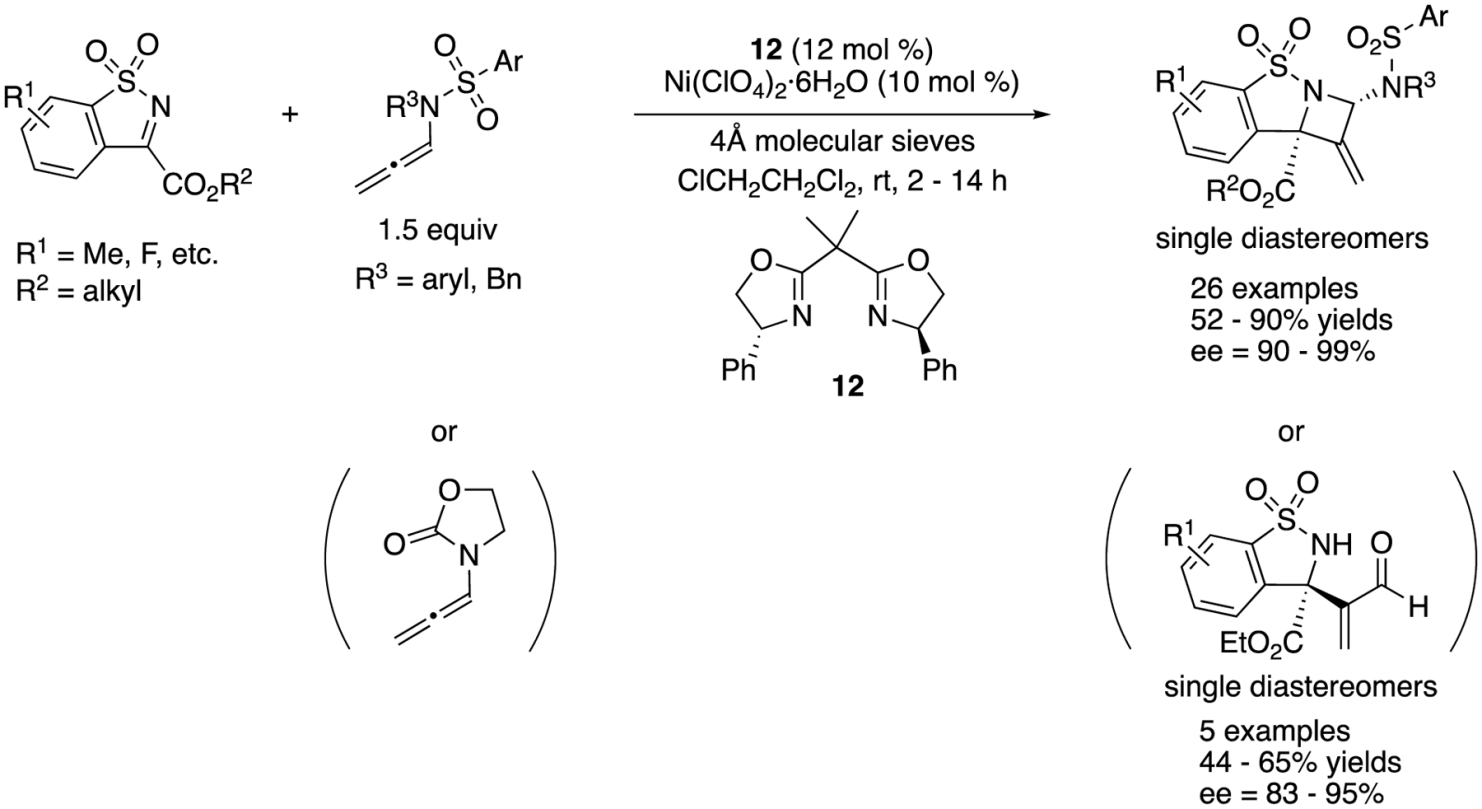
Enantioselective [2 + 2] cycloaddition of *N*-allenamides with *N*-sulfonyl ketiminoester.

In 2017, Jia and coworkers reported an enantioselective [2 + 2] cycloaddition of *N*-sulfonyl ketiminoesters with *N*-allenamides that produced chiral azetidines (Scheme 10) [64], They screened several Lewis acidic metal salts and chiral BOX ligands and then identified Ni-**12** complex as an optimal catalyst for this transformation. *N*-Allenyl oxazolidinones were also found to be viable nucleophiles for their method. The reactions with *N*-allenyl oxazolidinones provided the corresponding acrylaldehydes after standard aqueous work-up, although it took 3 h at 60 °C to hydrolyze azetidines derived from *N*-allenamides with TsOH·H_2_O. It is noteworthy that only one diastereomer was detected in all cases. The azetidines and acrylaldehydes were obtained in 52–90% yields with 90–99% ee and in 44–65% yields with 83–95% ee, respectively.

### 2.2. Endocydic Ketimines without Electron-Withdrawing Substituents

All of the notorious properties of unmodified ketimines summarized in Figure 2 are absent in the ketimines discussed above. However, these contributions clearly demonstrated that creative applications of relatively easy-to-use ketimines for Mannich reaction can provide synthetically versatile and medicinally important β-amino carbonyl compounds and their variants that are otherwise difficult to synthesize. In this section, endocyclic ketimines that are not substituted with electron-withdrawing substituents at their C=N carbon atoms are discussed. These ketimines are less electrophilic and thus, arguably, more challenging substrates.

#### 2.2.1. Direct Mannich

**Scheme 11.**
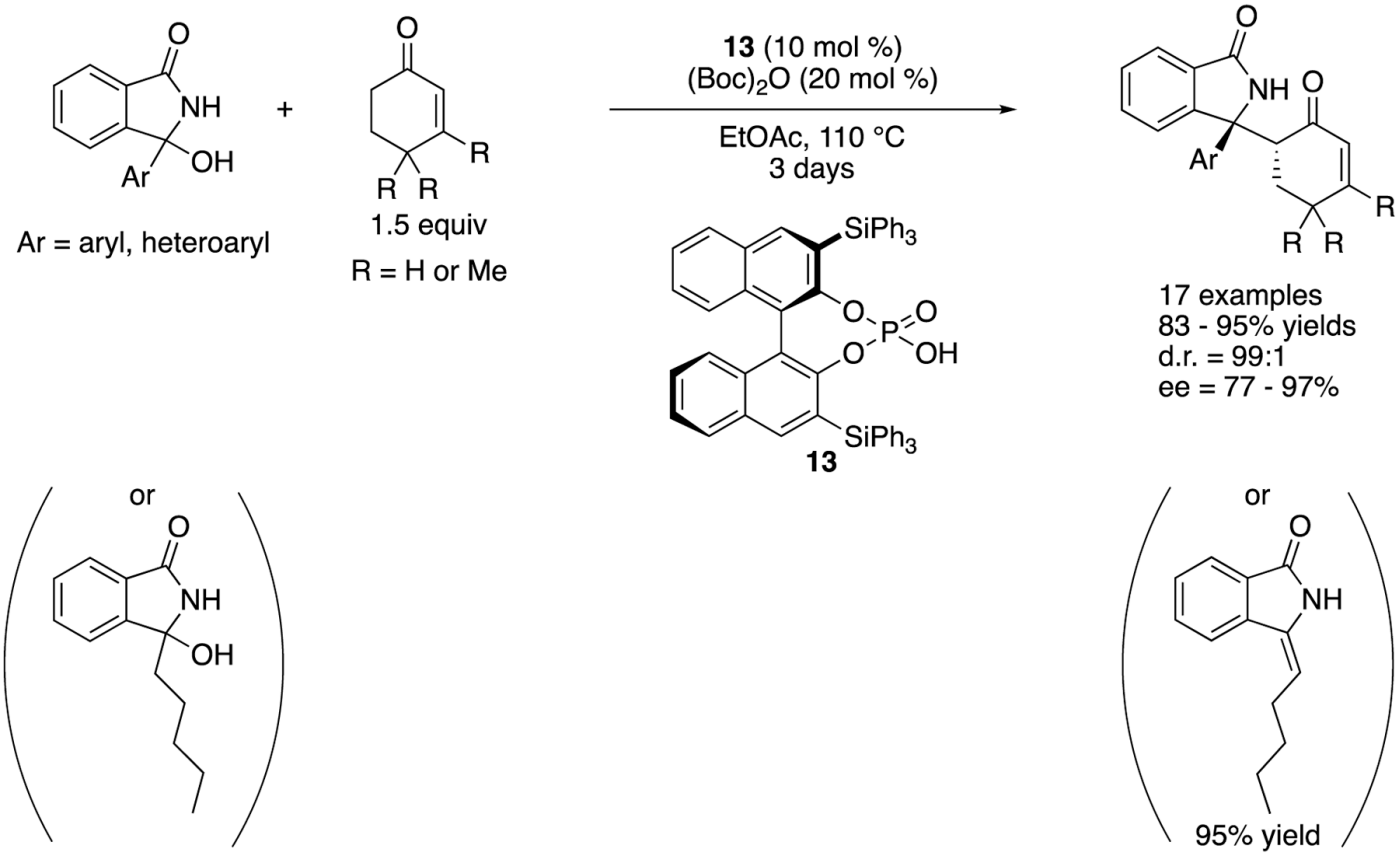
BINOL phosphoric acid-catalyzed Mannich reaction of endocyclic *N*-acyl ketimines.

3-Aryl-3-hydroxyisoindolin-l-ones are often employed as stable precursors for the corresponding endocyclic *N*-carbonyl diaryl ketimines. This class of ketimines is useful synthons to access chiral isoindolin-l-ones that are an important motif found in numerous biologically relevant molecules and natural products. In 2019, Reddy and coworkers reported BINOL phosphoric acid-catalyzed Mannich reaction of endocyclic *N*-acyl ketimines generated in situ from 3-hydroxyisoindolin-l-ones (Scheme 11) [65]. The method provided chiral isoindolin-l-ones bearing adjacent quaternary and tertiary stereogenic centers in 83–95% yields with excellent stereoselectivities despite the high reaction temperature (99:1 diastereoselectivity for all substrates and 77–97% ee). They noted that 3-hydroxy-3-pentylisoindoline-l-one (i.e., a tautomerizable ketimine) provided the corresponding enamide in 95% in 4 h without the desired Mannich product under the optimized condition.

**Scheme 12.**
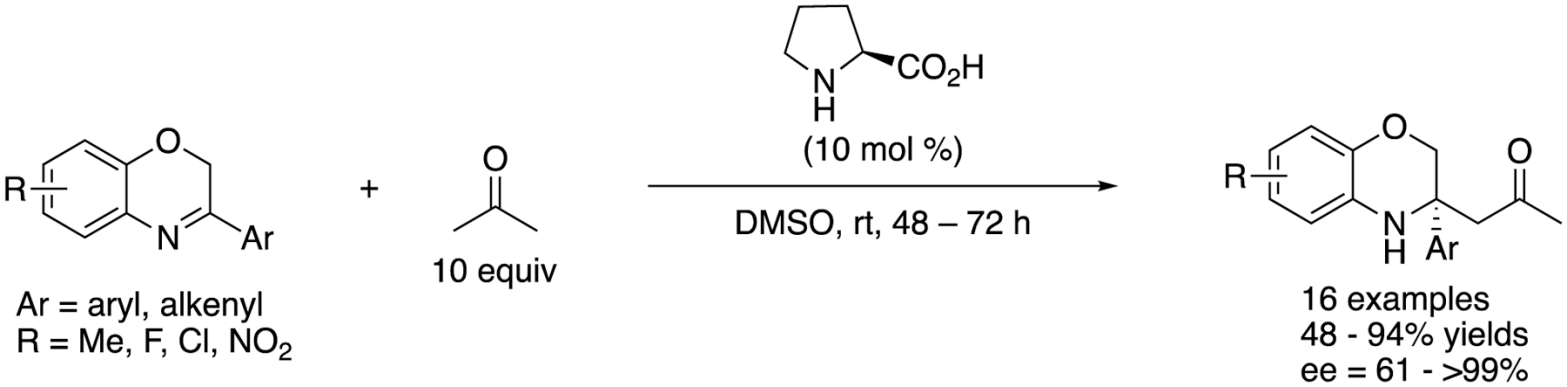
Proline-catalyzed Mannich reaction of 3-substituted-2*H*-l,4-benzoxazines.

In 2013, Wang and coworkers described the proline-catalyzed direct asymmetric Mannich reaction of 3-substituted-2*H*-l,4-benzoxazines (Scheme 12) [66], This work represents the first catalytic asymmetric Mannich reaction of 3,4-dihydro-2*H*-l,4-benzoxazines and provided the *N*-heterocyclic products in 48–94% yields with 61–>99% ee. The authors pointed out that the ring strain in 3,4-dihydro-2H-l,4-benzoxazines contributed to their reactivities. It is still notable that these ketimines bear no electron-withdrawing substituents in sharp contrast to the ketimines discussed earlier in this review. It is worthy of mention that the same transformation catalyzed by wheat germ lipase was reported by Guan, He, and coworkers in 2016 (not shown) [67].

2*H*-Azirines are three-membered ring molecules with a C=N double bond and are the most strained nitrogen unsaturated heterocyclic compounds (selected review; [68]). Their high chemical reactivity is mainly due to their high ring strain that enhances the reactivity of the C=N bond.

**Scheme 13.**
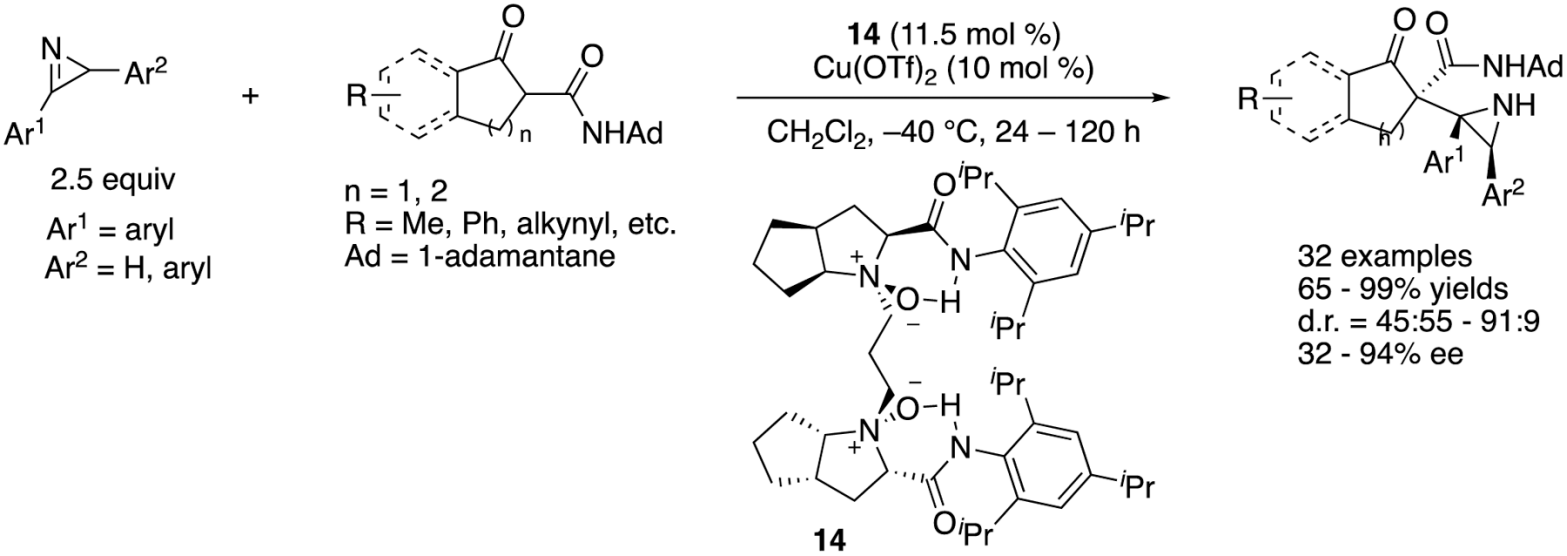
Copper-catalyzed asymmetric Mannich reaction of 2*H*-azirines with β-ketoamides.

In 2018, Lin, Feng, and coworkers developed a copper-catalyzed asymmetric Mannich reaction of 2-*H*-azirines with β-keto amides (Scheme 13) [69]. This represents the first example of the catalytic enantioselective Mannich reaction of 2-*H*-azirines and is one of the two early examples of asymmetric catalytic additions of carbon-based nucleophiles to 2-*H*-azirines (the aza-benzoin reaction of aldehydes with 2-*H*-azirines was reported earlier in the same year) [35], This method employed racemic 2-*H*-azirines; one enantiomer of which preferentially reacted with a chiral Cu-enolate complex, affording the aziridines bearing three contiguous stereogenic centers in 65–99% yields with 45:55–91:9 diastereoselectivities and 32–94% ee.

**Scheme 14.**
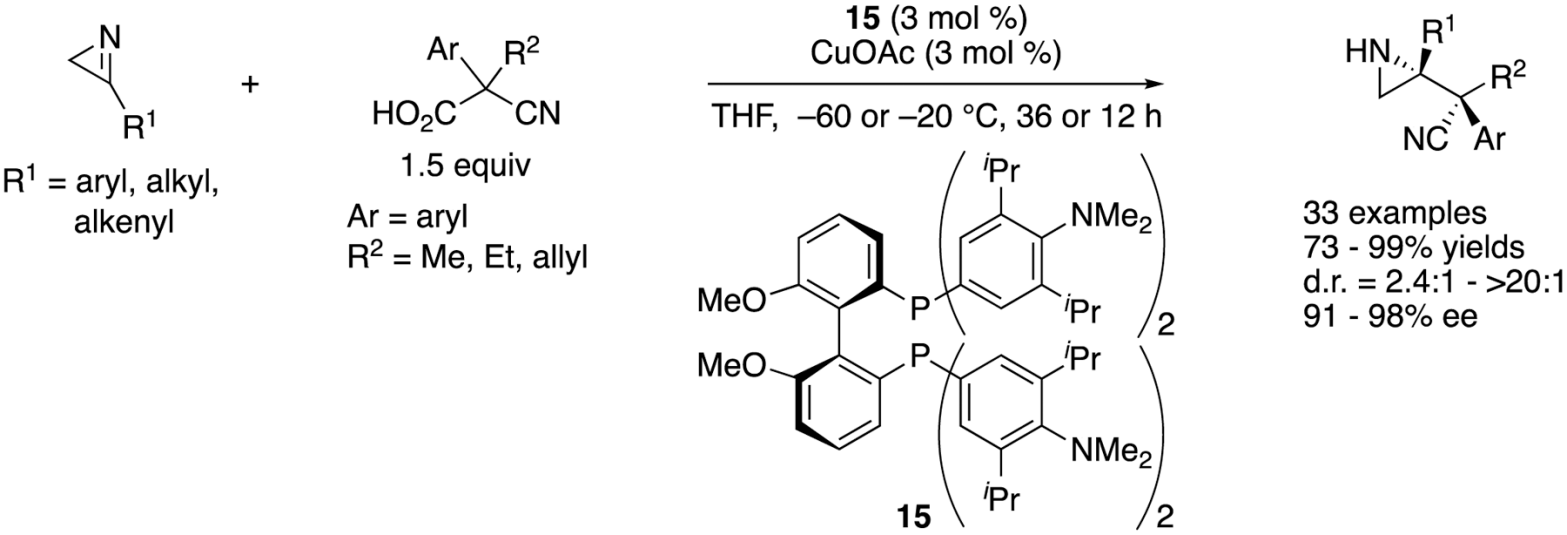
Copper(I)-catalyzed asymmetric decarboxylative Mannich reaction of 2*H*-azirines.

In 2019, Yin and coworkers reported a copper(I)-catalyzed asymmetric decarboxylative Mannich reaction of 2H-azirines (Scheme 14) [70], This method utilized a 2*H*-azirine not only as an electrophile but also as a base to deprotonate a cyanoacetic acid to induce its decarboxylation (i.e., decarboxylative enolization). As such, a reacting electrophile was a protonated 2*H*-azirine whose electrophilicity was enhanced. Importance of this “proton transfer” strategy was successfully demonstrated by showing that a nonprotonated 2*H*-azirine did not react with a corresponding nucleophile under the otherwise identical reaction condition. The reaction scope was very broad and tolerated various substituents on both nucleophiles and electrophiles, affording various enantio-enriched aziridines in 73–99% yields with 2.4:1–>20:1 diastereoselectivities and 91–98% ee. Furthermore, the method worked on a gram-scale reaction with only 2 mol% catalyst loading without any loss in stereoselectivities; albeit, it took 50 h (R^1^ = Ph, R^2^ = Me, Ar = Ph, 91% yield, >20:1 d.r., 97% ee).

**Scheme 15.**
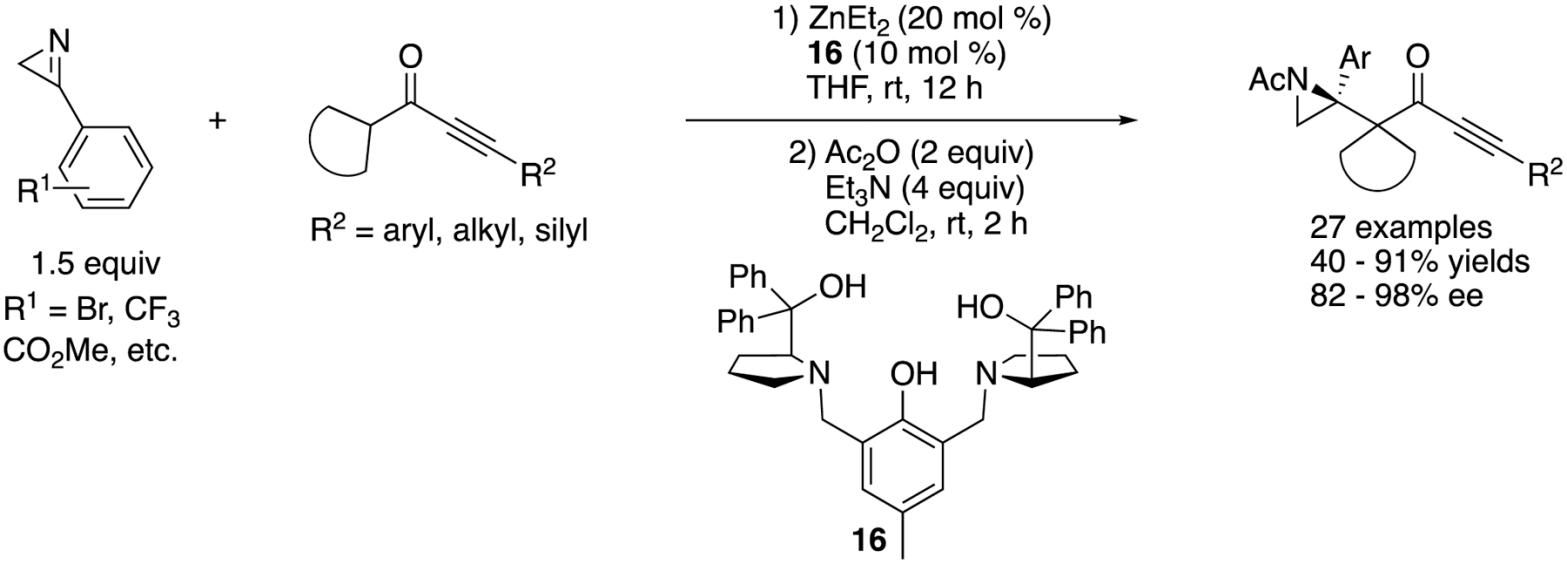
Zn-ProPhenol catalyzed asymmetric Mannich reaction of 2*H*-azirines with alkynyl cycloalkyl ketones.

In 2020, Trost and coworker developed an asymmetric Mannich reaction of 2*H*-azirines with alkynyl cycloalkyl ketones (Scheme 15) [71]. The key to the success was to employ their bimetallic Zn-ProPhenol complex (**16**) that activated both nucleophiles and electrophiles within the same chiral pocket encompassing both Brønsted basic and Lewis acidic sites. The method efficiently provided the complex aziridines in 40–91% yields with 82–98% ee. The authors noted that possible intramolecular hydrogen bonding between the N–H bond of the aziridine moiety and the carbonyl moiety within a product presumably resulted in a chiral center at the nitrogen atom, making the subsequent product characterizations very difficult. Therefore, a sequential N–H bond acetylation of the products was conducted.

#### 2.2.2. Silyl Enol Ethers

**Scheme 16.**
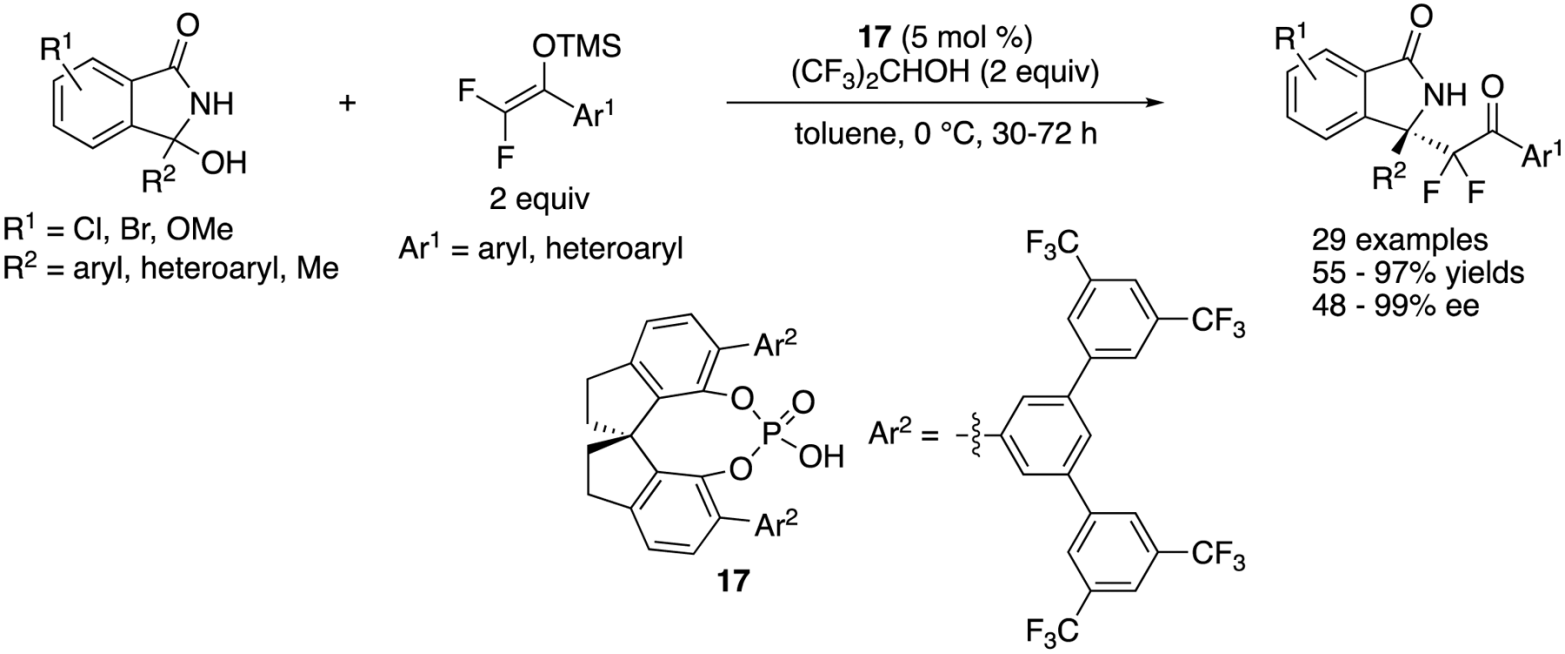
Chiral phosphoric acid-catalyzed Mukaiyama-Mannich reaction of endocyclic *N*-acyl ketimines.

In 2020, Zhang, Ma, and coworkers reported a chiral phosphoric acid-catalyzed Mukaiyama-Mannich reaction of endocyclic *N*-acyl ketimines generated in situ from 3-hydroxyisoindolin-l-ones (Scheme 16) [72]. They employed difluorinated silyl enol ethers as nucleophiles to prepare enantioenriched fluoroalkyl-functionalized isoindolones. During the screening of reaction conditions and chiral phosphoric acid catalysts, they found that (1) the use of hexafluoroisopropyl alcohol as additive was beneficial for both reactivity and enantioselectivity and (2) catalysts bearing trifluoromethylated chiral barriers (Ar^2^) were superior to counterparts having no trifluoromethyl group. As such, they tested a nonfluorinated silyl enol ether (F = H, Ar^1^ = Ph) under the optimized reaction conditions and found it unreactive. The scope of both ketimines and silyl enol ethers were broad, and the Mannich products were obtained in 55–97% yields with 48–99% ee. It is noteworthy that their method tolerated a tautomerizable ketimine (R^2^ = Me), giving the corresponding product (R^1^ = H, Ar^1^ = Ph) in 85% yield with 48% ee (see Scheme 11 for comparison). Furthermore, a gram-scale reaction was successfully demonstrated (R^1^ = H, R^2^ = Ar^1^ = Ph, 98% yield, and 96% ee).

### 2.3. Isatin-Derived Ketimines

3-substituted-3-aminooxindoles are an important structural motif found in numerous natural products and biologically relevant molecules. The catalytic asymmetric Mannich reaction of isatin-derived ketimines provides direct access to enantio-enriched 3-substituted-3-aminooxindoles. Furthermore, isatin-derived ketimines are relatively reactive electrophiles. As such, many catalytic asymmetric Mannich reactions of isatin-derived ketimines have been reported. In light of the excellent review articles that covered up to the end of 2017 [73–75], we discuss herein a few selected examples published after that period (for other selected examples, see: [76–84]).

#### 2.3.1. Direct Mannich

**Scheme 17.**
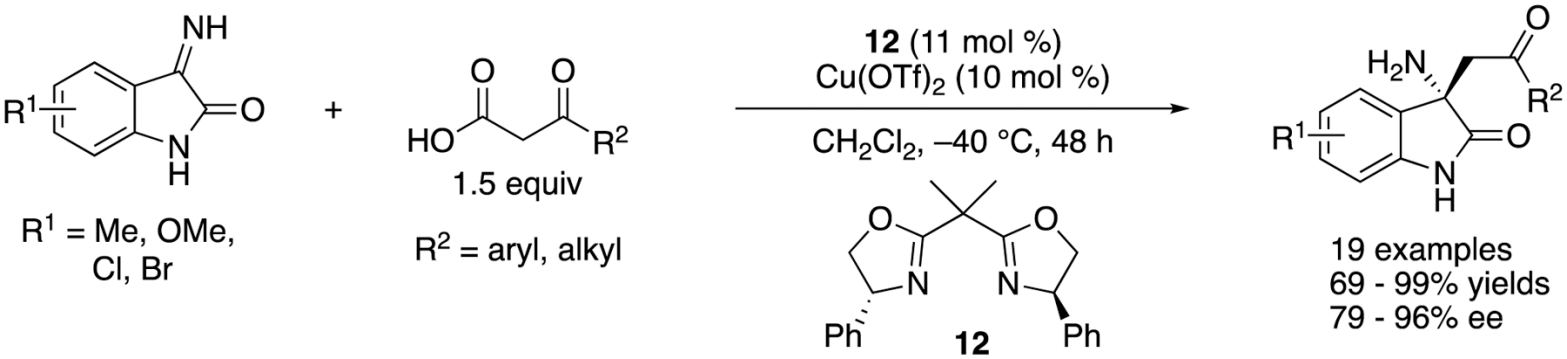
Copper-catalyzed Mannich reaction of *N*-unprotected isatin-derived ketimines.

In 2018, Morimoto, Ohshima, and coworkers reported a decarboxylative Mannich reaction of *N*-unprotected isatin-derived ketimines, which directly afforded enantio-enriched chiral oxindoles bearing primary amines at their 3-postions (Scheme 17) [85]. The method that can accommodate substrates that have no protecting groups are more step- and atom-economical than the counterparts that require them [86–88], The method tolerated various ketoacids, affording the Mannich products in 69–99% yields with 79–96% ee. Their preliminary mechanistic study indicated that the addition of a ketoacid to a ketimine preceded the decarboxylation process with their catalytic system.

**Scheme 18.**
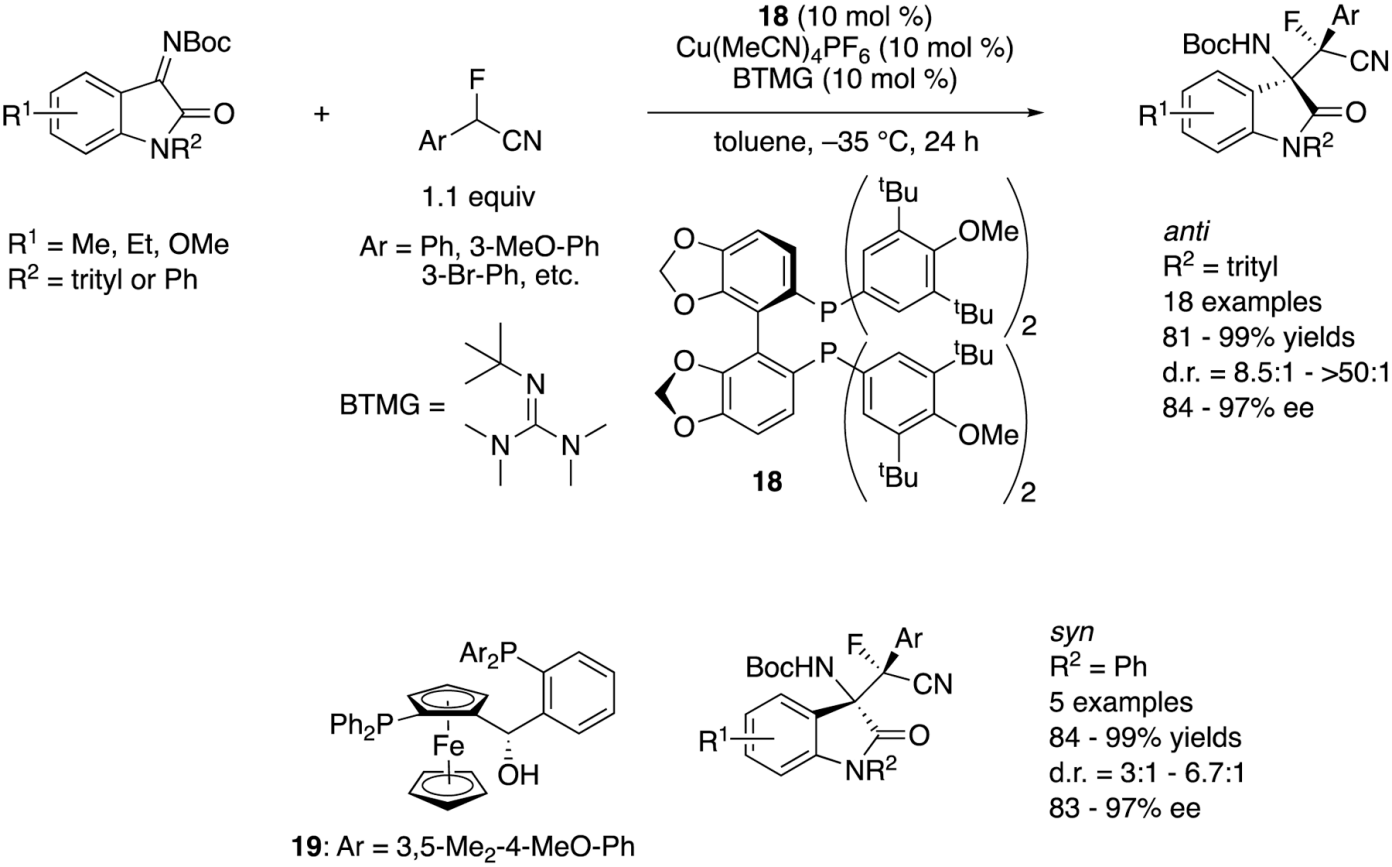
Stereodivergent asymmetric Mannich reaction of α-fluoronitriles with isatin-derived ketimines.

In 2019, Wolf and coworkers developed a copper-catalyzed stereodivergent asymmetric Mannich reaction of isatin-derived ketimines and α-fluoro-α-arylnitriles (Scheme 18) [89]. A chiral cuprous keteniminate complex generated from α-fluoro-α-arylnitrile, a chiral copper catalyst, and BTMG was proposed as a nucleophile. The Segphos (**18**)-copper complex provided anti diastereomers in 81–99% yields with 8.5:1–>50:1 diastereoselectivities and 84–97% ee. On the other hand, the Taniaphos (**19**) complex afforded syn diastereomers in 84–99% yields with 3:1–6.7:1 diastereoselectivities and 83–97% ee. The switching of diastereoselectivities was successfully demonstrated by choosing a proper combination of the chiral ligand and the isatin *N*-protecting group (trityl or phenyl). Furthermore, a gram-scale reaction (R^1^ = H, R^2^ = trityl, Ar = Ph, 99% yield, 12.7:1 d.r., 90% ee) was successfully carried out without compromising yield and stereoselectivities.

**Scheme 19.**
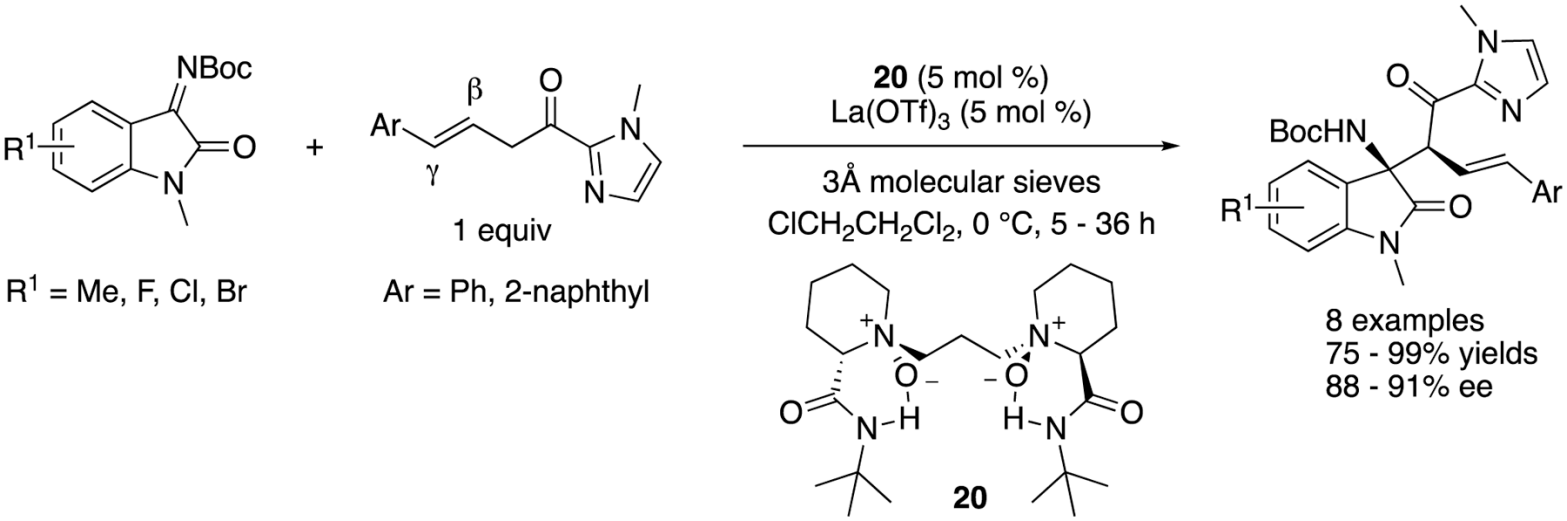
Chiral La-catalyzed Mannich reaction of isatin-derived ketimines with β,γ-unsaturated 2-acyl imidazoles.

In 2020, Liu, Feng and coworkers described several Lewis acid-catalyzed enantioselective transformations of (β,γ-unsaturated 2-acyl imidazoles; the study of which included ketimines as electrophiles (Scheme 19) [90], After the screening of the reaction conditions, the catalyst complex generated from La(OTf)3 and (*S*)-pipecolic acid-derived ligand was found to promote the Mannich reaction of isatin-derived ketimines and β,γ-unsaturated 2-acyl imidazoles, delivering the desired β-amino 2-acyl imidazoles as single regio- and diastereo-isomers in 75–99% yields and 88–91% ee. The pyrazolinone-derived ketimines were also demonstrated to be good substrates for their method (not shown).

#### 2.3.2. Silyl Enol Ethers

**Scheme 20.**
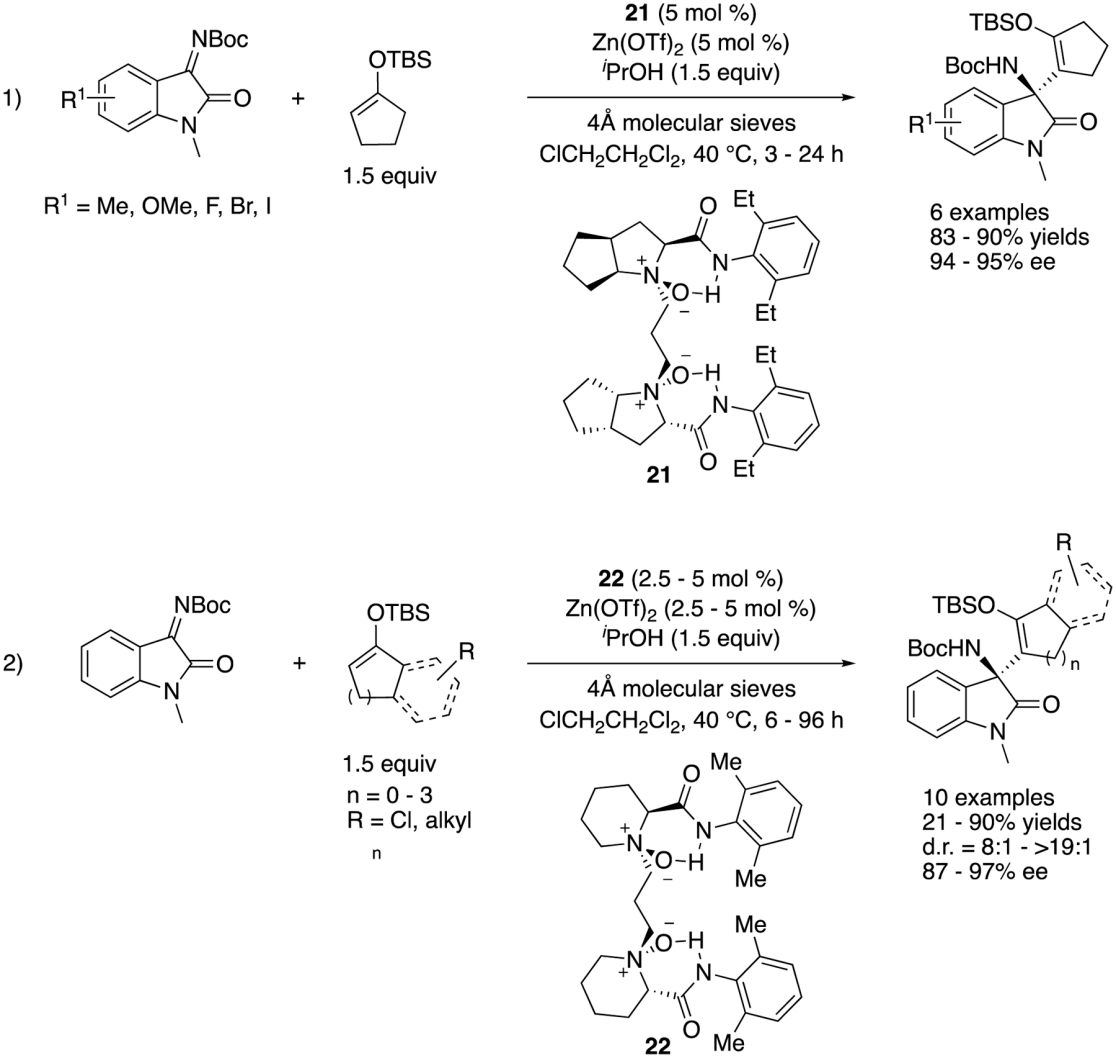
Tandem α-alkenyl addition/proton shift reaction of silyl enol ethers with ketimines.

In 2019, Feng and coworkers reported a serendipitously discovered tandem α-alkenyl addition/proton shift reaction of silyl enol ethers and ketimines catalyzed by chiral *N,N*′-dioxide/Zn(II) complexes (Scheme 20) [91]. After the optimization of reaction conditions, dioxide **21** was found to be optimal for a series of isatin-derived ketimines (1), giving the products in 83–90% yields with 94–95% ee. A gram-scale reaction (R^1^ = H) was successfully demonstrated without compromising yield and stereoselectivity (86% yield and 95% ee). With respect to the scope of silyl enol ethers, dioxide **22** turned out best and provided the products in 21–90% yields with 87–97% ee and 8:1–>19:1 diastereoselectivities (for racemic α′-substituted cyclic silyl enol ethers that required 10 mol% catalyst loadings). The pyrazolinone-derived ketimines were also demonstrated to be good substrates for the method (not shown). Their preliminary mechanistic study strongly supported that the Mukaiyama-Mannich addition intermediates got protonated by isopropanol (additive) before the subsequent silyl shift, giving the corresponding β-amino silyl enol ethers.

### 2.4. Acyclic Ketimines Bearing Electron-Withdrawing Groups and/or Alkynes

Ketimines substituted with esters, perfluorinated alkyl, or alkyne groups are relatively good electrophiles, and thus, enolates with a range of different nucleophilicities (i.e., different catalytic methods) were reported for their Mannich reactions. Nonetheless, some examples presented in this section demonstrate that seemingly subtle differences in the ketimine structures substantially affected chemical yields and stereoselectivities, highlighting the challenging nature of ketimine Mannich reactions. On the other hand, some ketimines presented herein were creatively designed so as to not only circumvent the inherent problems of unmodified ketimines but also to provide synthetically and medicinally important chiral building blocks.

#### Direct Mannich

**Scheme 21.**
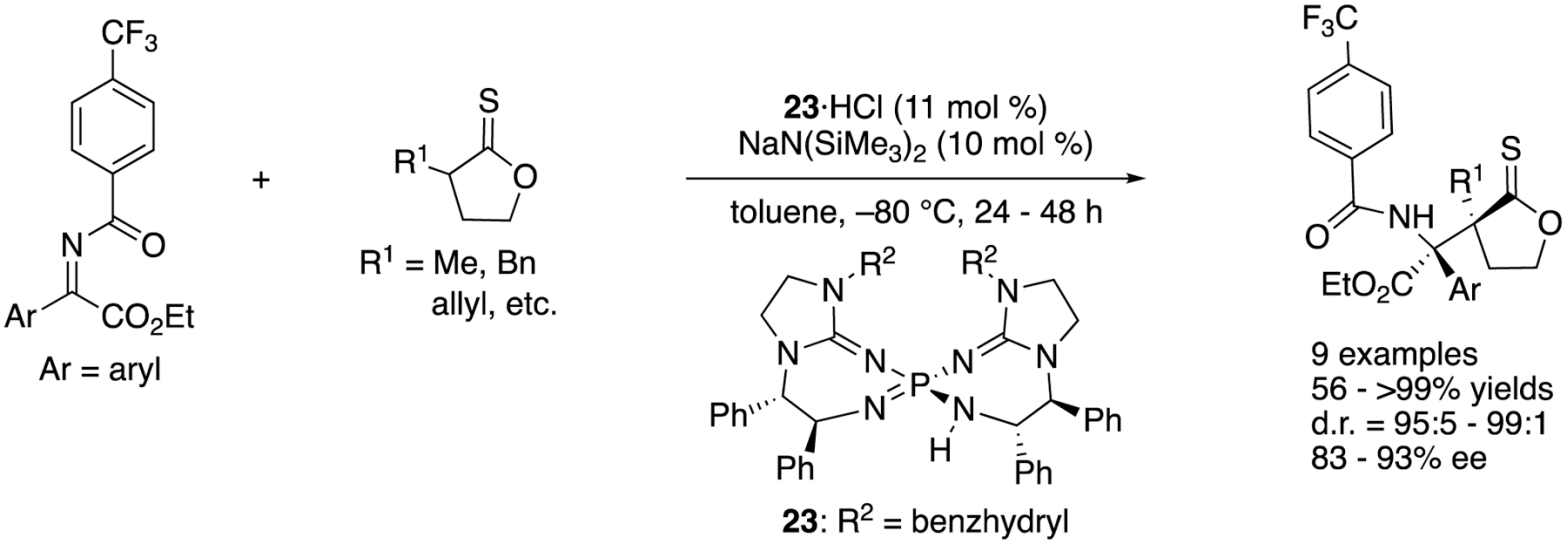
Chiral organosuperbase-catalyzed direct Mannich reaction of ketiminoesters.

In 2016, Terada and coworkers developed a novel asymmetric direct Mannich reaction of ketiminoesters with thionolactones using bis(guanidino)iminophosphorane **23** as a chiral organosuperbase catalyst (Scheme 21) [92], Thionolactones were identified as suitable nucleophiles, while a corresponding lactone did not undergo the reaction. It was presumed that a lactone was not acidic enough to get enolized by the base catalyst. With respect to the electrophiles, the substituent on the benzoyl protecting group was found crucial for both chemical yield and diastereoselectivity. For example, a corresponding methoxy-substituted ketiminoester (CF_3_ = OMe) provided the product in much lower yield and selectivity (Ar = *p*-tol: for CF_3_, 93% yield, d.r. = 95:5; for OMe, 35% yield, d.r. = 88:12). Under the optimized conditions, the method provided the products with vicinal quaternary stereogenic centers in 56–>99% yields with 95:5–99:1 diastereoselectivities and 83–93% ee.

**Scheme 22.**
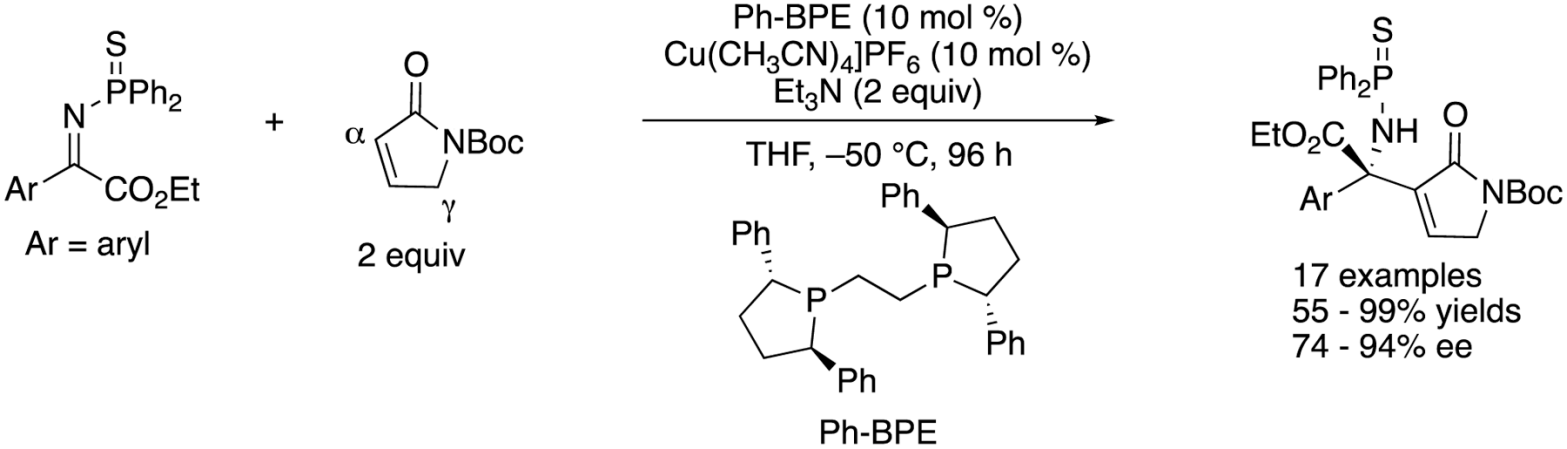
Direct enantioselective Mannich reaction of α,β-unsaturated γ-butyrolactam with ketiminoesters.

As part of their longstanding interests in the soft Lewis acid/hard Bronsted base cooperative catalysis, in 2016 Kumagai, Shibasaki, and a coworker developed the direct catalytic asymmetric Mannich reaction of an α,β-unsaturated γ-butyrolactam with ketiminoesters (Scheme 22) [93]. This method was found to give the α-addition products (aza-Morita-Baylis-Hillman type products) through the regioselective α-addition of the dienolate intermediate followed by isomerization of a double bond. This work represents the first α-addition of α,β-unsaturated γ-butyrolactams to ketimines under the asymmetric catalysis conditions. The interaction of the soft Lewis acidic copper catalyst and soft Lewis basic thiophosphinoyl protecting group of ketimines was suggested crucial for the success of this Mannich reaction, because a corresponding phosphinoyl protected ketiminoester did not react under the otherwise identical reaction conditions. These observations were consistent with their earlier works on the soft Lewis acid/hard Brønsted base cooperative catalysis (vide infra). The method accommodated 17 ketiminoesters and provided the β-amino carbonyl products in 55–99% yields with 74–94% ee. A gram-scale reaction was demonstrated without any loss in yield or ee (Ar = Ph, 99% yield, 91% ee).

**Scheme 23.**
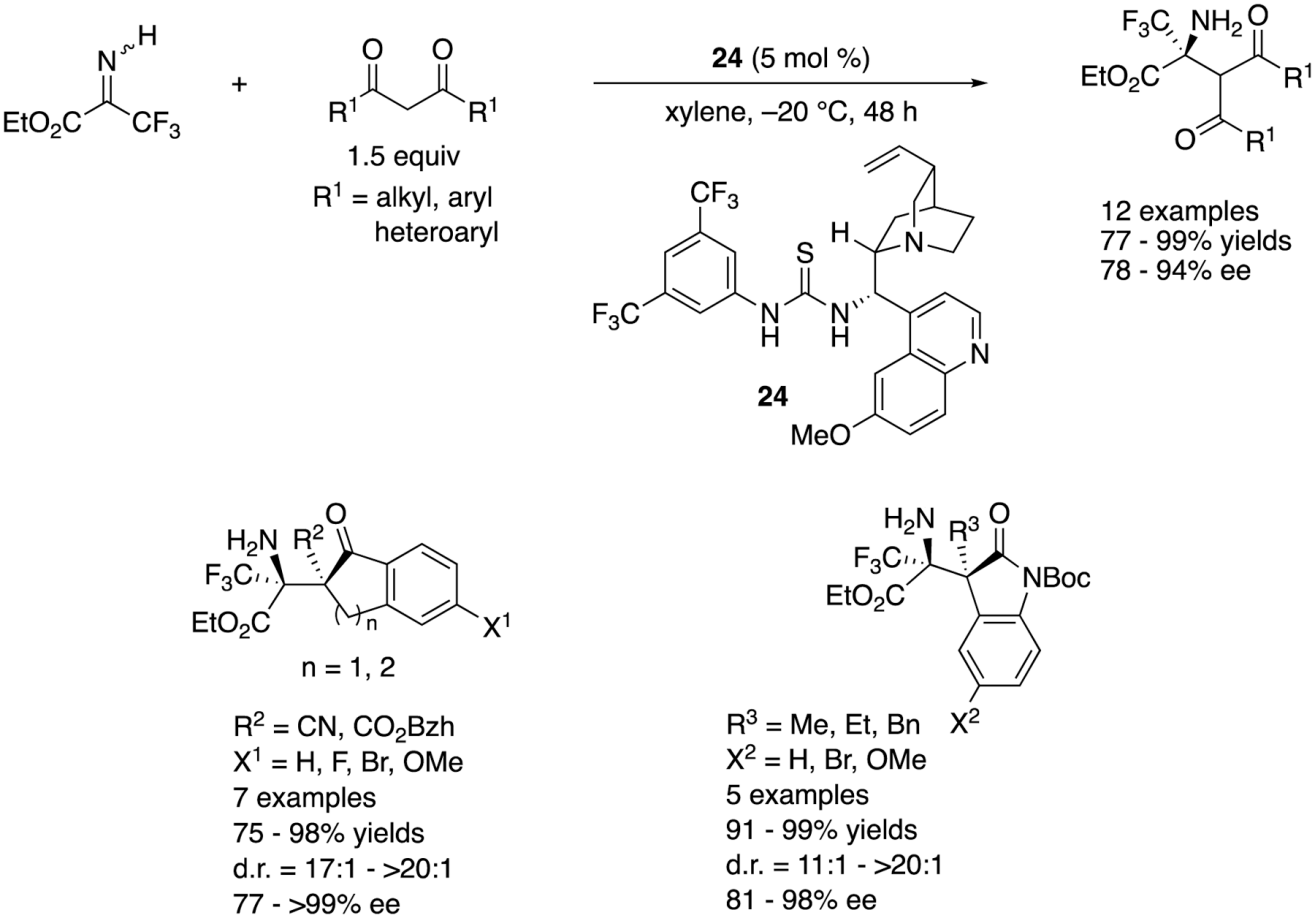
Direct catalytic Mannich reaction with an *N*-unprotected trifluoromethyl ketiminoester.

As part of their efforts in the development of the transformations employing *N*-unprotected imines/ketimines, in 2017 Morimoto, Ohshima, and coworkers reported a direct enantioselective Mannich reaction with an *N*-unprotected trifluoromethyl ketiminoester (Scheme 23) [94]. While unprotected *N*-H ketimines generally have limited stabilities, they often mitigate problems associated with the *E*/*Z* isomerism in the asymmetric catalysis reactions. Under the optimized conditions, their method tolerated malonates, cyclic (3-keto-nitriles, esters, and oxindoles (conducted at 0 °C instead of −20 °C), providing the corresponding products in 77–99% yields with 78–94% ee, 75–98% yields with 17:1–>20:1 diastereoselectivities and 77–>99% ee, and 91–99% yields with 11:1–>20:1 diastereoselectivities and 81–98% ee, respectively.

**Scheme 24.**
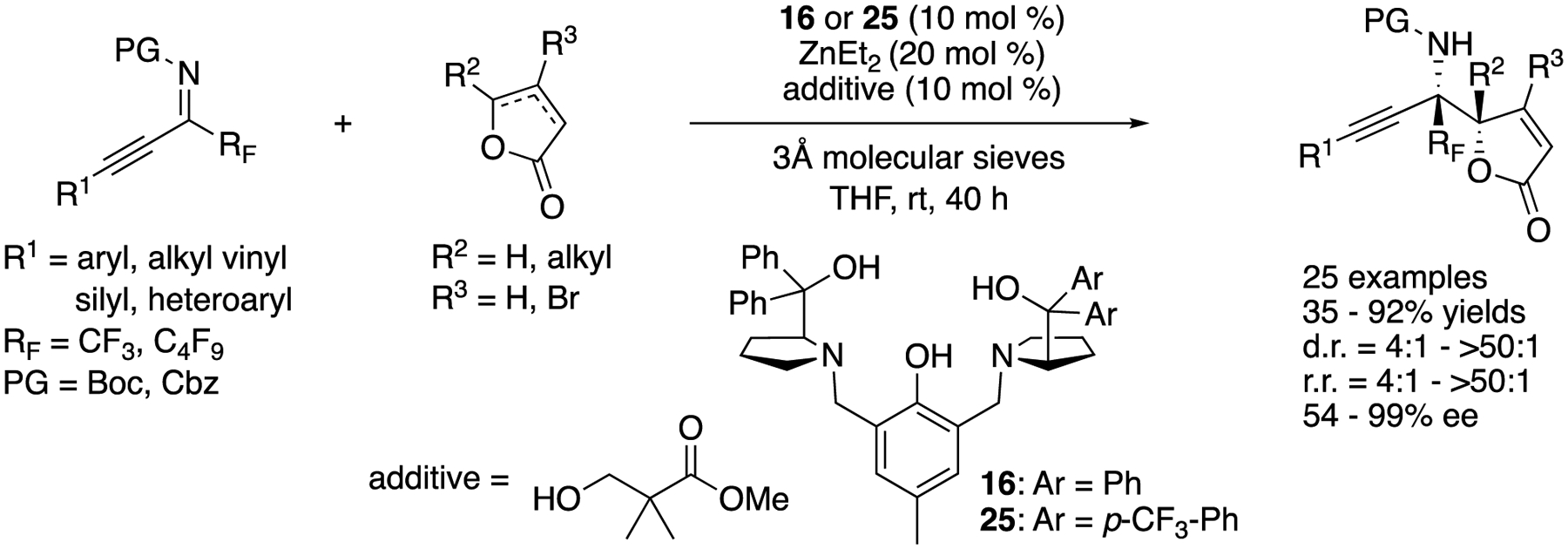
Direct catalytic asymmetric vinylogous Mannich reaction of polyfluorinated alkynyl ketimines.

In 2018, Trost and coworkers developed a Zn-ProPhenol catalyzed asymmetric Mannich reaction of α,β- and β/γ-butenolides with perfluoroalkyl alkynyl ketimines (Scheme 24) [95]. The method provided vinylogous products bearing two contiguous tetrasubstituted stereogenic centers. This represents the first successful use of ketimines in the ProPhenol Mannich process. They noted the salient features of perfluoroalkyl alkynyl ketimines; (1) a fluorinated group makes ketimines more electrophilic; (2) an alkyne is sterically much less demanding and its higher s character should further enhance the electrophilicity of ketimines; and (3) the increased steric differences between alkynyl and perfluoroalkyl groups would give ketimines in a single diastereomeric form, which is critical for high stereoselectivities. The reaction scope was very broad, providing the medicinally important (i.e., perfluoroalkyl groups) and synthetically versatile (i.e., alkynes) Mannich products in 35–92% yields with 4:1–>50:1 diastereoselectivities, 4:1–>50:1 regioselectivities, and 54–99% ee.

**Scheme 25.**
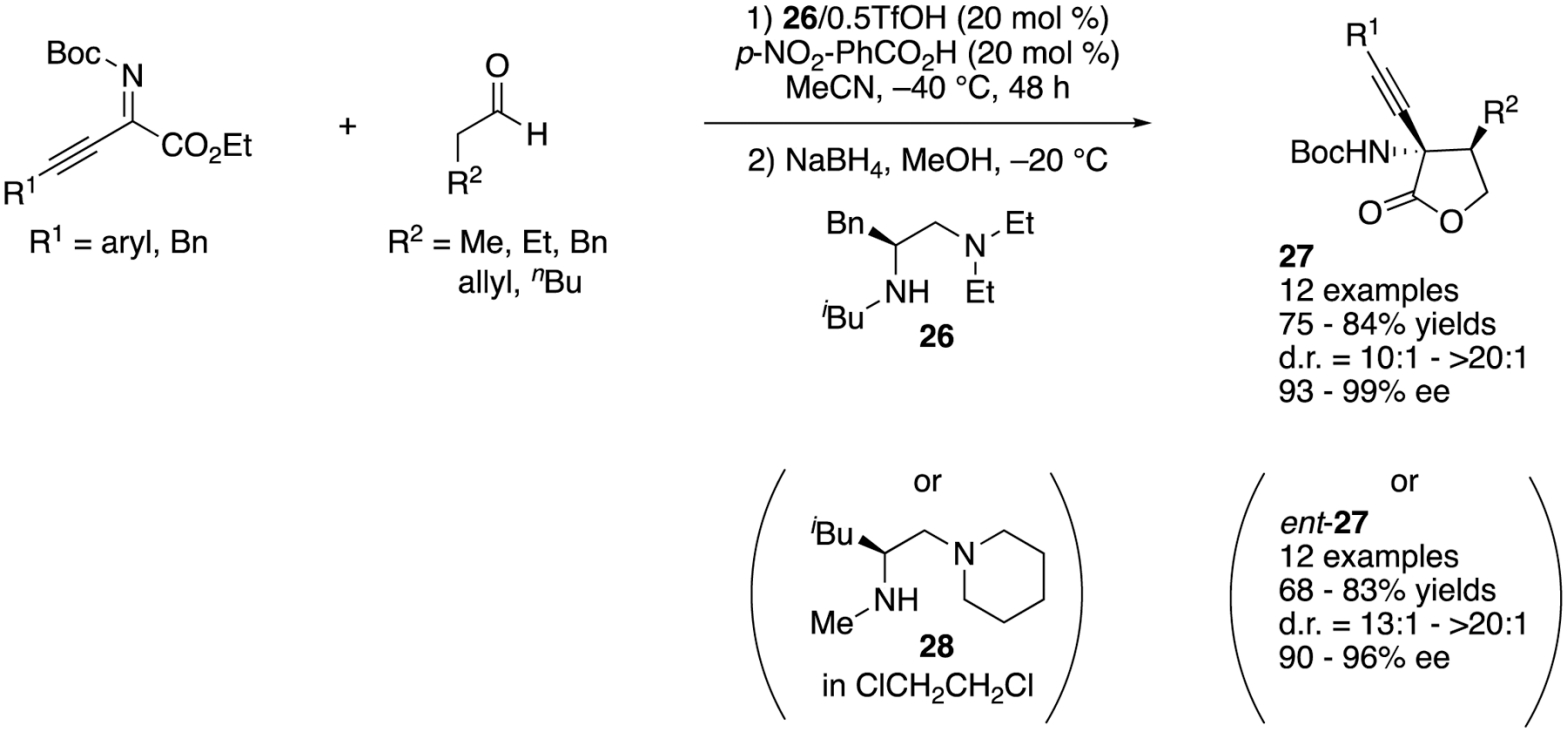
Enantiodivergent Mannich reaction of alkynyl ketiminoesters catalyzed by chiral amines.

Chiral amine catalysts derived from natural amino acids have proven highly effective for a wide range of enantioselective transformations. However, enantiomers of natural amino acids are not always readily available. As such, in 2019, Lan, Shao, and coworkers described their strategy to address this issue, which was “enantiodivergence” via minimal modification of a chiral amine catalyst (Scheme 25) [96]. They successfully demonstrated this strategy by applying their catalysts to the Mannich reaction of alkynyl ketiminoesters. By switching catalyst **26** and MeCN to **28** and dichloroethane, the corresponding enantiomers were obtained in almost the same yields and stereoselectivities. They also demonstrated that the same was true for a range of alkynyl trifluoromethyl-, isatin-derived-, and pyrazolinone-derived ketimines (not shown).

**Scheme 26.**
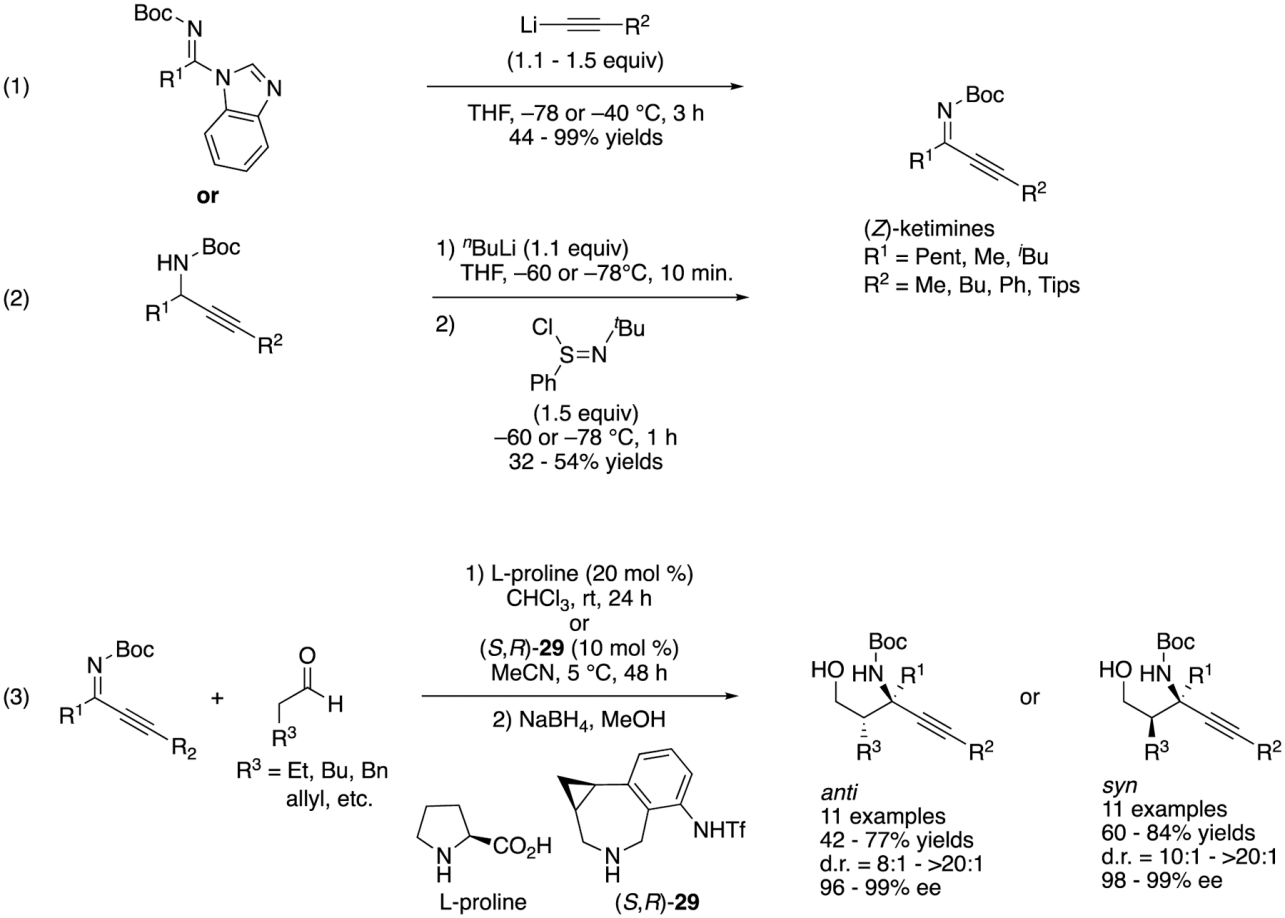
Chiral amine-catalyzed Mannich reactions of (*Z*)-alkynyl alkyl ketimines.

Ketimines bearing two structurally similar substituents exist as an inseparable *E/Z* diastereomeric mixture and thus are rarely utilized in the asymmetric catalysis methods in contrast to ketimines that are available in a single diastereomeric form. Dialkyl ketimines are a prototypical example of such isomeric ketimine mixtures. They are also relatively easy to tautomerize to enamines (especially ones protected with Boc). In 2021, Kano, Maruoka, and coworkers reported a solution to this classical yet persisting problem, which is to utilize alkynyl alkyl ketimines as synthetic equivalents of dialkyl ketimines. The alkynyl groups in the reaction products can be readily reduced to the corresponding alkyl groups through simple hydrogenation. Furthermore, the steric size differences between alkynyl and alkyl groups are large enough to give alkynyl alkyl *N*-protected ketimines in a single diastereomeric form, which in turn makes them easy to differentiate by a chiral catalyst. As discussed above, alkynyl-substituted ketimines are more reactive than the corresponding alkyl counterparts due to their electronegative sp hybridized carbon atoms and sterically small triple bonds. These advantageous properties of alkynyl alkyl ketimines certainly outweigh the cost of an extra hydrogenation step. Nonetheless, *N*-Boc alkynyl alkyl ketimines are hardly accessible in a single diastereomeric form through dehydrative condensation reaction. Therefore, they developed the synthesis of *N*-Boc-protected alkynyl alkyl ketimines ((1) and (2), Scheme 26) and then proceeded to evaluate them for chiral amine-catalyzed Mannich reactions (3) [97]. (Z)-Alkynyl alkyl ketimines underwent chiral amine-catalyzed Mannich reaction smoothly with aldehyde nucleophiles. Their method is stereodivergent; proline provided anti products in 42–77% yields with 8:1–>20:1 diastereoselectivities and 96–99% ee, and phenylcyclopropane-based amine **29** afforded syn counterparts in 60–84% yields with 10:1–>20:1 diastereoselectivities and 98–99% ee. They successfully demonstrated the hydrogenation of the Mannich products, providing chiral amines bearing two structurally similar alkyl groups that are otherwise very difficult to prepare in an enantio-enriched form (not shown).

## 3. Unmodified Ketimines in the Mannich Reaction

Unless the ketimine activating groups are part of the desired products (e.g., CF_3_, isatin), they need to be transformed adequately or removed from the Mannich products, which adds steps that would not have been necessary otherwise (vide supra). Therefore, arguably, the development of the Mannich reaction with unmodified ketimines would be highly beneficial from the viewpoints of both atom and step economies [86–88].

### 3.1. Silyl Ketene Acetals

**Scheme 27.**
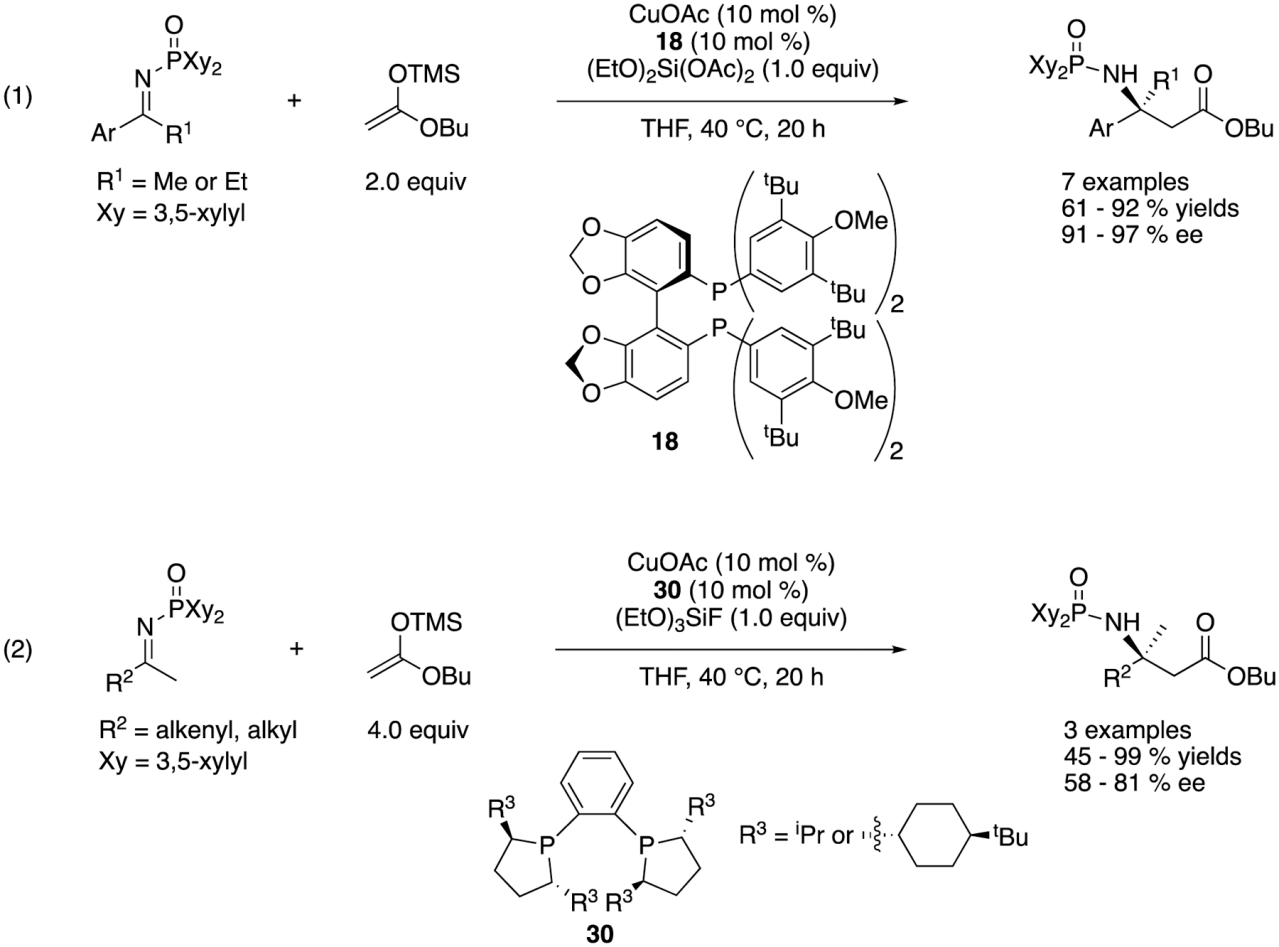
The first asymmetric catalytic Mannich reaction of unmodified ketimines.

In 2007, Kanai, Shibasaki, and coworkers disclosed the first catalytic enantioselective Mannich reaction of unmodified ketimines using a preformed silyl ketene acetal and chiral copper complexes (Scheme 27) [98]. In these reactions, *N*-di(3,5-xylyl)phosphinoyl ketimines were employed, and a highly nucleophilic copper enolate was generated from a silyl ketene acetal and a chiral copper complex through transmetalation. They attempted to accelerate the presumable turnover-limiting catalyst regeneration step from the intermediate copper amide product by using an electrophilic silicon species, (EtO)_2_Si(OAc)_2_ or (EtO)_3_SiF as a trapping agent and successfully improved the yield. While optimal chiral ligands for aromatic and aliphatic ketimines turned out to be different, the method tolerated both classes of ketimines very well, giving the β,β-disubstituted amino acid equivalents in 61–92% yields with 91–97% ee and 45–99% yields with 58–81% ee, respectively, While they mentioned that the nucleophile scope was limited to acetate donors since α-substituted enolates did not undergo the addition reaction, this work still remains as the state-of-the-art catalytic enantioselective Mannich reaction between unmodified ketimines and acetate donors.

**Scheme 28.**
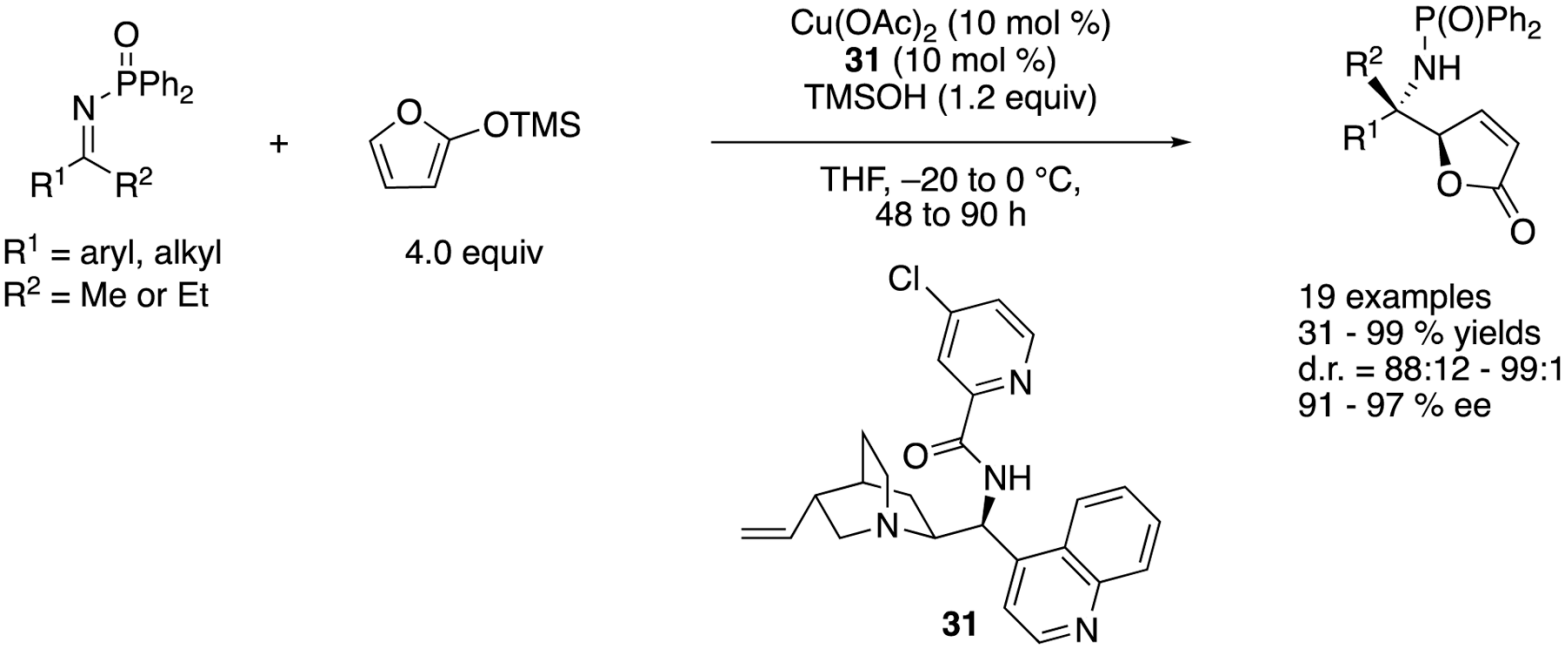
Chiral copper(II) complex-catalyzed anti-selective vinylogous ketimine Mannich reaction.

In 2013, Nakamura and coworkers reported a diastereo- and enantio-selective vinylogous Mannich reaction of *N*-diphenylphosphinoyl ketimines and 2-(trimethylsiloxy)furan catalyzed by a chiral copper(II) complex (Scheme 28) [99], They proposed that a nucleophilic chiral copper dienolate complex generated from 2-(trimethylsiloxy)furan was an actual nucleophilic species. Cinchona alkaloid-derived amide **31** was found to be an optimal ligand, and its copper (II) complex tolerated both aromatic and aliphatic ketimines, giving the products 31–99% yields with 88:12–99:1 diastereoselectivities and 91–97% ee.

### 3.2. Reductive Mannich

**Scheme 29.**
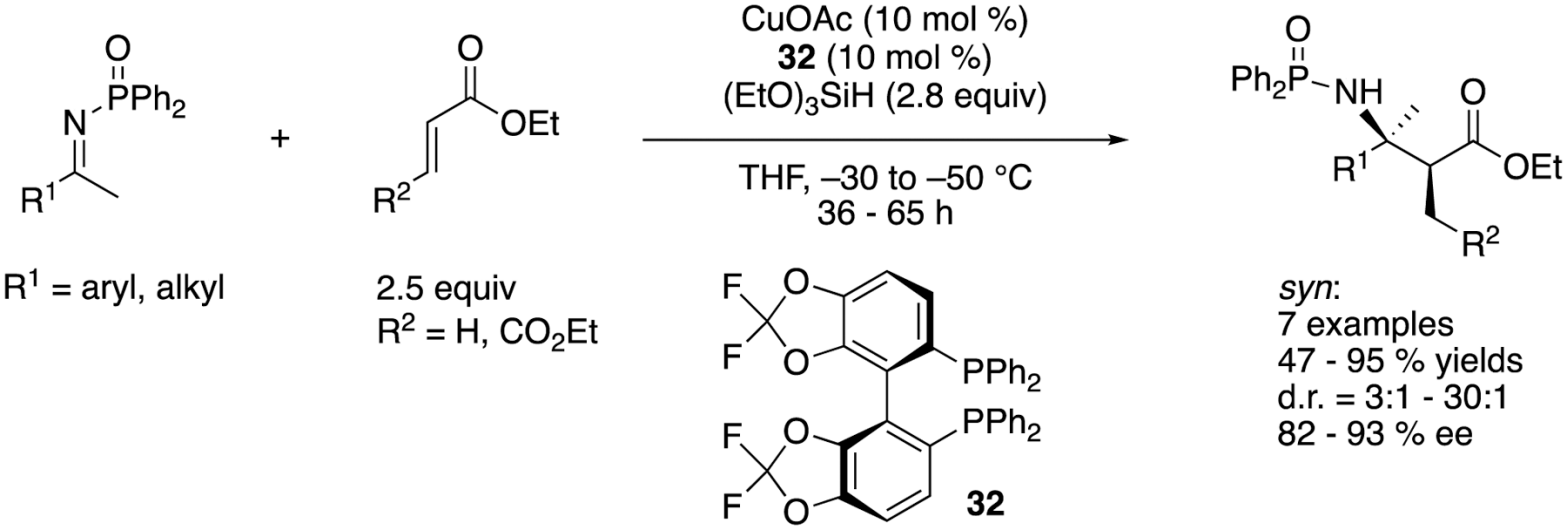
State-of-the-art enantioselective propionate ketimine Mannich reaction.

α,β,β-trisubstituted (β^2,3,3^) amino acids are among the important building blocks for a wide variety of natural products, medicinally relevant molecules, and mimics of protein structural motifs. The ketimine Mannich reaction of α-substituted enolates provides direct access to α,β,β-trisubstituted amino acids from readily available carbonyl compounds. In 2008, Kanai, Shibasaki, and coworkers described their efforts to significantly expand their previous ketimine Mannich reaction (Scheme 27) that was limited to acetate nucleophiles (Scheme 29) [100]. Since they found that the copper enolate generation from a corresponding silicon enolate through transmetalation was the rate-limiting step for their method, they attempted to increase the concentration of active copper enolates by the conjugate addition of Cu-based nucleophiles to α,β-unsaturated esters. Their reductive enolate formation approach turned out to be fruitful and afforded the Mannich products in 47–95% yields with 3:1–30:1 diastereoselectivities and 82–93% enantiomeric excesses. This work is not only the first example of but also state of the art for the Mannich reaction between nonactivated ketimines and propionate nucleophiles.

**Scheme 30.**
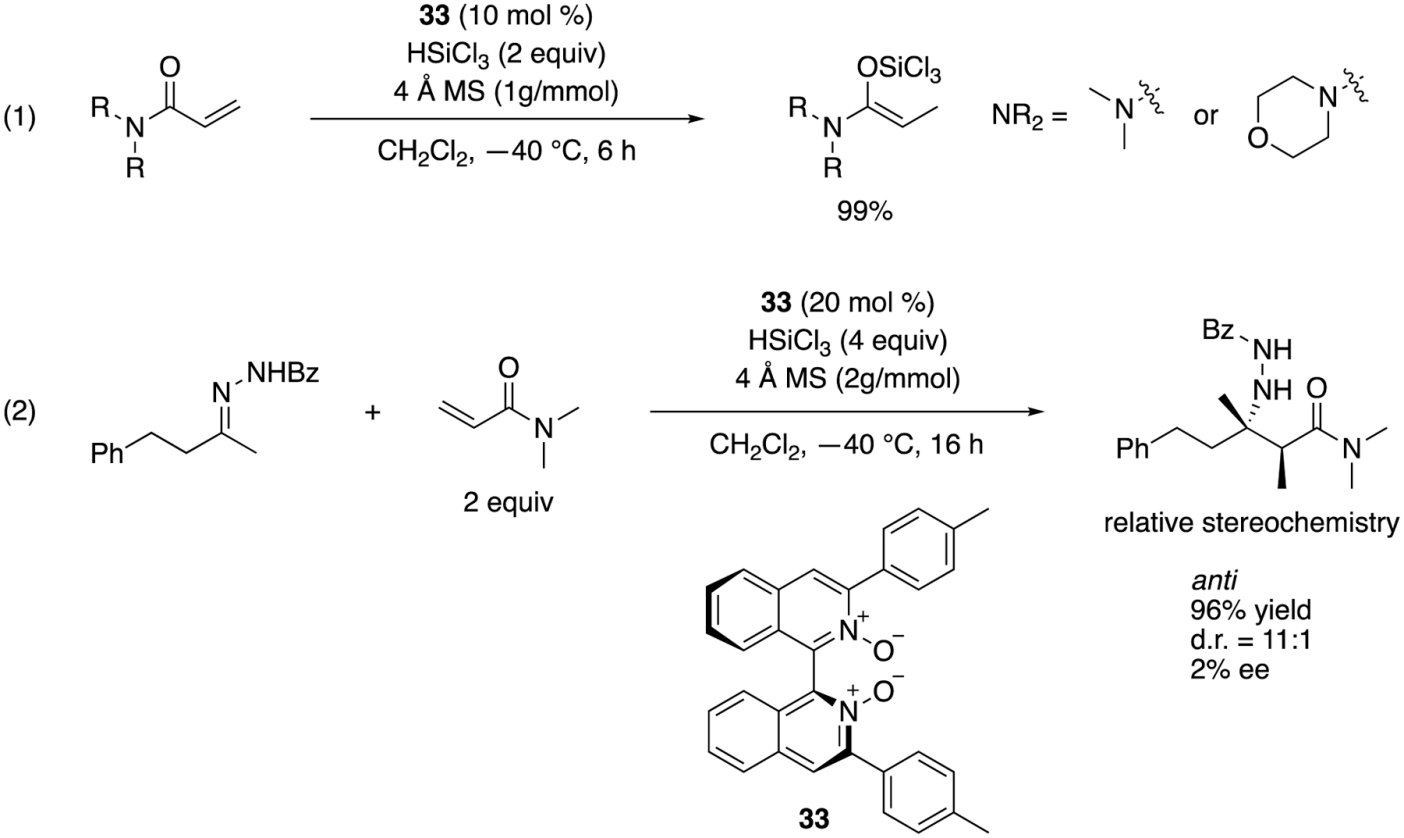
Anti-selective propionate ketimine Mannich reaction.

Reep and Takenaka developed a simple method to generate exceedingly reactive *O*-trichlorosilyl-*N*-*O*-ketene acetals from corresponding acrylamides and HSiCl_3_ under the Lewis base catalysis conditions as an approach to the propionate ketimine Mannich reaction (Scheme 30) [101]. At the outset of their study, they found that the reported reductive method for (α,β-unsaturated ketones [102,103] quantitatively reduced dimethylacrylamide in the presence of benzaldehyde, but no aldol products formed, although they fully reproduced the reported reductive aldol reaction. Since the D_2_O quenching of the reaction did not incorporate the detectable amount of D atom in a resulting propionamide, they hypothesized that this hitherto unknown *O*-trichlorosilyl-*N*-*O*-ketene acetal got rapidly protonated by a small amount of HC1 intrinsic to HSiCl_3_. After screening various proton scavengers, they found that activated 4 Å molecular sieves (MS) were optimal. Their success could be attributed to the “sieve effect” since 3 and 5 Å MS gave the similar results but not 10 Å MS. These findings highlighted the exceedingly higher nucleophilicity of *O*-trichlorosilyl-*N*-*O*-ketene acetal than ketone-derived counterparts that underwent aldol reactions in the absence of proton scavengers. They focused on α-mono-substituted *Z(O)-N, O*-ketene acetals, because the high nucleophilicity is presumably derived from their nitrogen lone-pairs that can donate electron density to the enol units. The corresponding α,α-disubstituted ketene acetals lose this electronic benefit due to the allylic strain that pushes their nitrogen lone-pairs out of conjugation with the enol functionality [104–106]. They tested the reductive method for an aliphatic ketone-derived benzoylhydrazone and found that the desired product formed in 96% yield with 11:1 diastereoselectivity and only 2% ee. Since their method provided an anti diastereomer, it could possibly be developed into a complementary method to Shibasaki’s syn-selective propionate ketimine Mannich reaction (Scheme 29). The relative stereochemistry of the Mannich product was established by the X-ray analysis of the product crystal [107].

### 3.3. Enamine

**Scheme 31.**
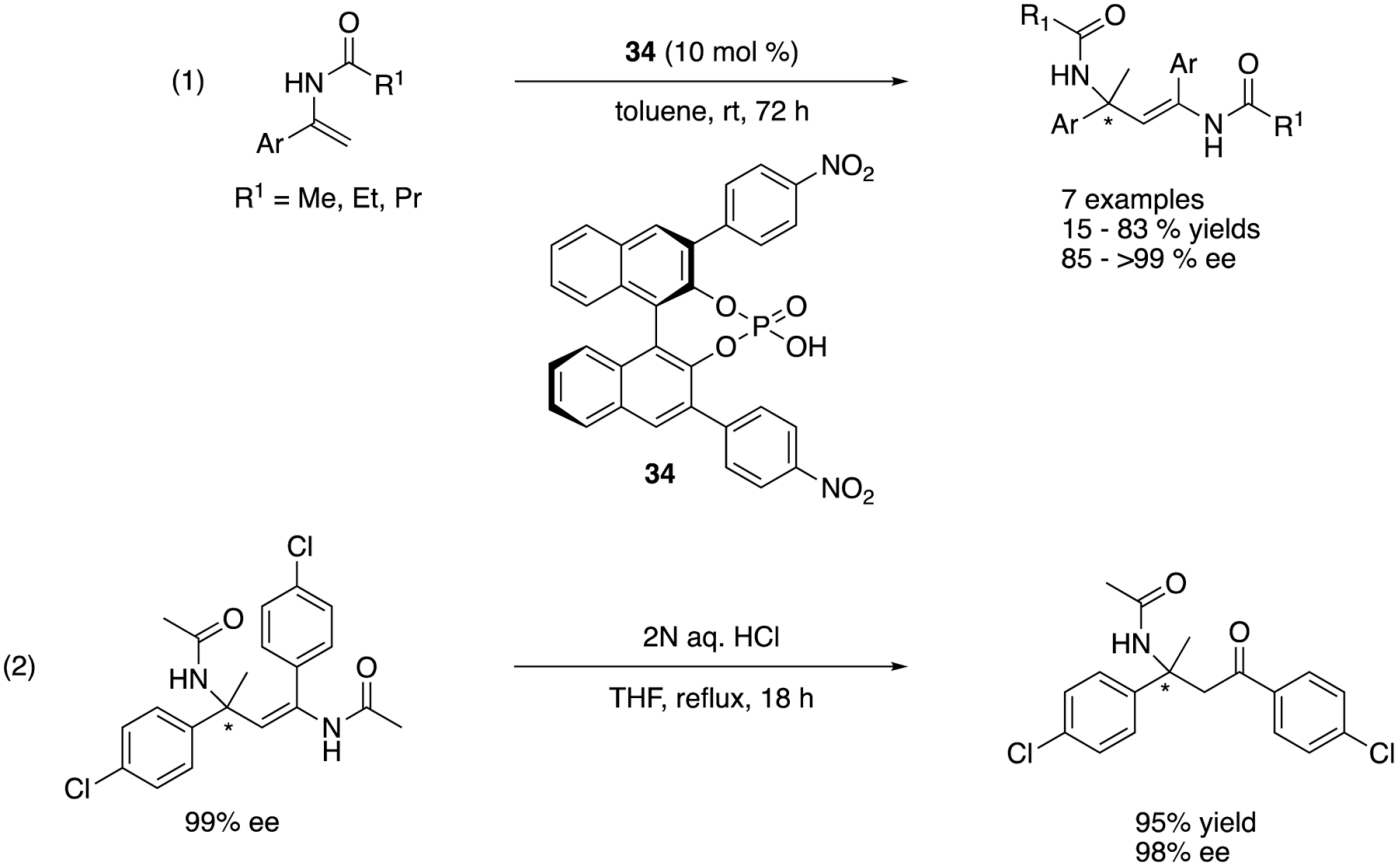
Asymmetric self-coupling of enamides by a BINOL-derived phosphoric acid.

In 2008, Tsogoeva and coworkers reported highly enantioselective self-coupling of enamides by using BINOL-derived phosphoric acids (Scheme 31) [108]. They took advantage of an equilibrium between ketimine and enamide in the presence of a chiral acid catalyst to utilize *N*-acyl ketimines that are otherwise difficult to employ as electrophiles due to the tautomerization. The method provided the self-coupled products with 15–83% yields with 85–>99% ee. They successfully demonstrated the utility of enantio-enriched self-coupled products by converting one example to a β-aminoketone without essential loss in its optical activity (2). This method is a viable alternative to the ketimine Mannich reaction.

### 3.4. Direct Mannich

**Scheme 32.**
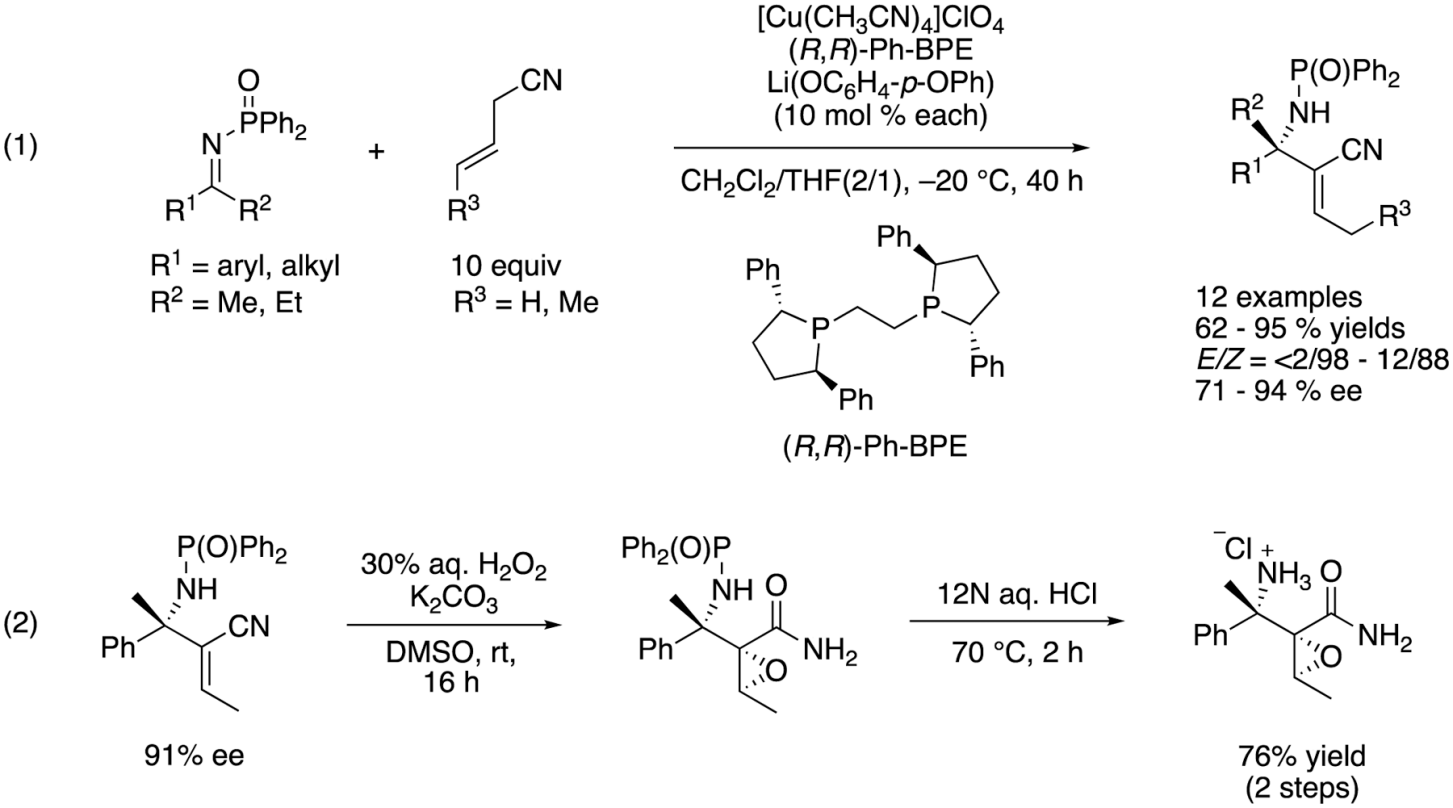
Direct catalytic asymmetric addition of allylic cyanide to ketimines.

In 2008/ Kumagai, Shibasaki, and coworkers reported a direct catalytic asymmetric addition of allylic cyanides to ketimines (Scheme 32) [109], They became interested in nitriles because these can be viewed as a masked carboxylic acid, are readily available, and could be small enough nucleophiles to overcome the severe steric demands of ketimines. Furthermore, they pointed out that allylic cyanides bear a relatively acidic α-proton (p*K*_a_ = 21.1 in DMSO), and thus, they could be selectively deprotonated by a base catalyst in the presence of ketimines. They hypothesized that a soft Lewis acidic Cu-Ph-BPE complex selectively coordinated to a cyanide so as to allow a phenoxide to deprotonate it (i.e., soft enolization). To their delight, the reaction scope turned out broad, giving the products in 62–95% yields with <2/98–12/88 *E*/*Z* ratios and 71–94% ee. They successfully demonstrated the utility of the products by converting one example to a densely functionalized β′-amino α,β-epoxyamide (2).

**Scheme 33.**
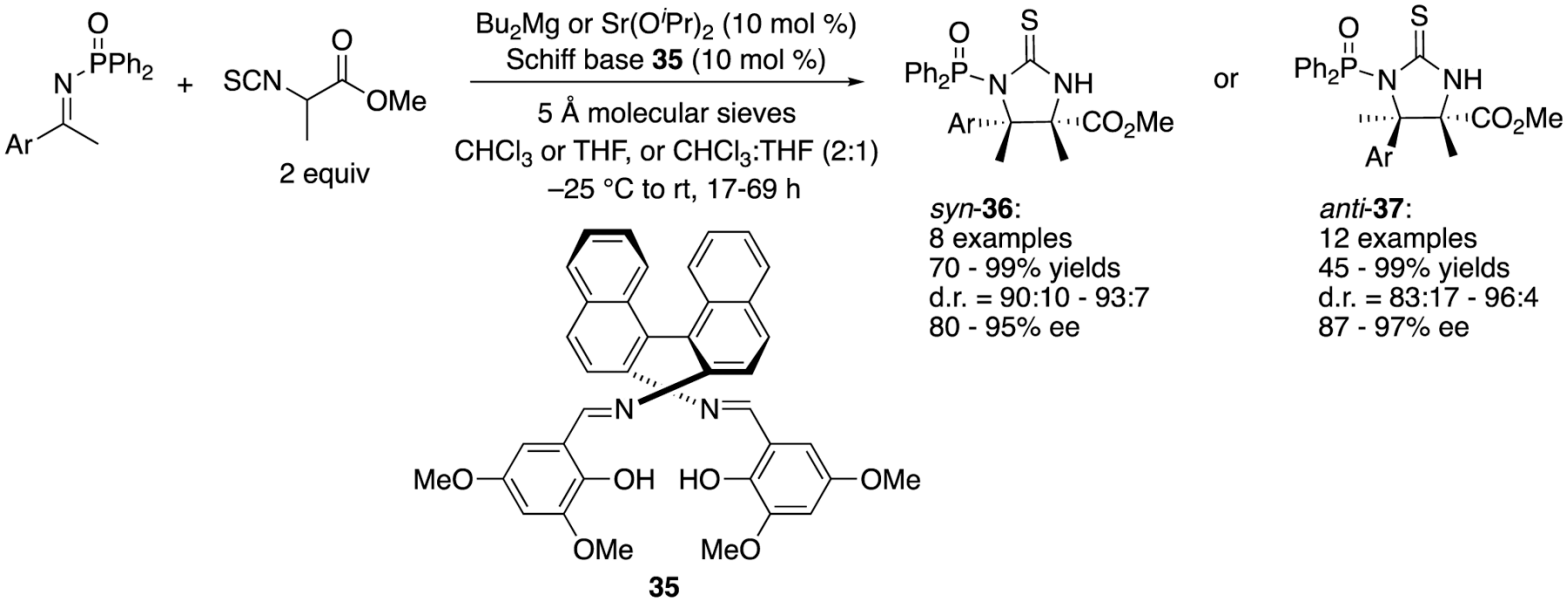
Stereodivergent direct catalytic asymmetric Mannich reaction.

As a direct access to α,β-diamino acid surrogates with vicinal tetrasubstituted stereocenters, Matsunaga, Shibasaki, and coworkers described the ketimine Mannich reaction with α-methyl-α-isothiocyanato ester in 2011 (Scheme 33) [110]. They began their study with catalysts made from Bu_2_Mg and BINAM-derived Schiff bases on the basis of their previous experiences with the aldol reaction of α-methyl-α-isothiocyanato ester and determined that the Schiff base shown in Scheme 33 was optimal with a model ketimine (Ar = 4-Br_6_C_6_H_6_). This chiral Mg catalyst afforded a syn diastereomer (**36**) as a major product (87% yield, d.r. = 91:9, 84% ee). In order to improve stereoselectivities, they further investigated Ca(O^i^Pr)_2_, Sr(O^i^Pr)_2_, and Ba(O^i^Pr)_2_ with the same Schiff base. To their surprise, Sr(O^i^Pr)_2_ led to an unexpected reversal of the diastereoselectivity, providing an anti-isomer (**37**) as a major product (Ar = 4-Br_6_C_6_H_6_, 86% yield, d.r. = 94:6, ee = 92%) while the other two metals gave only trace amounts of the products. The reversal of the diastereoselection resulted from the opposite ketimine facial selectivities by the Mg and Sr catalysts. The ^1^H NMR spectra of both catalysts were found to be complicated, which implicated a possibility of the oligomeric structures of theses catalysts. As such, the elucidation of the precise structures of the catalysts proved very challenging. Nonetheless, the Mg provided syn products in 70–99% yields with 90:10–93:7 diastereoselectivities and 80–95% ee, and the Sr catalyst gave anti products in 45–99% yields with 83:17–96:4 diastereoselectivities and 87–97% ee. To demonstrate the synthetic utility of the products, an anti product (Ar = 4-MeC_6_H_4_) was converted to a corresponding imidazoline and 2-phenyl-imidazoline (not shown).

**Scheme 34.**
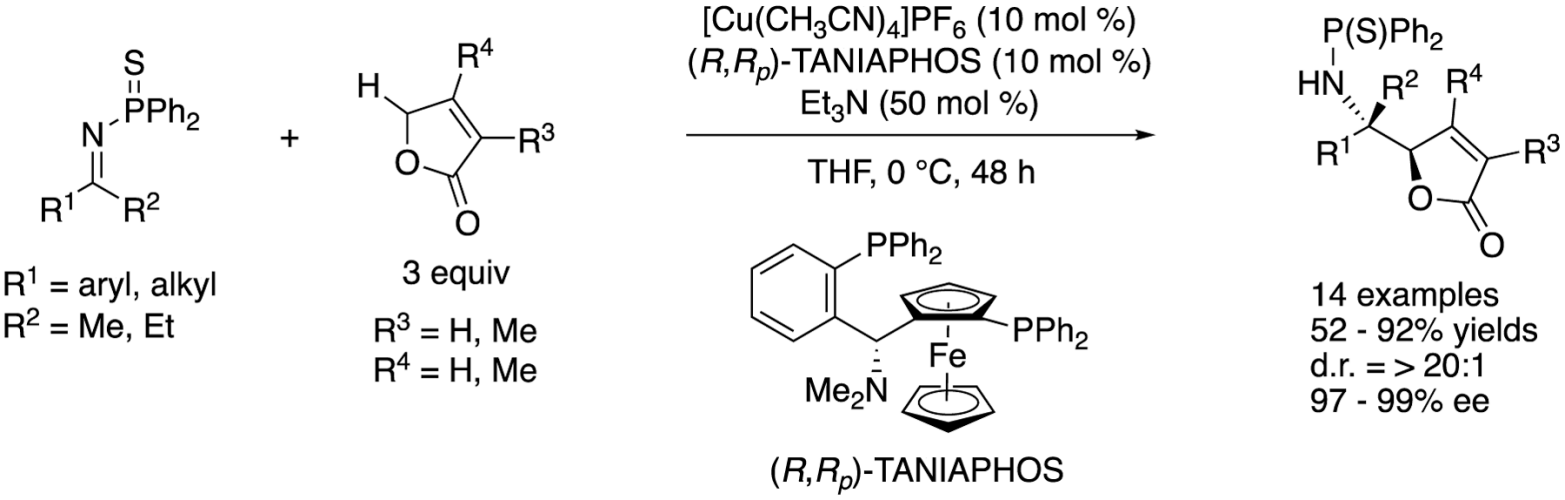
Direct catalytic asymmetric vinylogous ketimine Mannich reaction of γ-butenolides.

In 2013, Kumagai, Shibasaki, and coworkers reported a direct catalytic asymmetric vinylogous ketimine Mannich reaction of γ-butenolides (Scheme 34) [111]. For the development of direct catalytic asymmetric carbon-carbon bond-forming transformations under proton-transfer conditions, γ-butenolides have attracted much attention as useful pronucleophiles due to their relatively acidic protons that facilitate the generation of the corresponding dienolates and the high frequency of a γ-butenolide motif present in natural products and biologically relevant compounds. It is notable that prior to their contribution, γ-butenolides had not been employed in direct catalytic asymmetric vinylogous Mannich reactions with ketimines. On the basis of their continuing interests in the soft Lewis acid/hard Bronsted base cooperative catalysis (vide supra), they hypothesized that a soft Lewis acid would activate an *N*-(diphenylthiophosphinoyl)ketimine, while a hard Bronsted base would generate a dienolate from a γ-butenolide via deprotonation. They successfully demonstrated the importance of soft Lewis acid-soft Lewis base interaction (i.e., Cu⋯S interaction) by confirming that a *N*-phosphinoyl ketimine (R^1^ = Ph, R^2^ = Me) barely reacted under the optimized reaction conditions. The reaction scope was broad and included two alkyl methyl ketimines and two methyl substituted γ-butenolides, giving the products in 52–92% yields with >20:1 diastereoselectivity and 97–99% ee.

**Scheme 35.**
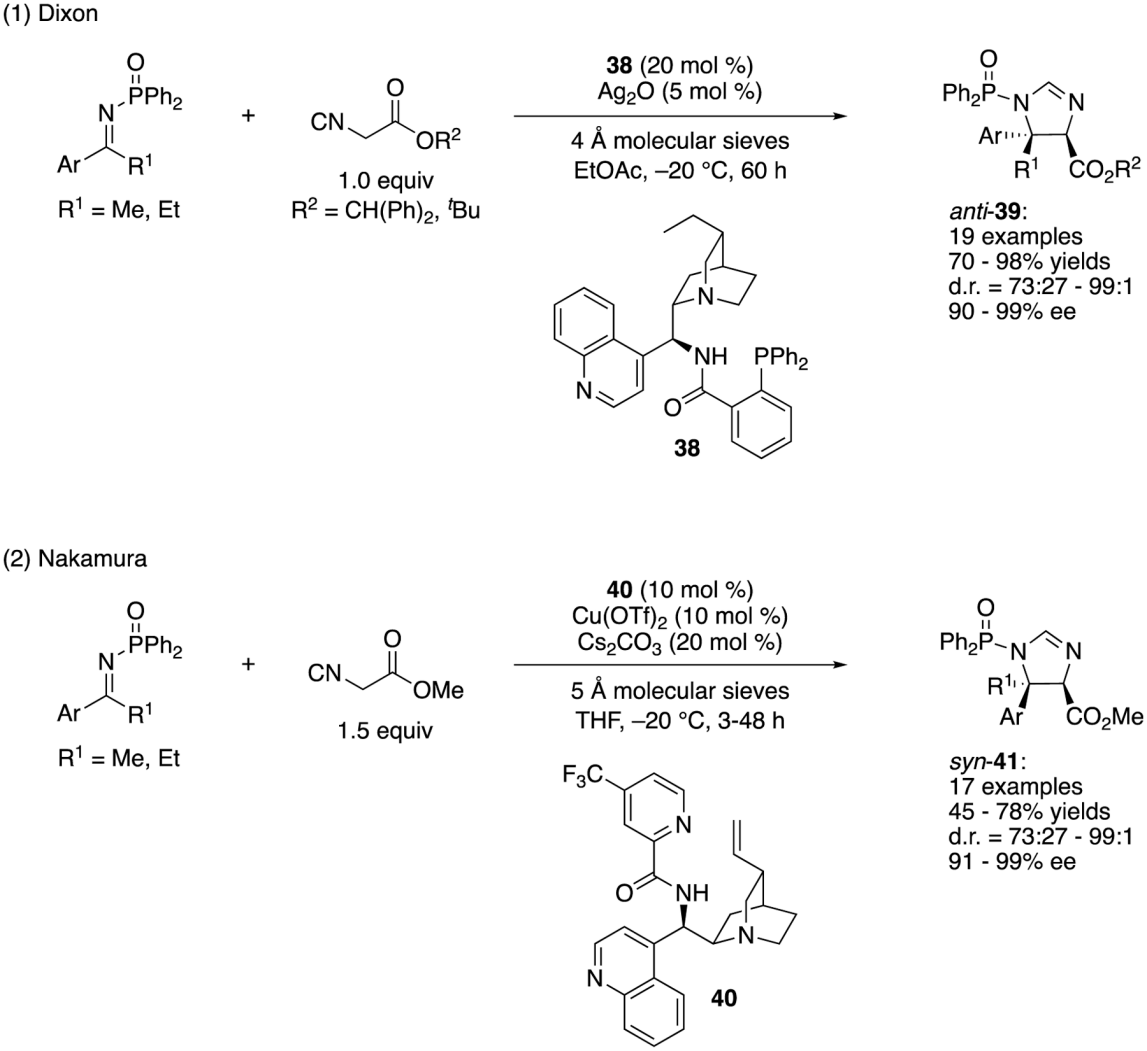
Direct asymmetric Mannich reactions of isocyanoacetates and ketimines.

In 2014, the groups of Dixon and Nakamura independently reported direct asymmetric Mannich reactions of isocyanoacetates and ketimines (Scheme 35) [112,113]. Despite the importance of α,β-diamino acid building blocks in chemical synthesis of biologically relevant molecules, catalytic enantioselective addition of isocyanoacetates to ketimines were not reported before their contributions.

Dixon and a coworker found that when a combination of cinchona-derived aminophosphine precatalyst **38** and silver oxide was employed as a binary catalyst system, anti-configured imidazoline products (**39**) were obtained in 70–98% yields with 73:27–99:1 diastereoselectivities and 90–99% ee (Scheme 35, (1)). It is notable that their method worked very well with the 1:1 ratio of ketimine and isocyanoacetate, indicating a high performance of their catalyst system. In general, excess of either electrophile or nucleophile is necessary for challenging asymmetric catalytic transformations such as the ketimine Mannich reaction. In 2016, they further developed this method to include α-substituted isocyanoacetates whose reaction scope was broad and included various aryl, heteroaryl, and alkyl methyl ketimines (not shown) [114]. Hydrolysis of these imidazoline products afforded access to fully substituted α,β-diamino acids in an enantio-enriched form.

Nakamura and coworkers reported that a complex made from alkaloid **40** and Cu(OTf)_2_ efficiently catalyzed the Mannich reaction of isocyanoacetate in the presence of CS_2_CO_3_ (Scheme 35, (2)). Their method provided complementary syn-configured imidazoline products (**41**) in 45–78% yields with 73:27–99:1 diastereoselectivities and 91–99% ee. The corresponding 1-indanone-derived ketimine (i.e., a cyclic substrate) was also a good substrate for their method, affording the product in 44% yield with 91:9 diastereoselectivity and 98% ee. Notably, this catalyst system was found to work for the aliphatic ketimines (not shown). Two alkyl methyl ketimines (Ar = PhCH_2_CH_2_ and ^*i*^Bu in (2)) gave anti diastereomers as major products (*anti:syn* = 72:28 and 60:40,54 and 78% yields, 72 and 89% ee, respectively). In 2016, they also disclosed their investigation of the ketimine Mannich reaction of α-substituted isocyanoacetates (not shown) [115]. A catalytic complex made from an alkaloid analogous to **40** and NiCl_2_ was found to be optimal for this reaction in which the substrate scope was broad. The enantioselective synthesis of imidazolines with vicinal tetrasubstituted stereocenters was achieved by this new chiral Ni complex.

**Scheme 36.**
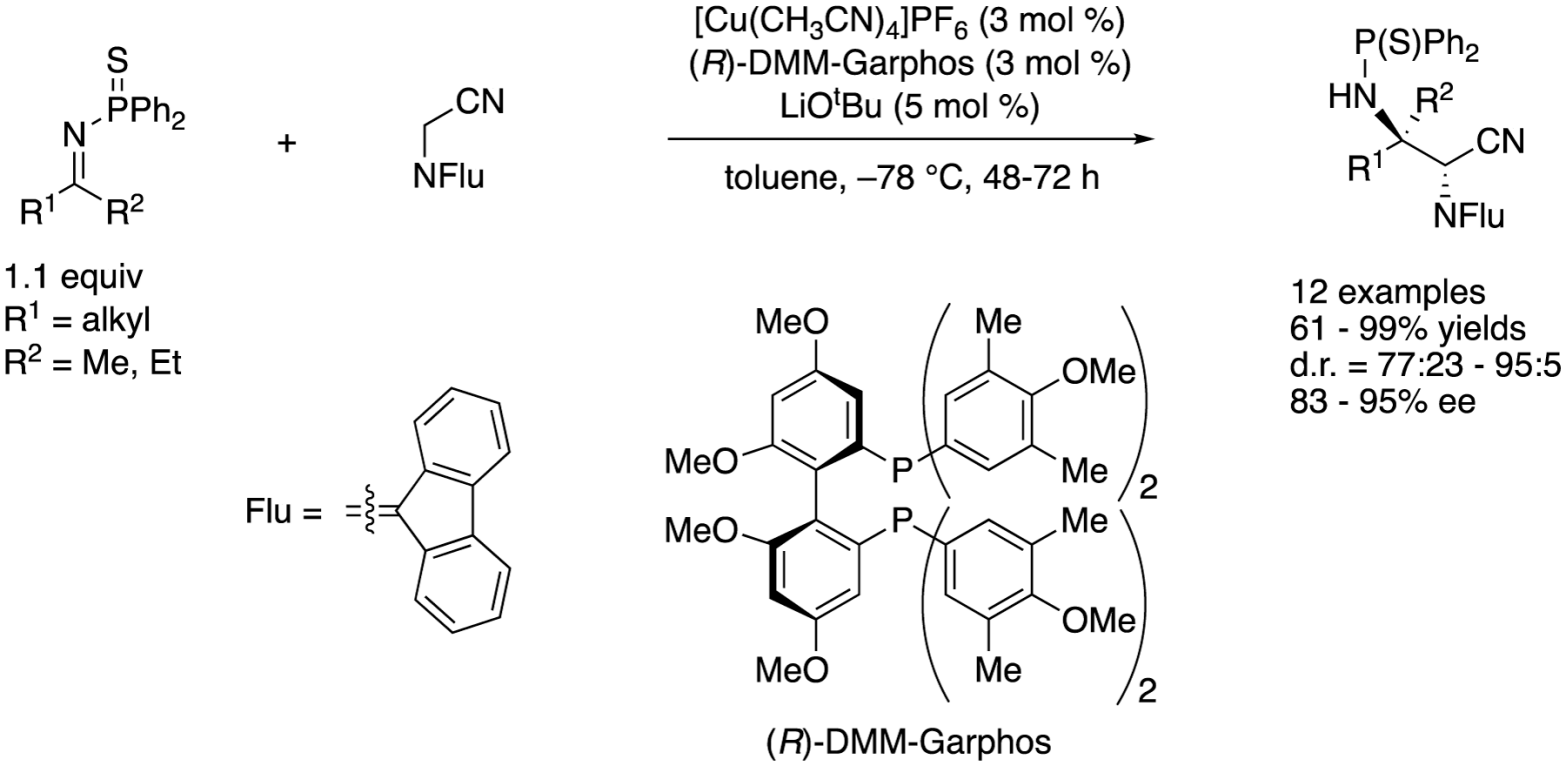
Direct catalytic asymmetric addition of *N*-alkylidene-α-aminoacetonitrile to aliphatic ketimines.

As part of their continuing interests in nitrile-based pronucleophiles and the soft Lewis acid/hard Brønsted base cooperative catalysis (vide supra), Kumagai, Shibasaki, and coworkers developed a direct catalytic asymmetric Mannich reaction of *N*-(9-fluorenylidene)-α-aminoacetonitrile and *N*-(diphenylthiophosphinoyl)ketimines (Scheme 36) [116]. The method provided vicinal diamines bearing tetra- and tri-substituted contiguous stereogenic centers in 61–99% yields with 77:23–95:5 diastereoselectivities and 83–95% ee. It is notable that their method was highly enantioselective for aliphatic ketimines where the structural difference of the two groups flanking a C=N bond was relatively small. Also worthy of mention is that only 3 mol% catalyst loading was enough, and it worked on a gram-scale reaction without any detrimental effect (R^1^ = CH_2_CH_2_Ph, R^2^ = Me, 48 h, 95% yield, d.r. = 91:9, 93% ee). Strangely enough, however, acetophenone-derived *N*-(diphenylthiophosphinoyl)ketimine provided only trace amounts of the product under the optimized reaction conditions. They conducted a preliminary mechanistic study and found that the analogous *N*-diphenylphosphinoyl ketimine (R^1^ = CH_2_CH_2_Ph, R^2^ = Me) resulted in much lower conversion and stereoselectivity under the reaction conditions, and this catalytic system exhibited higher performance for differentiating the prochiral face of ketimines rather than that of α-cyano carbanions. As such, the result suggested that the specific activation of the thiophosphinoyl group by a soft-Lewis acidic Cu(I) complex was crucial for the formation of a carbon-carbon bond and efficient stereochemical discrimination.

**Scheme 37.**
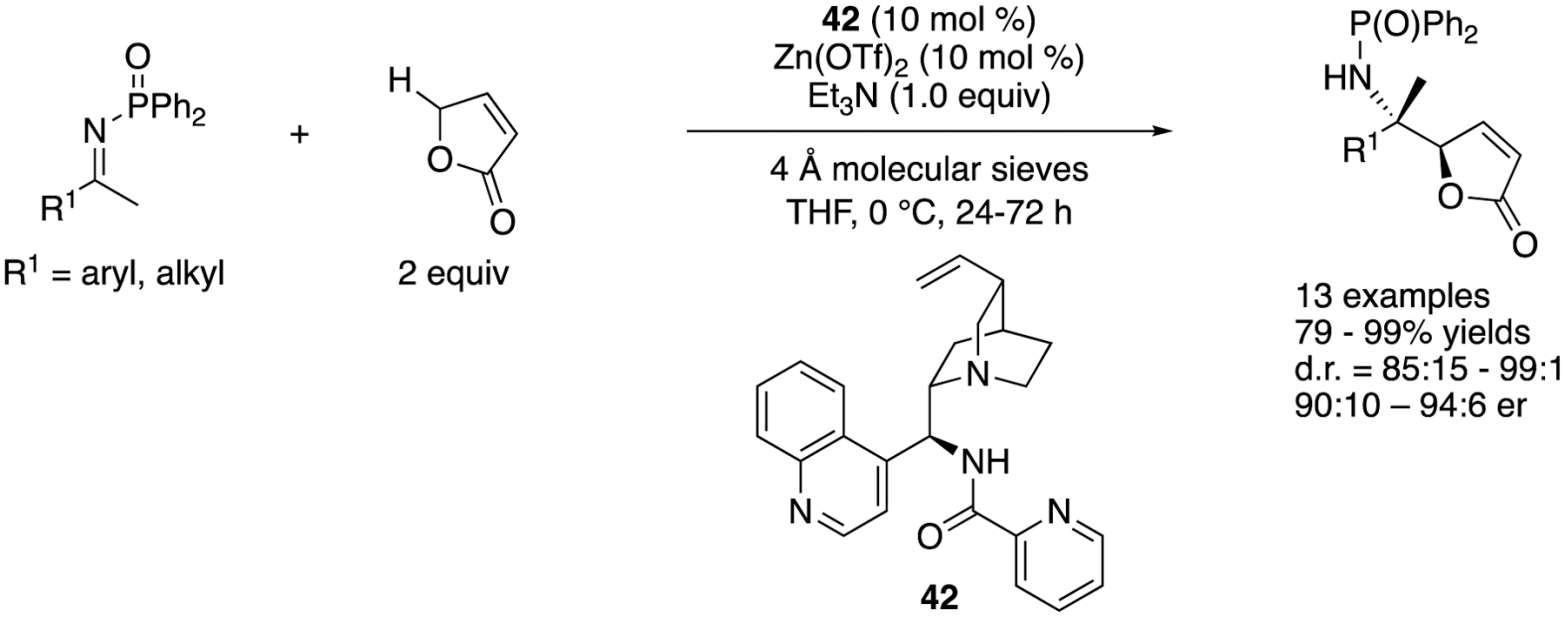
Direct enantioselective vinylogous Mannich reaction of ketimines with a γ-butenolide.

In 2015, Nakamura and coworkers reported that a complex made from alkaloid 42 and Zn(OTf)_2_ efficiently catalyzed the ketimine Mannich reaction of a γ-butenolide (Scheme 37) [117]. The ketimine scope was broad and included aryl, heteroaryl, and alkyl methyl ketimines as well as 1-indanone-derived ketimine. The products were obtained in 79–99% yields with 85:15–99:1 diastereoselectivities and 90:10–94:6 er. It is noteworthy that the pseudo-enantiomeric catalyst gave very similar reactivity and selectivity for three ketimines (R^1^ = 4-MeOC_6_H_4_, 4-FC_6_H_4_ and EtOCOCH_2_CH_2_, in 81–84% yields with 93:7–96:4 d.r. and 10:90–8:92 er) in this method, which is not always the case for cinchona-derived catalysts.

## 4. Activated and/or Cyclic Ketimines in the Allylation Reaction

The addition of allylic organometallic reagents to carbonyl and imine compounds represents an important process in the chemical synthesis (selected reviews; [118–121]). When ketimines are employed as electrophiles, chiral homoallylic α-tertiary amines are obtained. In light of a double bond in the products that can be viewed as a masked carbonyl, the allylation of the ketimines is analogous to the ketimine Mannich reaction.

### 4.1. Endocyclic Ketimines

#### 4.1.1. Allyl Rhodium Species

**Scheme 38.**
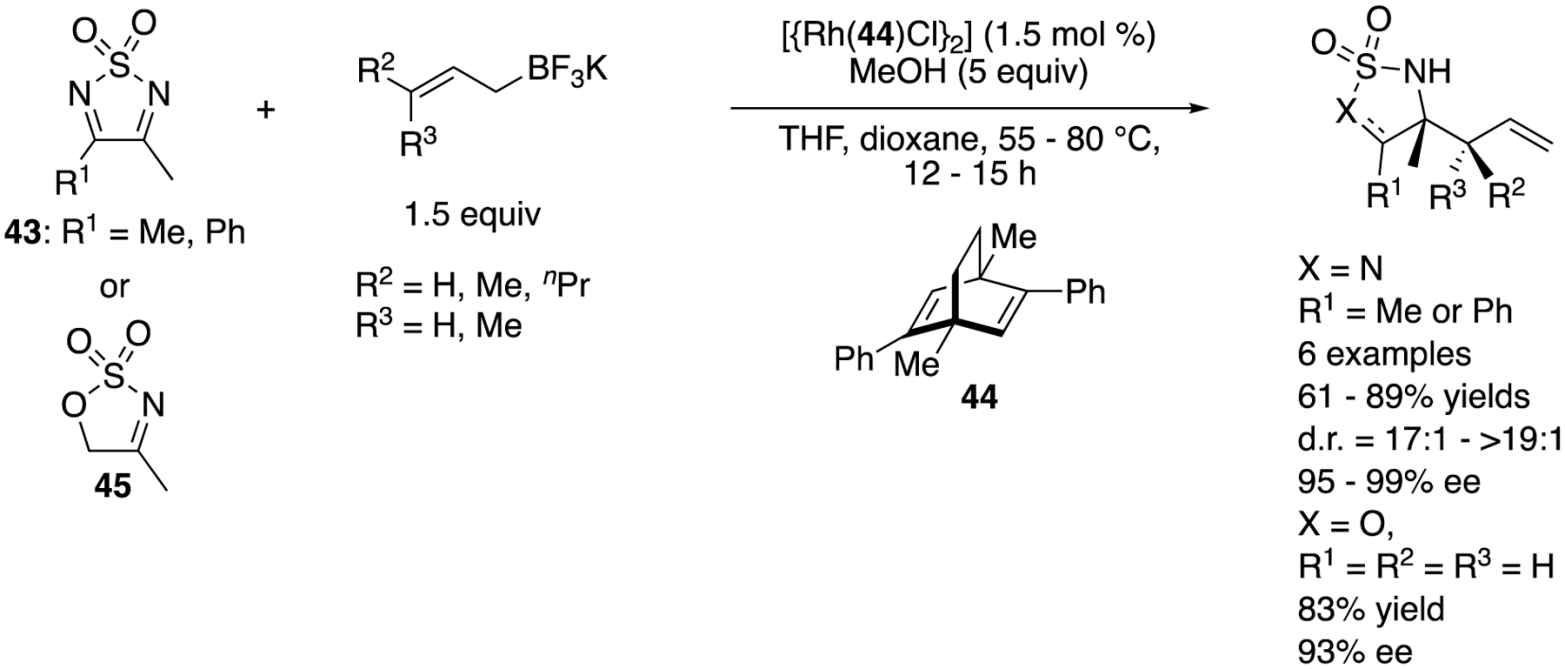
Enantioselective rhodium-catalyzed allylation of endocyclic ketimines.

In 2012, Lam and coworkers reported the first enantioselective rhodium-catalyzed addition of allylboron reagents to endocyclic ketimines (Scheme 38) [122]. Using only 1.5 mol% of the catalyst complex, 1,2,5-thiadiazolidine-l,1-dioxides (**43**) underwent the reaction with various allyl-, crotyl- and prenyl-trifluoroborates, affording the corresponding products in 61–89% yields with 17:1–>19:1 diastereoselectivities and 95–99% ee, as did a cyclic sulfamidate imine (**45**) with allyltrifluoroborate, which provided a corresponding homoallylamine in 83% yield with 93% ee. In subsequent years, they expanded the substrate scope of the method, which included endocyclic *N*-sulfonyl ketimines bearing CF_3_ and *n*-butyl groups at the imine carbon atom as well as various potassium allyltrifluoroborates (not shown) [123,124].

#### 4.1.2. Allyl Cobalt Species

**Scheme 39.**
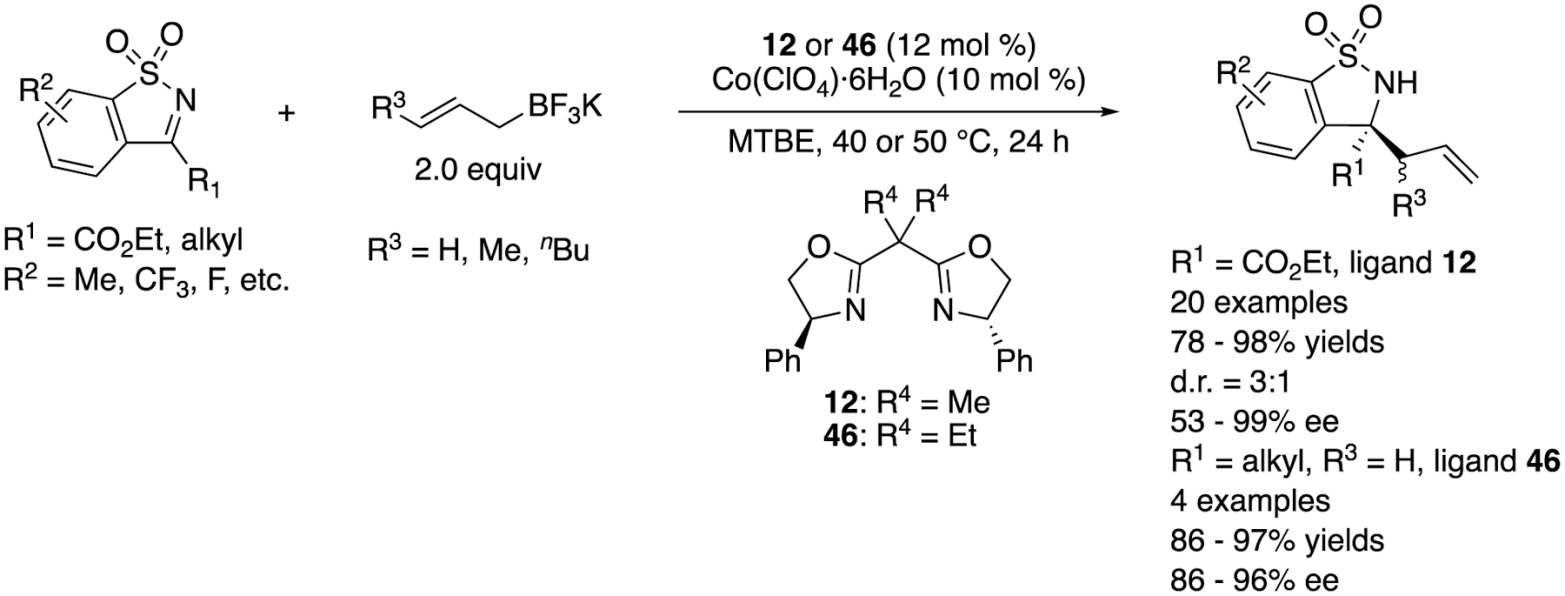
Enantioselective cobalt-catalyzed allylation of endocyclic ketimines.

In 2018, Yang, Zhang, and coworkers described that the same classes of electrophiles and nucleophiles that were previously studied with chiral rhodium complexes by Lam and coworkers (vide supra) underwent the allylation reaction catalyzed by chiral complexes generated from more cost-effective Co(ClO_4_)·6H_2_O and BOX ligands. The method afforded the enantio-enriched homoallylamines in 78–98% yields with 3:1 diastereoselectivity and 53–99% ee (Scheme 39) [125].

### 4.2. Isatin-Derived and Analogous Cydic Ketimines

#### 4.2.1. Allyl Palladium Species

**Scheme 40.**
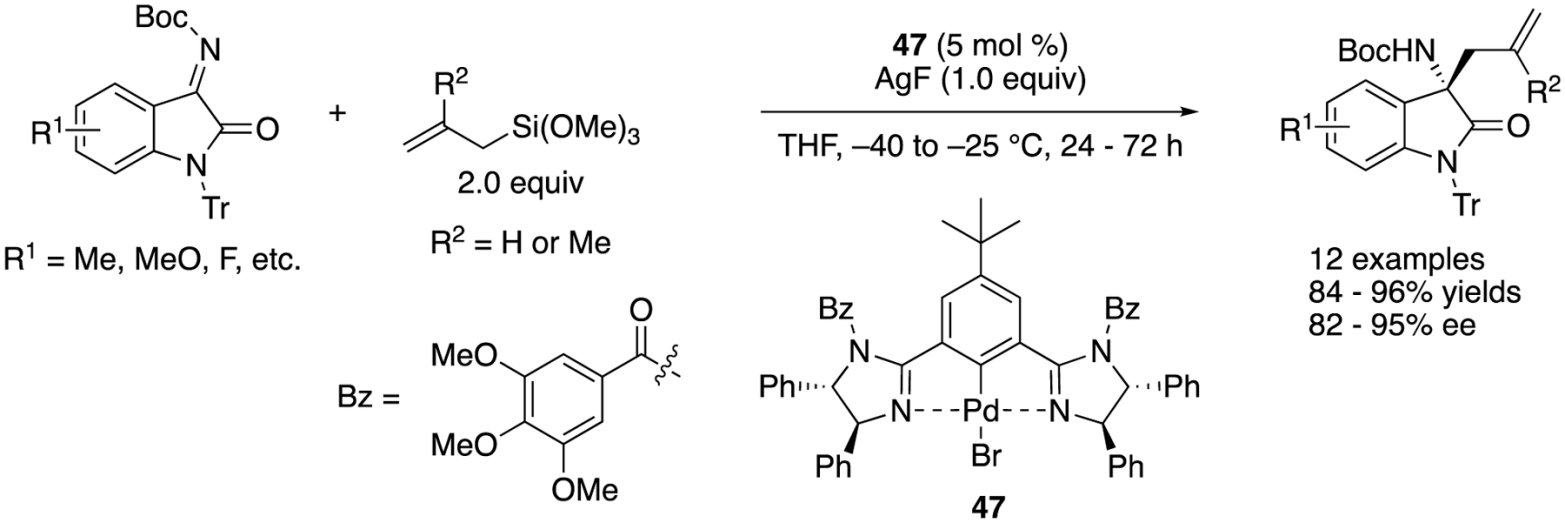
Enantioselective allylation of isatin-derived ketimines catalyzed by a chiral Pd-pincer complex.

Ketimines derived from isatins have attracted much attention as electrophiles because they provide chiral 3-substituted 3-amino-2-oxindoles that are a structural motif found in biologically relevant compounds and natural products, and they are relatively reactive electrophiles, as discussed above. However, asymmetric catalytic allylation of isatin-derived ketimines had not been reported prior to a contribution made by Nakamura and coworkers (Scheme 40) [126]. Chiral allyl palladium species generated from bis(imidazoline)-palladium pincer catalyst **47** and allyltrimethoxysilanes through transmetalation underwent the allylation reaction with *N*-teri-butoxycarbonyl-*N*′-trityl-protected isatin-derived ketimines. Under the optimized reaction conditions, allylation of ketimines with both electron-donating and -withdrawing substituents provided the corresponding products in 84–96% yields with 82–95% ee.

#### 4.2.2. Allyl Bismuth Species

**Scheme 41.**
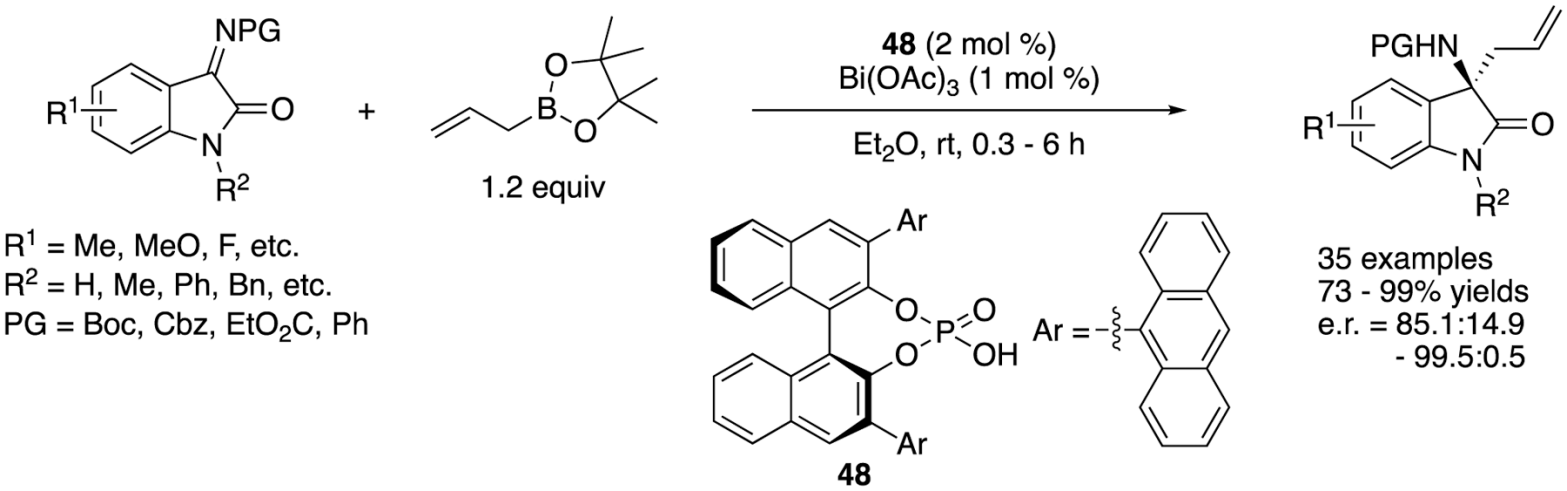
Bi(III)-catalyzed enantioselective allylation of ketimines.

In 2019, Li and coworkers described the development of an enantioselective asymmetric allylation of isatin-derived ketimines with allylboronates promoted by a binary acid system containing bismuth acetate and chiral phosphoric acid (Scheme 41) [127]. It is notable that most of the ketimines investigated were allylated in less than an hour at room temperature with only 1 mol% of Bi(OAc)_3_ and 2 mol% of chiral phosphoric acid **48**. The method gave chiral homoallylic α-tertiary amines in 73–99% yields with 85.1:14.9–99.3:0.7 enantiomeric ratios. An endocyclic *N*-sulfonyl ketiminoester and a pyrazoledione-derived ketimine were included in their study (not shown). While the former was comparable to the isatin-derived ketimines, the latter took 40 h to provide the corresponding product in 98% yield with 85.3:14.7 enantiomeric ratio. Regarding the actual nucleophile, they proposed that a chiral allyl bismuth complex generated from allylboronate and two molecules of chiral phosphoric acids through transmetalation would be the allylation species, because the α-selectivity was observed with 1-methylalylbornoic acid pinacol ester, and a positive nonlinear effect between the ees of chiral phosphoric acids and products was observed.

#### 4.2.3. Allenyl Copper Species

**Scheme 42.**
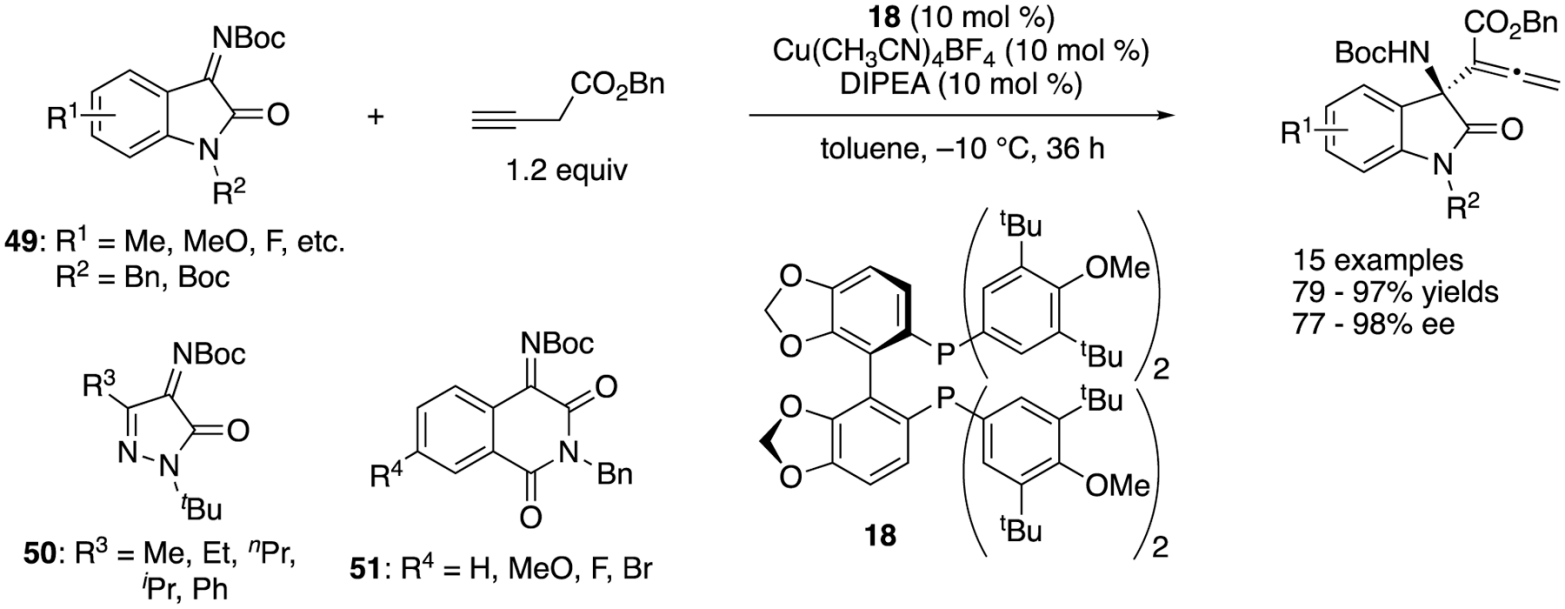
Cu(I)-catalyzed asymmetric α-allenylation of activated ketimines.

In 2020, Du, Chen, and coworkers reported Cu(I)-catalyzed asymmetric α-allenylation of activated ketimines with 3-butynoates (Scheme 42) [128]. Screening of catalysts and reaction conditions was conducted with isatin-derived ketimines. With an optimal catalytic system, α-allenylation of fifteen isatin-derived (**49**), five pyrazoledione-derived (**50**), four isoquinoline-1,2,3-trion-derived (**51**) ketimines, and one trifluoromethyl alkynyl ketimine (not shown) were evaluated. The first two classes of ketimines underwent the reaction at −10 °C and provided the corresponding products after 36 h in 79–97% yields with 77–98% ee, and 64–91% yields with 84–92% ee, respectively In contrast, isoquinoline-1,2,3-trion-derived ketimines took 72 h at rt to provide the products in 59–63% yields with 73–92% ee. With respect to the actual nucleophile, it was suggested that 3-butynoate generated the corresponding copper enolate intermediate in the presence of DIPEA, which isomerized to γ-allenyl copper species.

#### 4.2.4. Allyl Gold Species

**Scheme 43.**
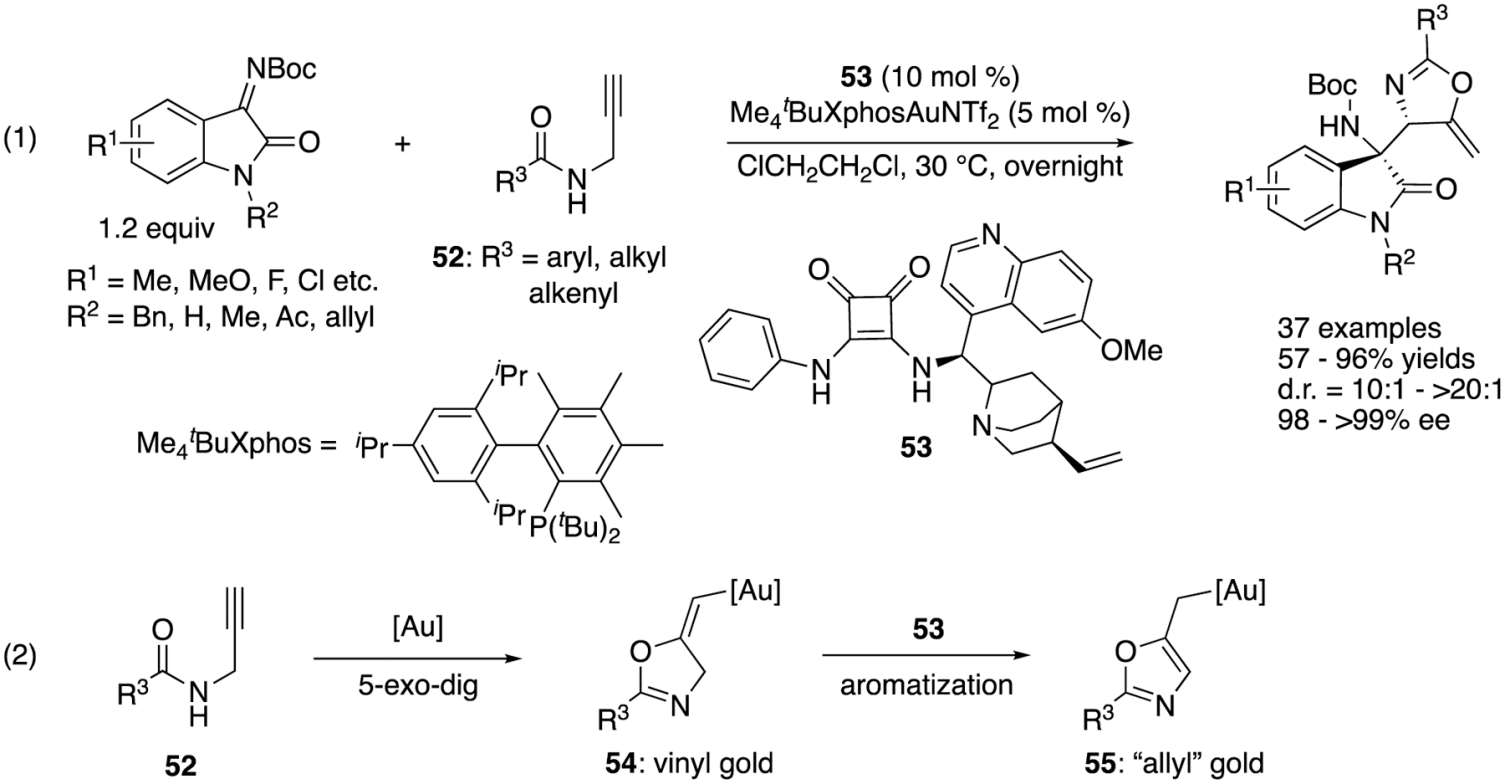
Asymmetric allylation of ketimines by allyl gold intermediates promoted by squaramide.

In 2021, Hu, Xu, and coworkers developed a gold and chiral organocatalyst cooperative catalysis strategy for the allylation of isatin-derived ketimines with readily available *N*-propargylamides **52** (Scheme 43) [129], Their method tolerated various substituents on both substrates and provided the products in 57–96% yields with 10:1–>20:1 diastereoselectivities and 98–>99% ee. As they obtained an X-ray crystal structure of the allyl gold intermediate (**55**, R^3^ = Ph), they hypothesized that allyl gold species resulted through the aromatization of the corresponding vinyl gold species promoted by a basic functionality of squaramide **53**. On this basis, they proposed that the squaramide electrophilically activated a ketimine via dual-hydrogen bonding, while its nitrogen atom coordinated with the gold catalyst, leading to a formal intramolecular reaction (i.e., bifunctional catalysis by the squaramide).

### 4.3. Ketimines Bearing a CF_3_ and/or Carbonyl Moiety etc

#### 4.3.1. Allyl Indium Species

**Scheme 44.**
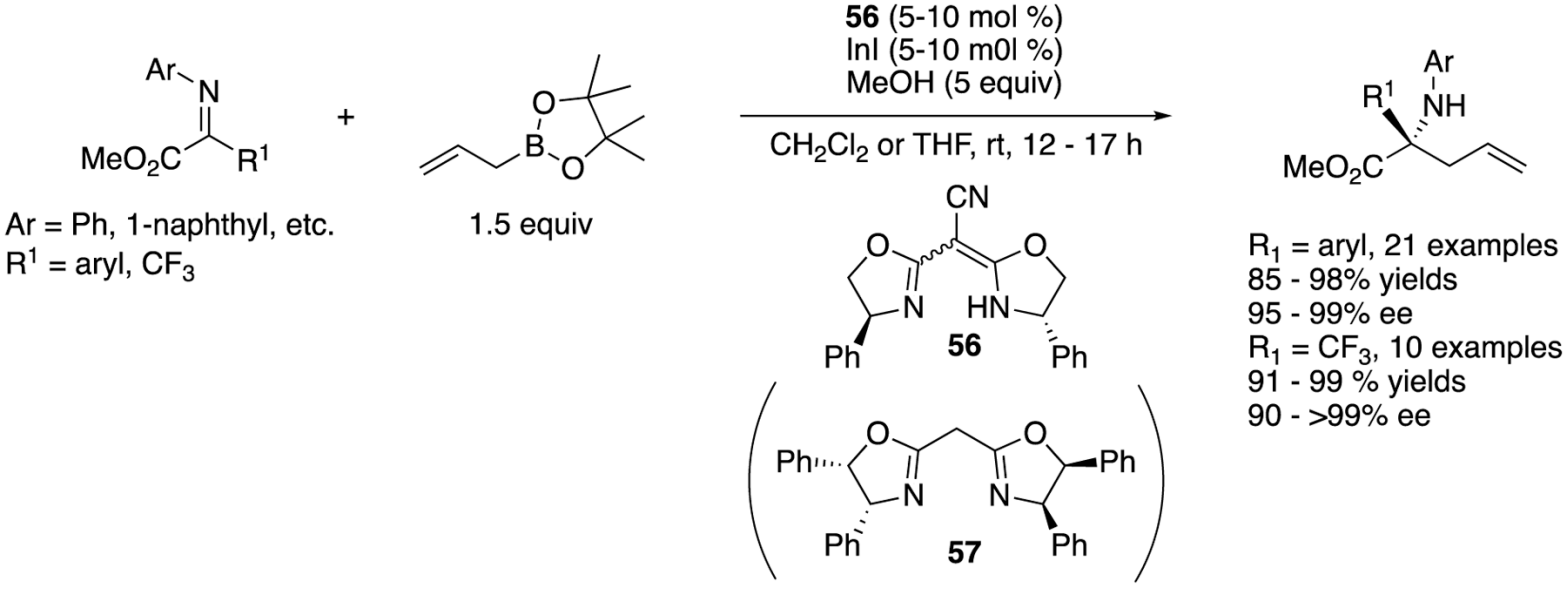
Enantioselective catalytic allylation of acyclic ketiminoesters.

In 2019, Kürti and coworkers described the first direct catalytic enantioselective allylation of acyclic keti mi noesters to provide α-allyl-α-aryl and α-allyl-α-trifluoromethyl amino esters (Scheme 44) [130]. They identified that a complex generated from a commercially available BOX-type ligand (**56**) and Ini as an optimal catalyst for both α-aryl- and α-trifluoromethyl-α-ketiminoesters. Their method afforded α-allyl-α-aryl and α-allyl-α-trifluoromethyl amino esters in 85–98% yields and 95–99% ee with 5 mol% catalyst loading in CH_2_CI_2_, and 91–99% yields and 90–99% ee with 10 mol% catalyst loading in THF, respectively. These reactions were scalable to a gram-scale with no deterioration of the yield or enantiopurity. Since an enantiomer of the optimal ligand (**56**) is not commercially available and would require several steps to synthesize, they found another commercially available ligand **57** that performed comparably to ligand **56** with the opposite sense of enantioselection, obtaining the corresponding enantiomers in six α-aryl- and three α-trifluoromethyl-α-ketiminoesters examined.

#### 4.3.2. Allyl Boronate Species

**Scheme 45.**
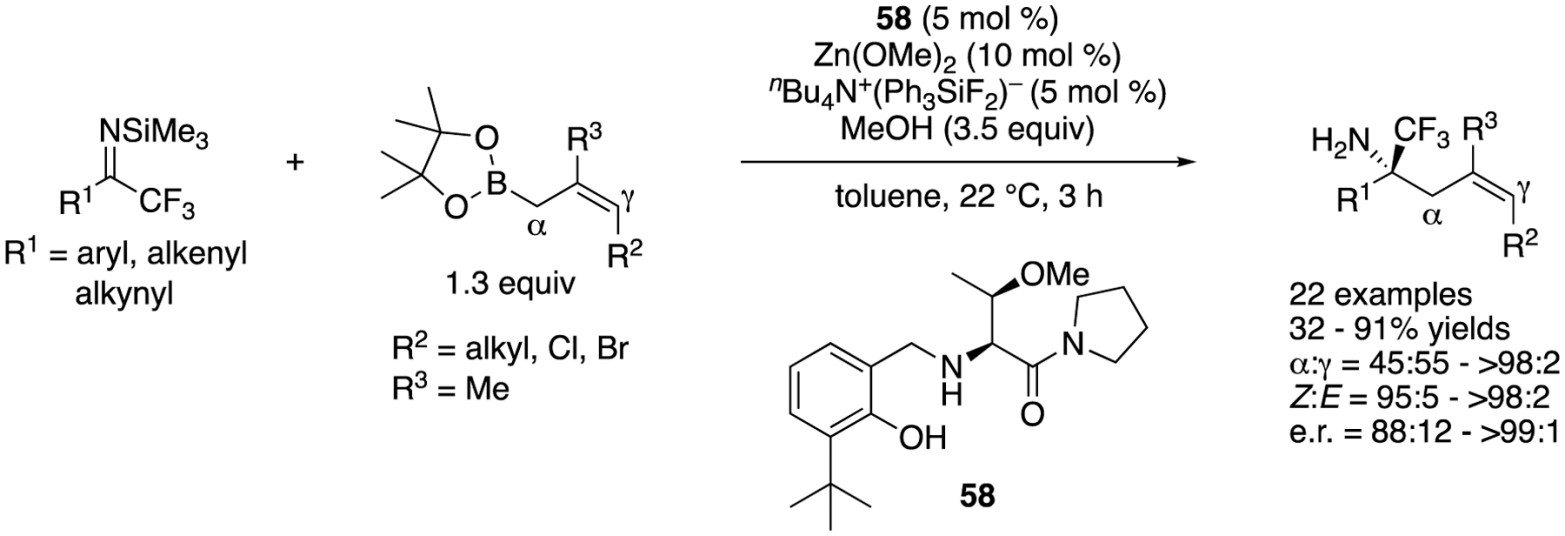
Regio- and enantio-selective allylation of N–H ketimines generated in situ.

In 2020, Hoveyda and coworkers reported a catalytic regio- and enantio-selective synthesis of trifluoromethyl-substituted homoallylic α-tertiary primary amines (Scheme 45) [131]. The reaction scope was very broad and included aryl-, heteroaryl-, alkenyl-, and alkynyl- trifluoromethyl ketimines, and γ-substituted Z-allyl and β,γ-trisubstituted Z-allyl boronates. Unprotected N–H ketimines generated in situ from corresponding *N*-silyl ketimines were allylated by an *O*-methyl-l-threonine-derived aminophenol-based boryl catalyst, giving the desired products in 32–91% yields with 45:55–>98:2 α:γ selectivity, 95:5–>98:2 *Z:E* selectivity, and 88:12–>99:1 enantiomeric ratios. It is worth mentioning that (1) *N*-trimethylsilyl aryl-trifluoromethyl ketimines are stable and readily prepared in multigram quantities and (2) this allylation reaction was demonstrated on a gram-scale with no deterioration of the yield, regio- and stereo-selectivities.

#### 4.3.3. Allyl Copper Species

**Scheme 46.**
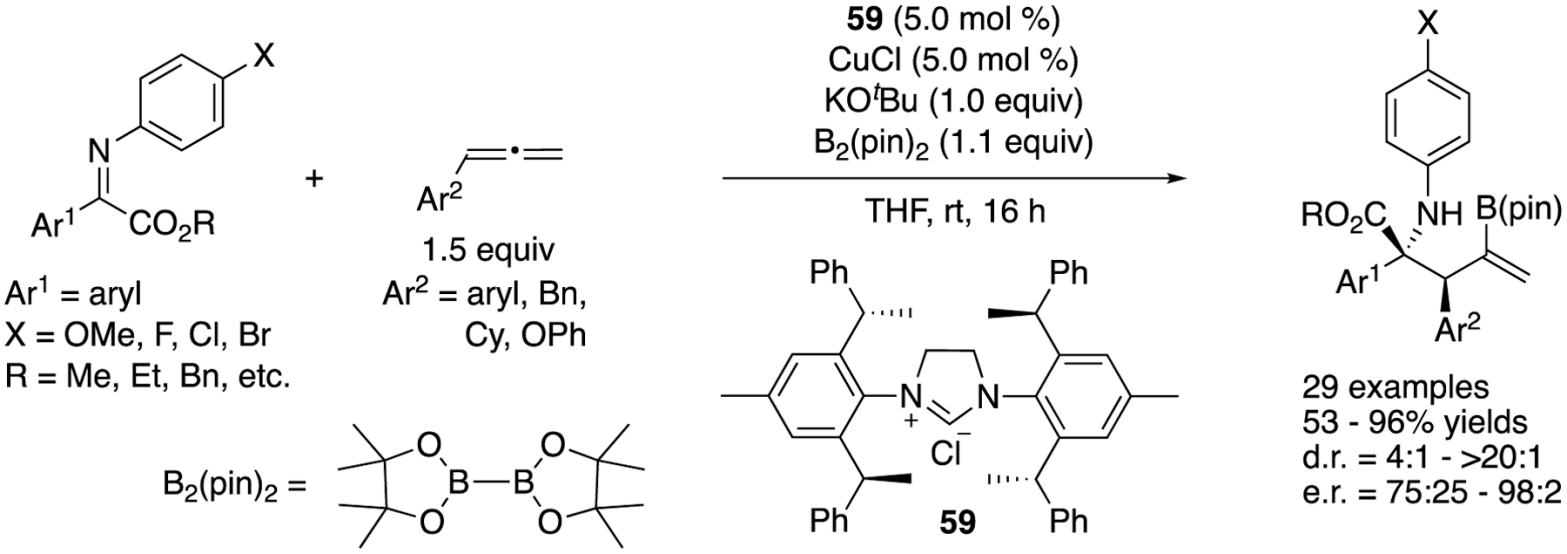
Enantioselective catalytic allylation of acyclic ketiminoesters via carboboronation of allenes.

In 2020, Chen and coworkers described an asymmetric allylation of acyclic ketiminoesters through copper-catalyzed carboboronation of allenes (Scheme 46) [132]. They found that the use of a bulky *C*_2_-symmetric NHC was the key to control the chemo-, regio-, diastereo-, and enantio-selectivities in their protocol. While the scope of the electrophiles was limited to alkyl aryl glyoxylate-derived ketiminoesters (Ar^1^ = aryl), that of allenes was found broad, affording the products in 53–96% yields with 4:1–>20:1 diastereoselectivities and 75:25–98:2 enantiomeric ratios.

## 5. Unmodified Ketimines in the Allylation Reaction

### 5.1. AUyl Copper Species

**Scheme 47.**
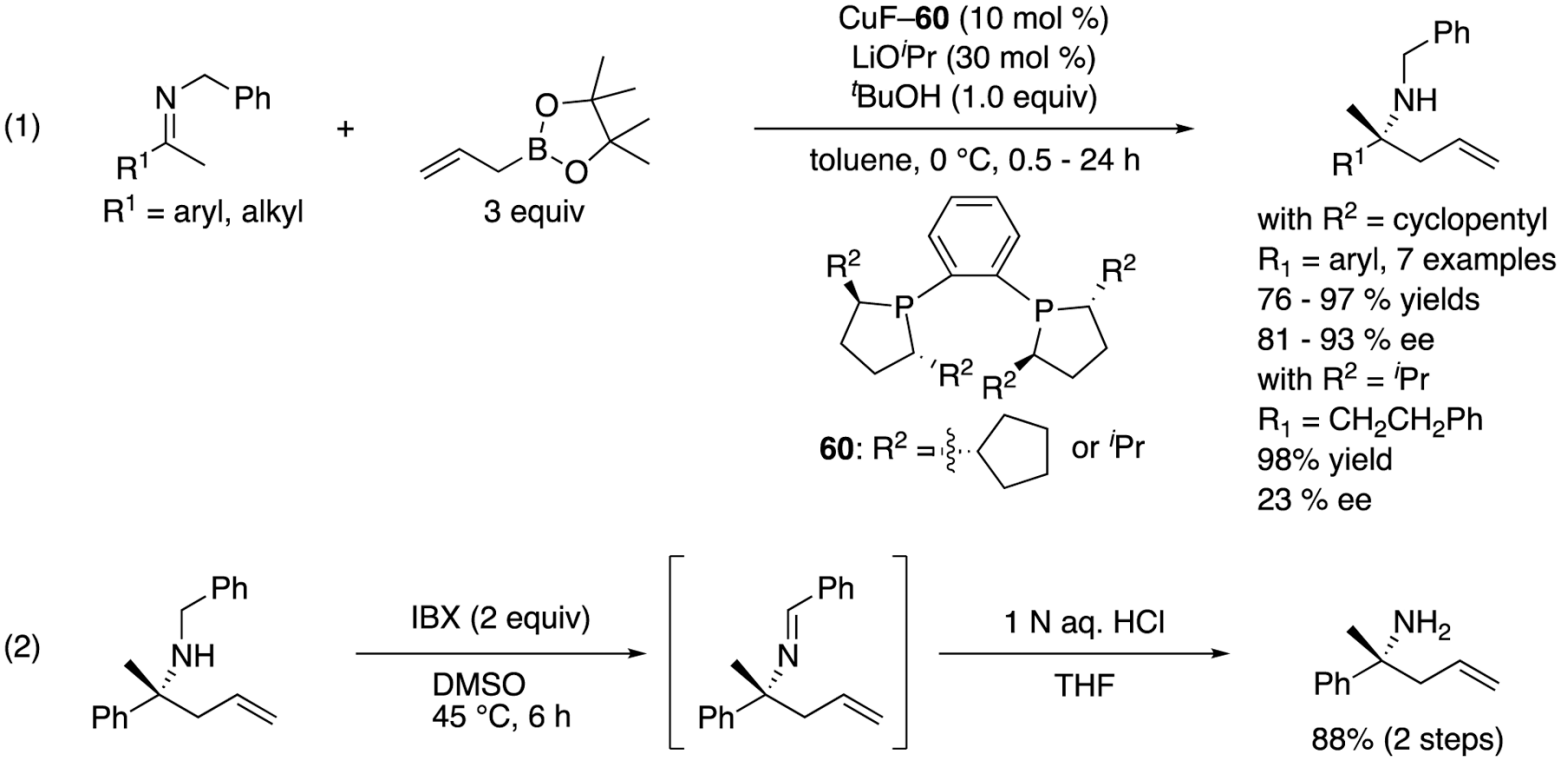
The first catalytic enantioselective ketimine allylation reaction.

In 2006, Kanai, Shibasaki, and coworkers reported the first catalytic enantioselective ketimine allylation reaction (Scheme 47, (1)) [133]. On the basis of their previous studies on the Cu-catalyzed allylboration of ketones, they hypothesized that a highly nucleophilic allylcopper species generated from allylboronate would also be a suitable nucleophile for ketimines. They first investigated the effect of different ketimine *N*-protecting groups on the reactivity by using achiral CuF·3PPh_3_ as a catalyst and selected *N*-benzylketimines for the development of an enantioselective variant. Cyclopentyl-DuPHOS was identified as an optimal chiral ligand for aryl methyl ketimines, providing homoallylic amines in 76–97% yields with 81–93% ee. On the other hand, ^*i*^Pr-DuPHOS was better for an aliphatic ketimine, giving a corresponding homoallylamine in 98% yield with 23% ee. The removal of *N*-benzyl group was done in two steps with an overall yield of 88% (2).

**Scheme 48.**
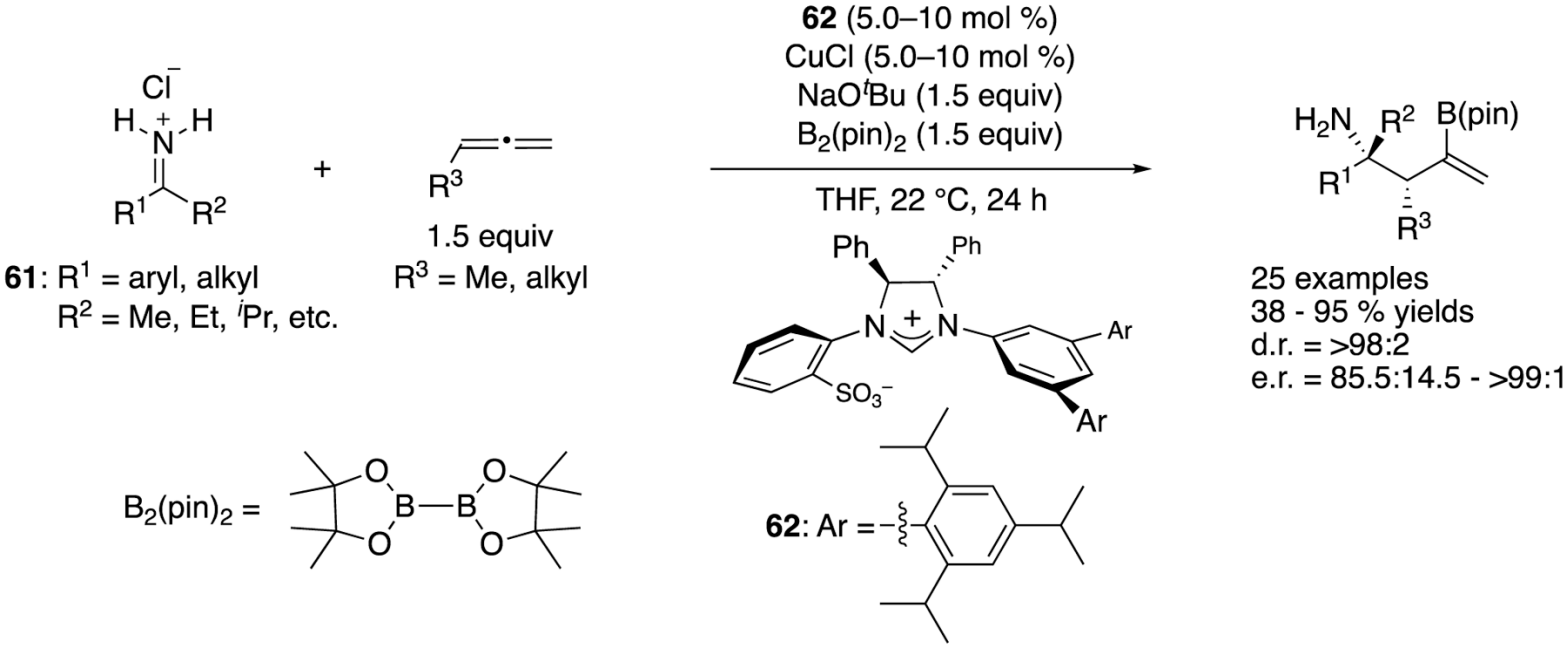
State-of-the-art catalytic enantioselective allylation of unmodified ketimines.

In 2017, Hoveyda and coworkers disclosed the first asymmetric catalytic allylation of unprotected N-H ketimines generated in situ from the corresponding salts (**61**), which directly provided enantio-enriched α-tertiary primary amines (Scheme 48) [134], This class of ketimine salts is bench-stable and prepared by the addition of organo-lithium or magnesium species to readily available nitriles followed by HC1 treatment [32]. This method was not only atom and step economical but also obviated possible complications associated with the removal of *N*-protecting units in the products. With respect to the actual nucleophiles, the chiral NHC-Cu complex produced corresponding chiral (Z)-allyl copper intermediates through borylcupration of monosubstituted allenes with B_2_(pin)_2_, which underwent the addition reaction to N–H ketimines. The reaction scope was very broad in terms of both ketimines and allenes, and the primary amine products were obtained in 38–95% yields with 85.5:14.5–>99:1 enantiomeric ratios and exceptional diastereoselectivity (>98:2 throughout). They noted that reactions of ketimines that contained an α- or (β-alkoxy or a benzyl group were inefficient, probably due to facile decomposition (enamine formation and β-elimination, respectively) and that the same applied to trifluoromethyl-substituted ketimines (decomposition to unidentified products). This catalytic method puts forward an expeditious strategy for the synthesis of α-tertiary homoallylamines (β-tertiary-amino carbonyl equivalents) with a very broad substrate scope in high diastereo- and enantio-selectivities, thus providing a solution to an important and persisting problem in catalytic enantioselective synthesis. Indeed, this method is the state of the art in the ketimine allylation.

**Scheme 49.**
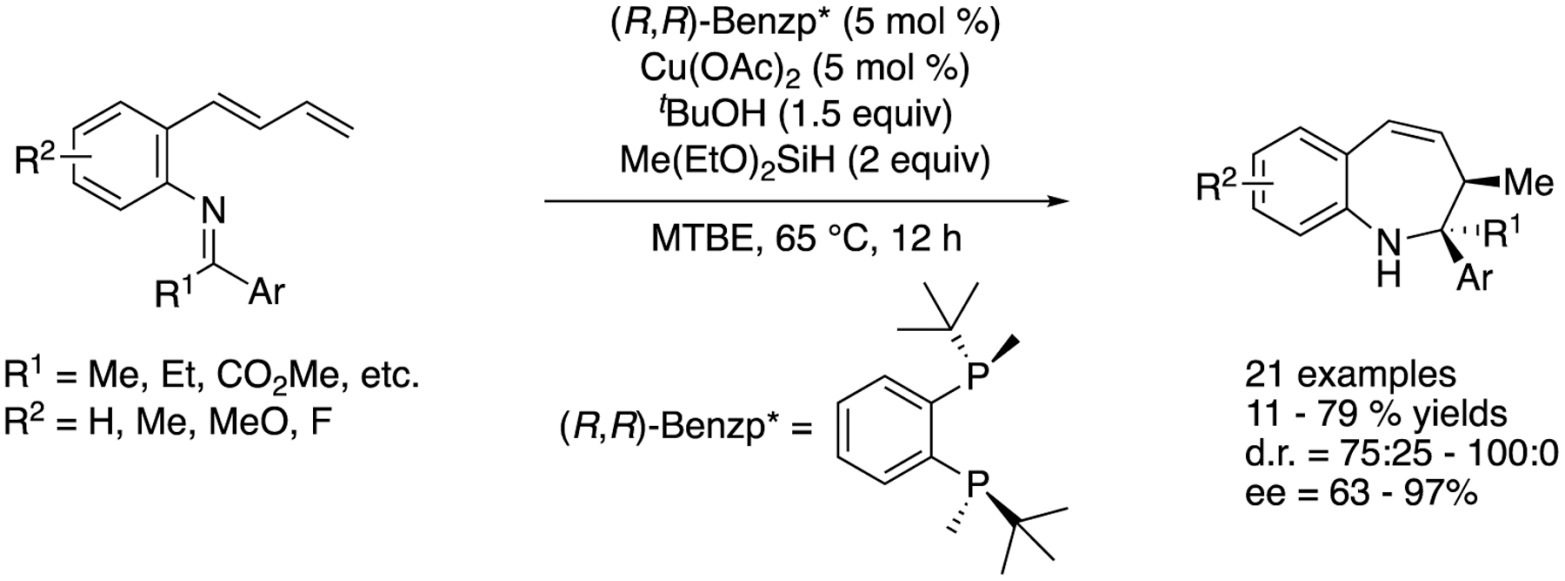
Catalytic asymmetric intramolecular allylation of ketimines.

In 2019, Yun and coworkers described copper-catalyzed asymmetric intramolecular reductive coupling of (*E*)-dienyl arenes with a tethered ketimine moiety (Scheme 49) [135]. This transformation was a sequence of a chemo-, regio-, and enantio-selective hydrocupration reaction of (*E*)-dienes that produced allyl copper species and their subsequent intramolecular addition to ketimines. The reaction scope was broad and provided enantio-enriched 1-benzazepine derivatives bearing two contiguous stereocenters in 11–79% yields with 75:25–100:0 diastereoselectivities and 63–97% ee.

### 5.2. Pd-Trimethylenemethane (TMM)

**Scheme 50.**
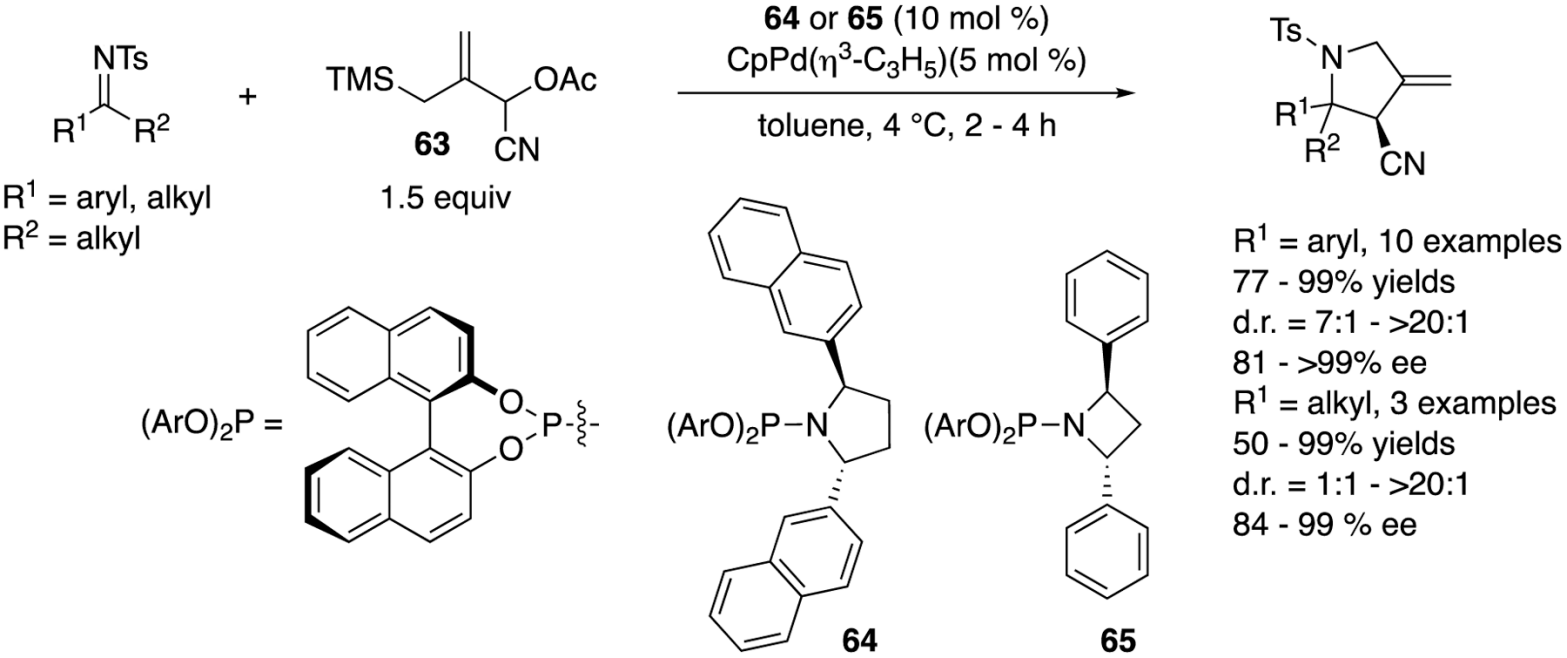
Pd-catalyzed asymmetric [3 + 2] cycloaddition of trimethylenemethane with ketimines.

In 2010, Trost and a coworker reported that a zwitterionic complex (Pd-TMM) generated in situ from a Pd catalyst and l-cyano-2-((trimethylsilyl)methyl)allyl acetate (**63**) underwent facile cycloaddition reaction with various ketimines (Scheme 50) [136,137]. The electrophile scope was very broad and included aryl alkyl, cyclic, and dialkyl ketimines. Phosphoramidite ligand **64** was found to be optimal for aryl alkyl and cyclic ketimines and provided the corresponding products in 77–99% yields with 7:1–>20:1 diastereoselectivities and 81–>99% ee but gave an unacceptable level of diastereoselectivity for cyclohexyl methyl ketimine. Ligand **65**, however, turned out fruitful for dialkyl ketimines, affording the cycloadducts in 50–99% yields with 1:1–>20:1 diastereoselectivities and 84–99% ee.

### 5.3. AUyl Rhodium Species

**Scheme 51.**
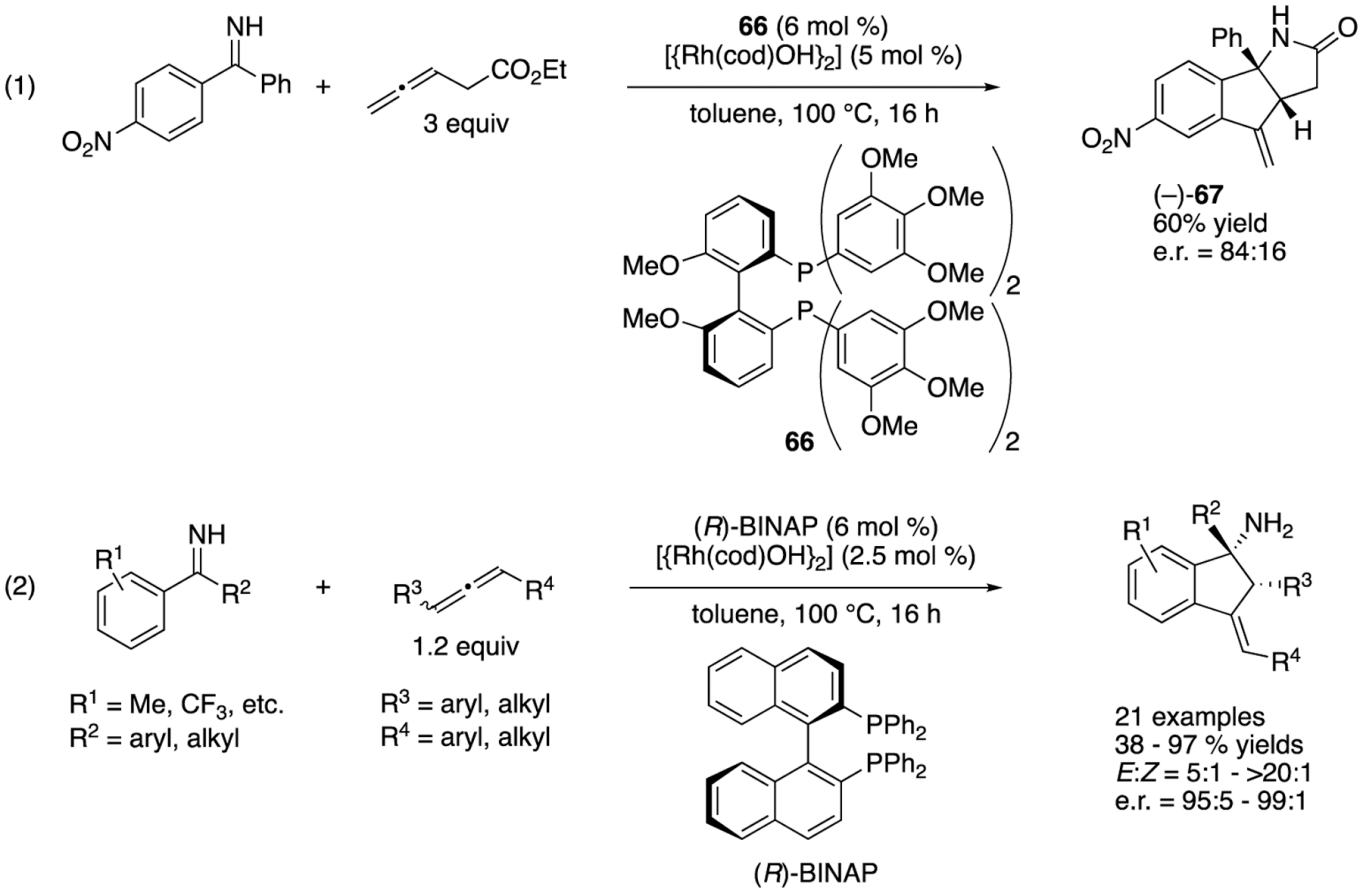
Rhodium-catalyzed asymmetric intramolecular allylation of ketimines.

In 2010, Cramer and a coworker reported one example of enantioselective rhodium(I)-catalyzed intramolecular allylation of a ketimine (Scheme 51, (1)) [138], This transformation was initiated by an imine-directed orthorhodation, followed by a carbometallation of the terminal bond of an allene that produced an allyl rhodium species, which in turn underwent an intramolecular ketimine allylation reaction to give the product where its ester group spontaneously cyclized on to the primary amine moiety formed. The absolute configuration of (−)-**67** was not determined. In 2013, they extended this protocol to Rh-catalyzed dynamic kinetic resolution of racemic allenes (2) [139], The substrate scopes of both ketimines and allenes were very broad, affording various cyclic α-tertiary homoallylamines in 38–97% yields with 5:1–>20:1 *E:Z* selectivities and 95:5–99:1 enantiomeric ratios. This protocol also used unprotected N–H ketimines that obviated a potentially problematic deprotection of the amine group in the products.

### 5.4. Allyltrichlorosilane

**Scheme 52.**
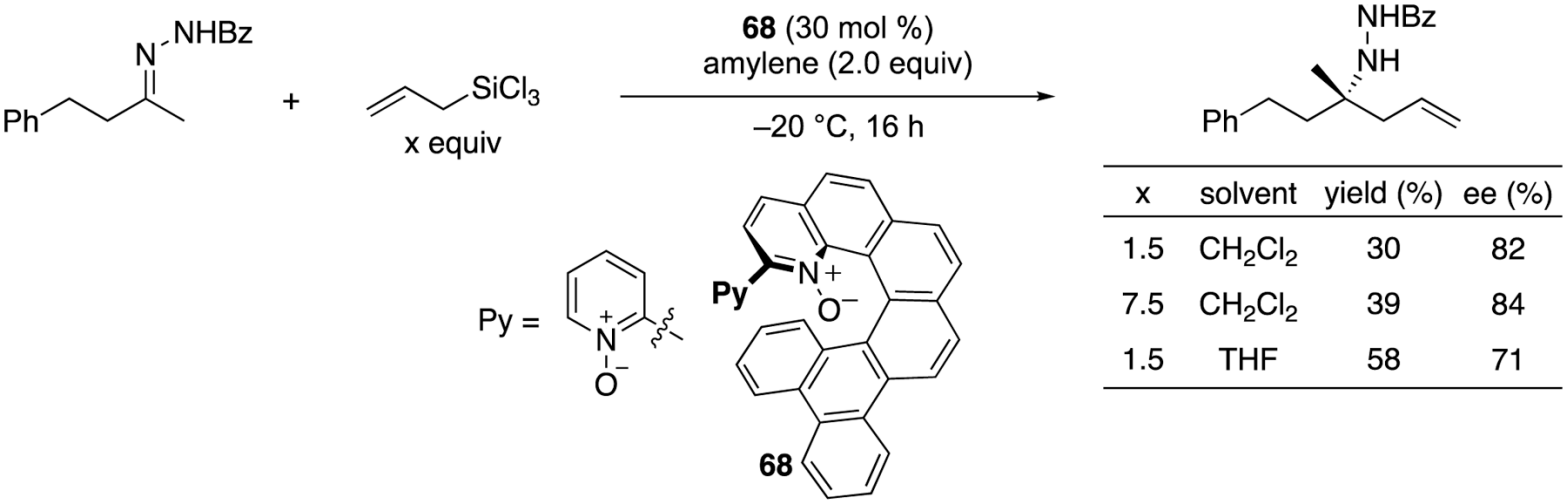
Chiral Lewis base-catalyzed allylation of an aliphatic ketimine.

In 2014, Peng and Takenaka described one example of enantioselective allylation of an aliphatic ketimine with allyltrichlorosilane catalyzed by their helical-chiral Lewis base catalyst (Scheme 52) [140]. Commercially available allyltrichlorosilane is an inexpensive easy-to-use allylation reagent. It is often employed for the allylation of aldehydes and aldimines, but its catalytic enantioselective addition to ketimines is not known to the best of our knowledge. Based on their preliminary mechanistic study, they hypothesized that a product (bearing a NSiCl_3_ unit before aqueous work-up) inhibited the catalyst from turning over efficiently in CH_2_Cl_2_. A more Lewis basic solvent, THF did improve the catalyst’s turnover but adversely affected the enantioselectivity.

## 6. Activated and/or Cyclic Ketimines in Aza-Morita-Baylis-Hillman Reaction

The asymmetric aza-Morita-Baylis-Hillman (aza-MBH) is widely recognized as a useful and atom-economical carbon-carbon bond formation reaction between electron-deficient alkenes and imines catalyzed by chiral Lewis bases (selected reviews; [141–144]). This transformation provides highly functionalized β-amino carbonyl compounds in an enantio-enriched form. As such, a number of important developments have been reported. However, the ketimine variant was not known until 2013, in which three independent studies were disclosed [145–147]. One of them employed isatin-derived ketimines [146], and more contributions with isatin-derived ketimines were disclosed later (selected references; [148–154]). In light of the excellent review articles on the isatin-derived ketimines that covered up to the end of 2017 [73–75], we discuss a few selected examples in Section 6.3 (vide infra).

### 6.1. Acyclic Ketimines Bearing a Carbonyl Moiety

**Scheme 53.**
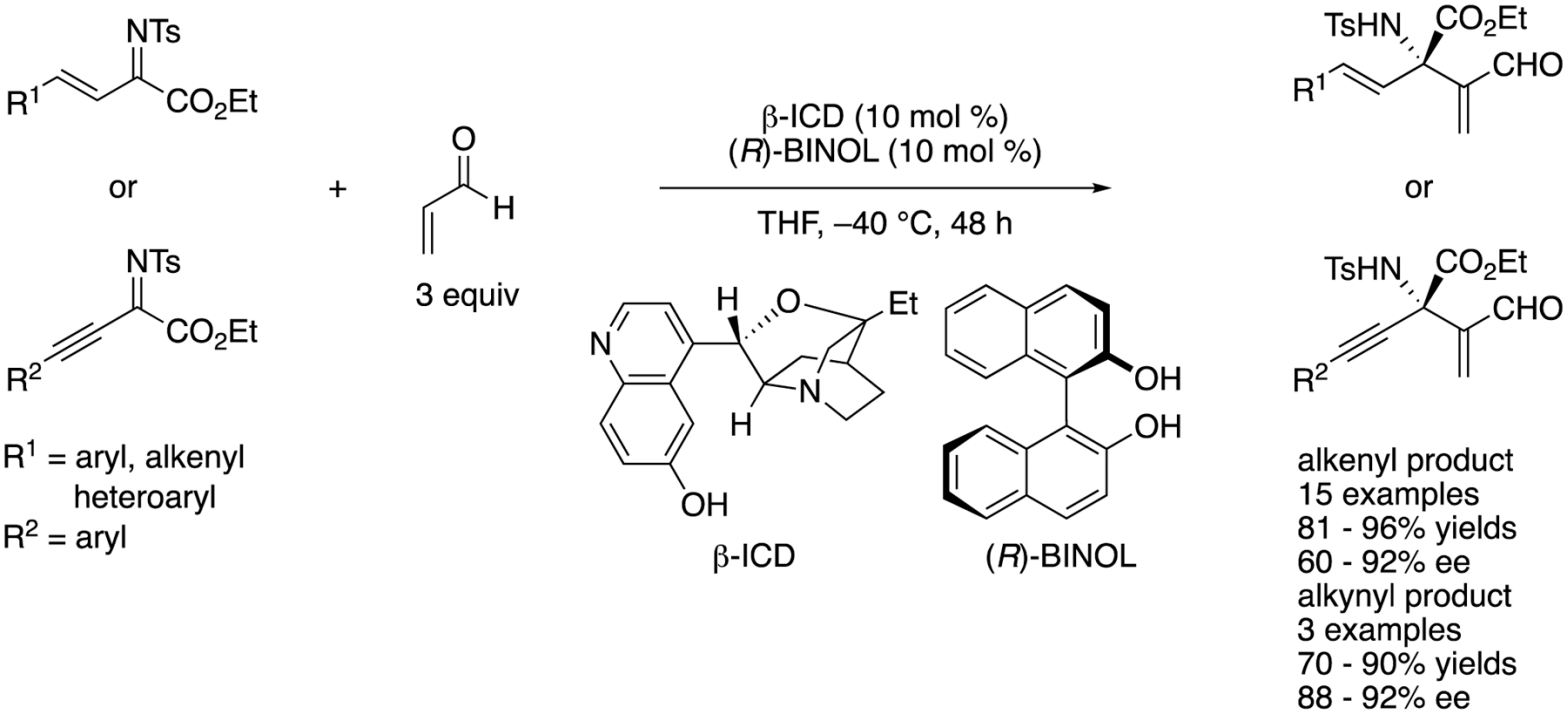
The first aza-Morita-Baylis-Hillman reaction of acrolein with ketiminoesters.

In 2013, Chen and coworkers developed an aza-MBH reaction of alkenyl or alkynyl ketiminoesters with acrolein catalyzed by β-isocupreidine ((β-ICD) (Scheme 53) [145]. During their optimization study, they found that some double-hydrogen-bond donors such as catechol, (*R*)-, (*S*)-BENOL, and a chiral thiourea enhanced the enantioselectivity of the model substrate (R^1^ = 4-Cl-Ph) from 65% ee to 87, 90, 90, and 90% ee, respectively, while yields were comparable (89, 92, 91, 89, and 91%, respectively). On the basis of the preliminary ^1^H NMR study of (β-ICD and phenolic additives, they proposed that a BINOL bridged a ketiminoester and a β-ICD-acrolein adduct through hydrogen bonding, leading to a more organized transition state. The optimized method worked for both alkenyl- and alkynyl-substituted ketiminoesters and provided the corresponding products in 81–96% yields with 60–92% ee and 70–90% yields with 88–92% ee, respectively.

**Scheme 54.**
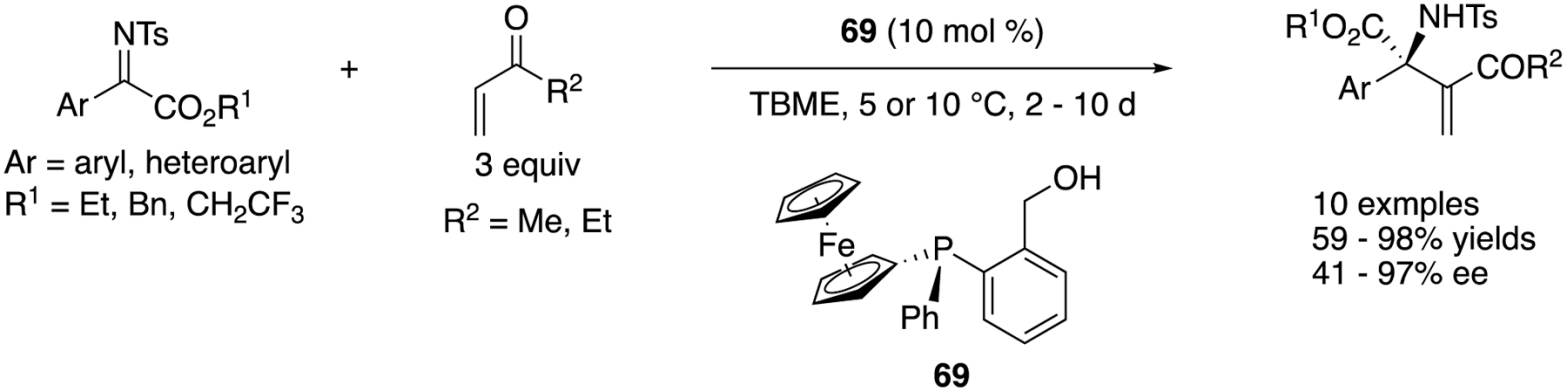
The P-chirogenic Lewis base-catalyzed aza-MBH reaction of alkyl vinyl ketones with ketiminoesters.

In the same year, Jugé and Sasai reported an aza-MBH reaction of methyl or ethyl vinyl ketones with ketiminoesters (Scheme 54) [147]. During their catalyst screening study, they identified that P-chirogenic Lewis bases were superior to other catalysts that were commonly employed for MBH reactions. After optimization of P-chirogenic catalyst structures and other reaction parameters, catalyst **67** provided the MBH products in 59–98% yields with 41–97% ee.

**Scheme 55.**
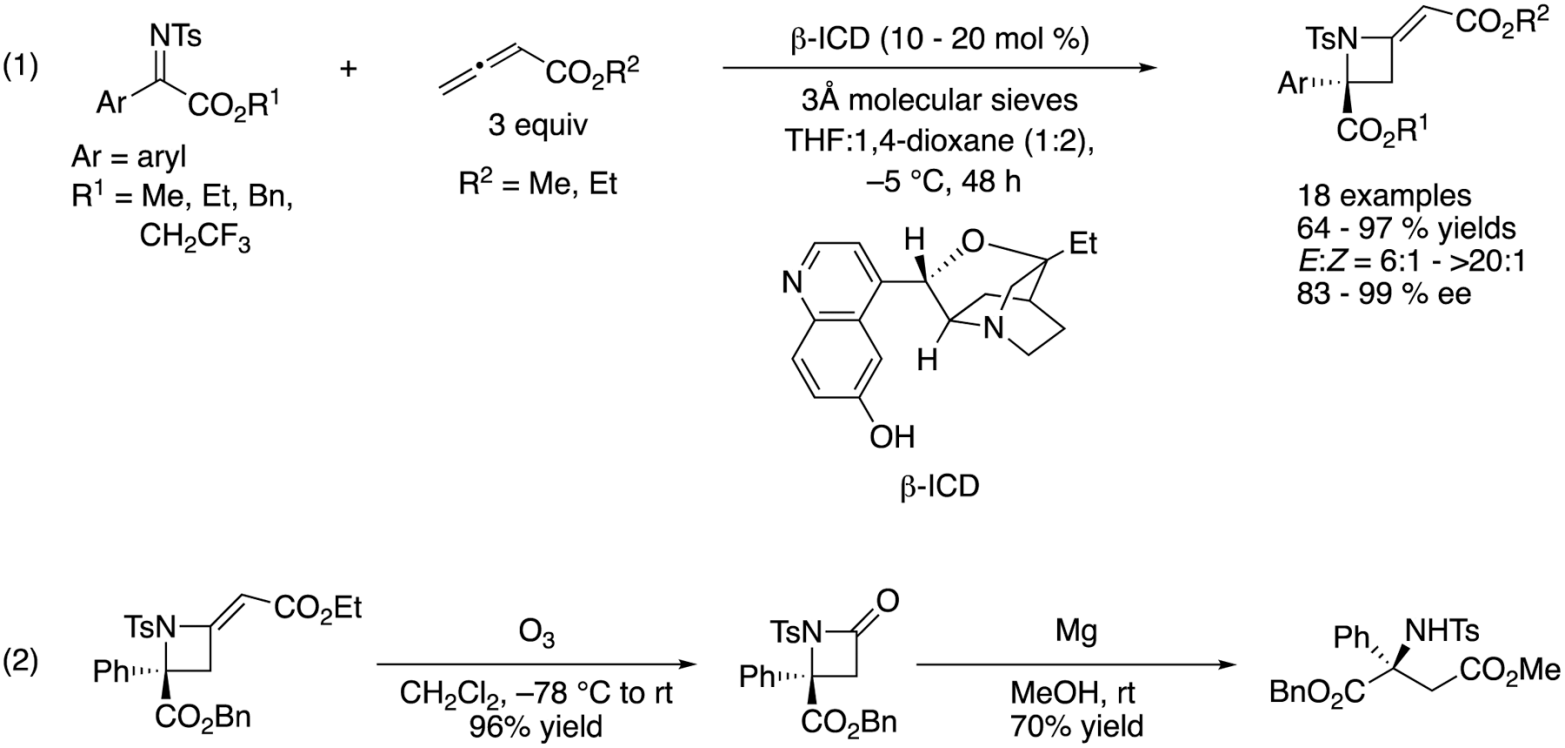
Chiral Lewis base-catalyzed formal [2 + 2] cycloaddition of ketiminoesters with allenoates.

In the same year, Sasai described a chiral Lewis base catalyzed formal [2 + 2] cycloaddition of ketiminoesters with allenoates (Scheme 55) [155]. This work represents the first example of catalytic enantioselective allenoate addition to ketimines (1). They screened various chiral amines and identified β-ICD as an optimal catalyst that provided the azetidines in 64–97% yields with 6:1–>20:1 E/Z selectivities and 83–99% ee. To demonstrate the synthetic utility of the products, they successfully converted an enantio-enriched azetidine to a β-amino carbonyl compound in two steps without any loss in its optical purity (2).

### 6.2. Endocyclic Ketimines

**Scheme 56.**
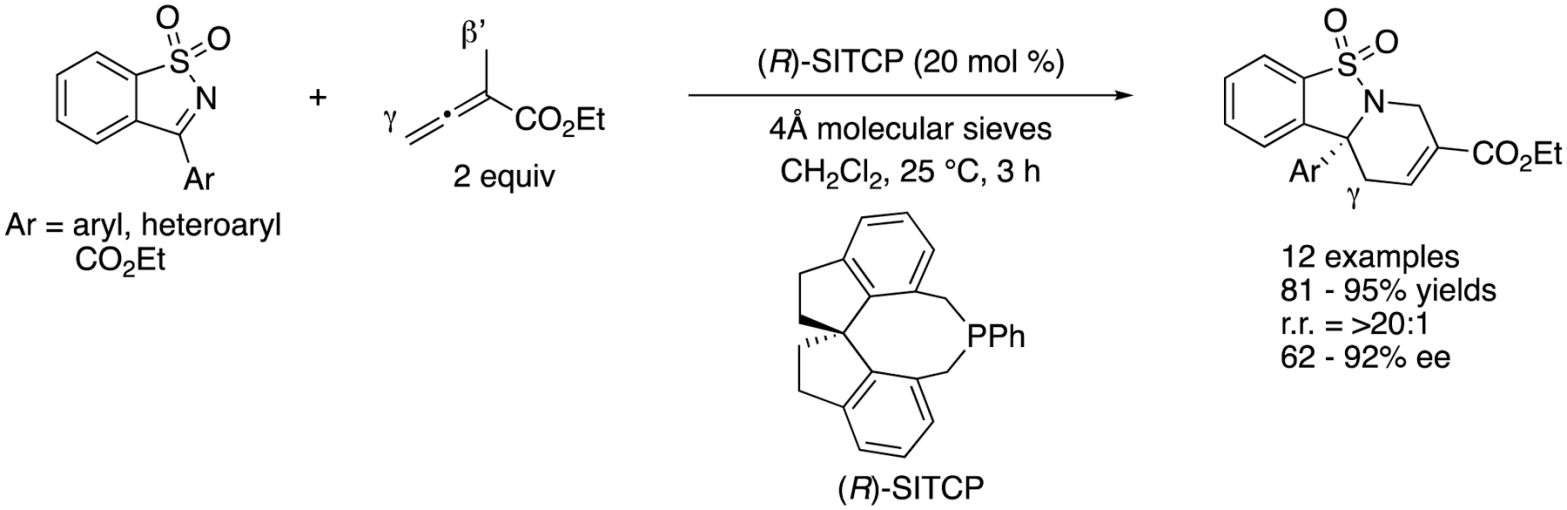
Enantioselective formal [4 + 2] cycloaddition of ketimines with α-methyl allenoate.

In 2014, Sasai and coworkers reported enantioselective Lewis base-catalyzed formal [4 + 2] cycloaddition of endocyclic *N*-sulfonyl ketimines with α-methyl allenoate (Scheme 56) [156]. After screening commonly used chiral phosphines, they found that (*R*)-SITCP efficiently promoted the desired transformation. This method readily provided enantio-enriched tetrahydropyridines bearing a chiral tetrasubstituted carbon stereogenic center in 81–95% yields with >20:1 regioselectivity and 62–92% ee. With respect to the observed regio-selection, they proposed that SITCP (monoaryl spiro-type phosphine catalyst) was relatively electron-rich, thus leading to the kinetically favored γ-addition to the ketimine through the zwitterionic allenoate-catalyst adduct, followed by intramolecular cyclization.

**Scheme 57.**
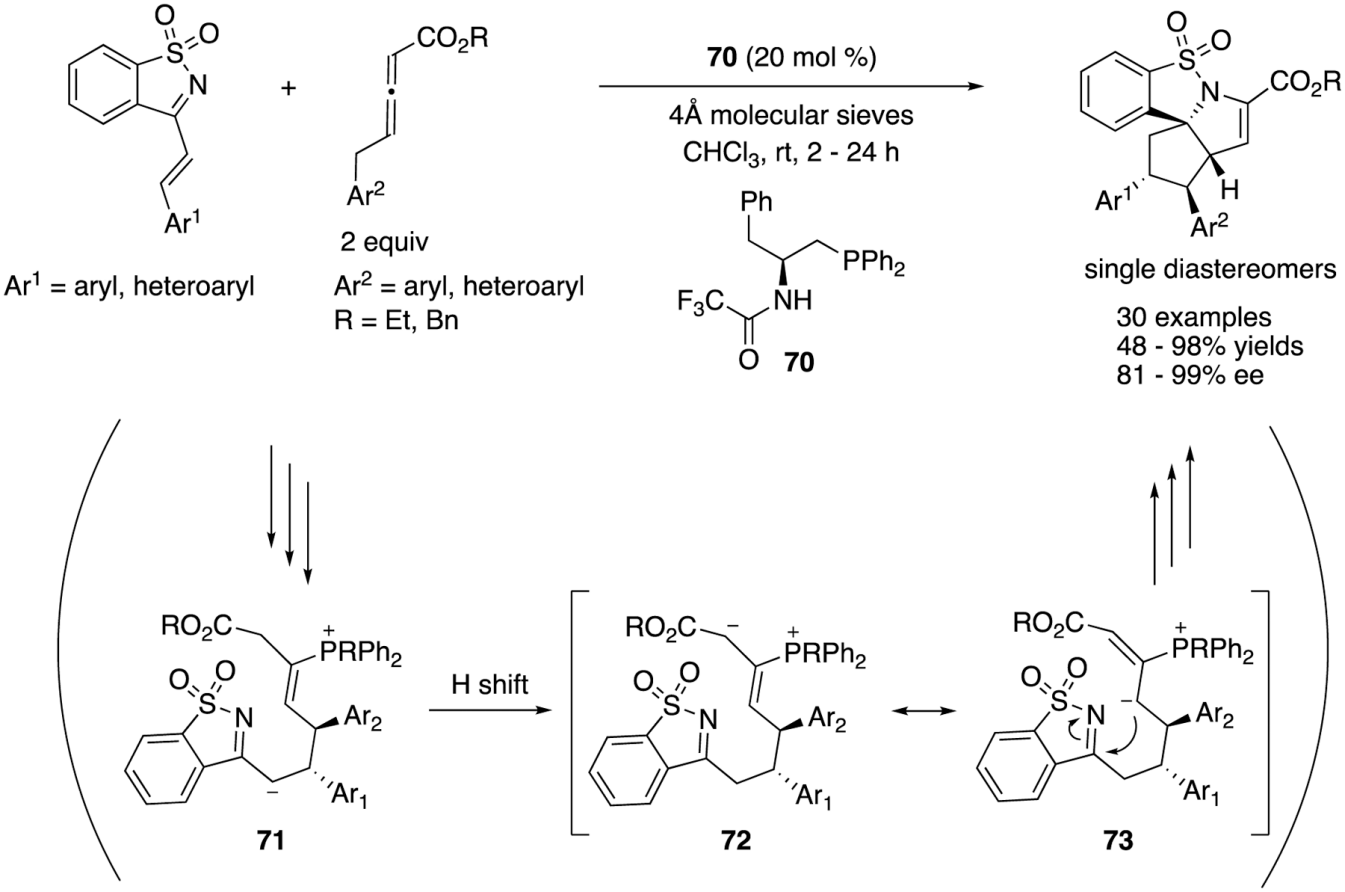
Chiral phosphine-catalyzed sequential annulation of allenoates and endocyclic ketimines.

In 2016, Huang and coworkers described a bifunctional-phosphine-catalyzed sequential annulation of endocyclic *N*-sulfonyl ketimines with γ-benzyl allenoates (Scheme 57) [157]. This transformation readily provided rapid access to polyheterocyclic products with four contiguous stereogenic centers (one quaternary and three tertiary carbon centers). It is noteworthy that only one isomer was obtained in all cases. As to the reaction pathway, it was proposed that a zwitterionic allenoate-catalyst adduct conjugatively added to an α,β-unsaturated ketimine to generate intermediate **71**, followed by a proton shift and intramolecular addition to the ketimine unit to form the first ring. The method tolerated various aromatic and heteroaroma tic substitutions for both substrates and provided the highly complex products in 51–98% yields with 81–99% ee.

### 6.3. Isatin-Derived Ketimines

**Scheme 58.**
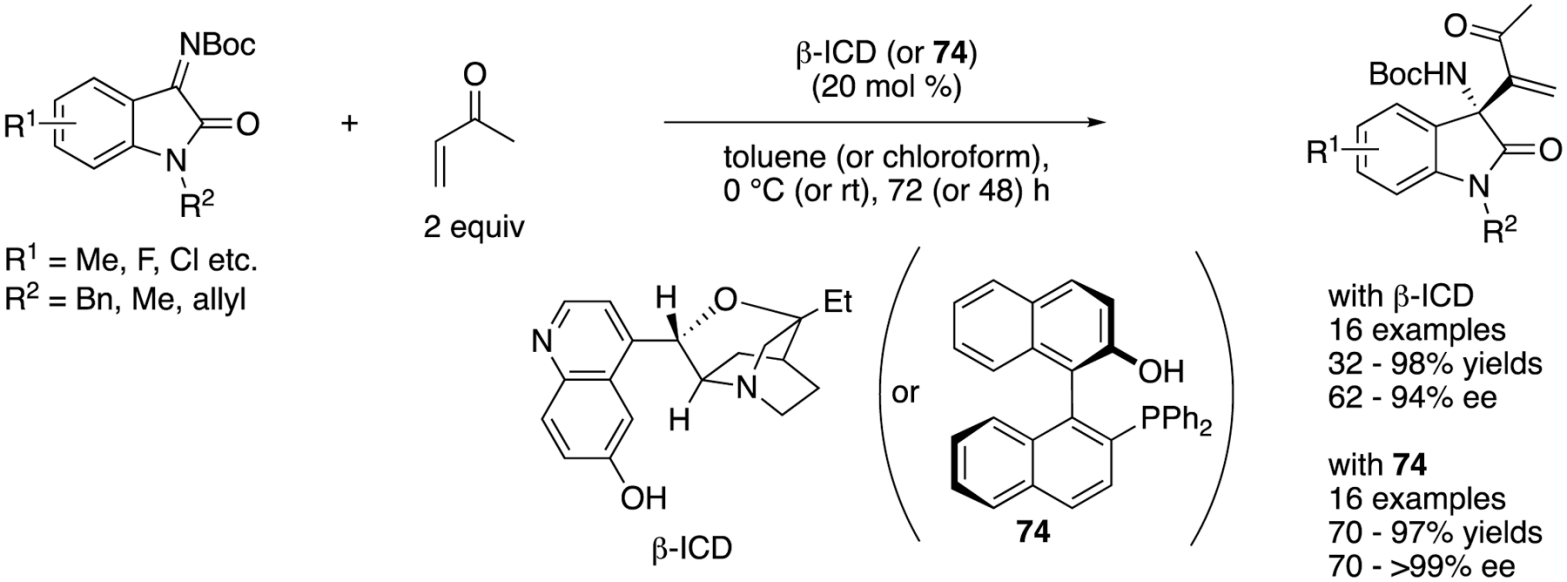
The first asymmetric catalytic aza-MBH reaction of isatin-derived ketimines.

In 2013, Shi, Li and coworkers described the first catalytic asymmetric aza-MBH reaction of isatin-derived ketimines with methyl vinyl ketone (Scheme 58) [146]. They evaluated both amine- and phosphine-based catalysts ((β-ICD and **74**, respectively) and optimized other reaction parameters for each catalyst. (β-ICD provided the products in 32–98% yields with 62–94% ee, and **74** afforded them in 70–97% yields with 70–>99% ee.

**Scheme 59.**
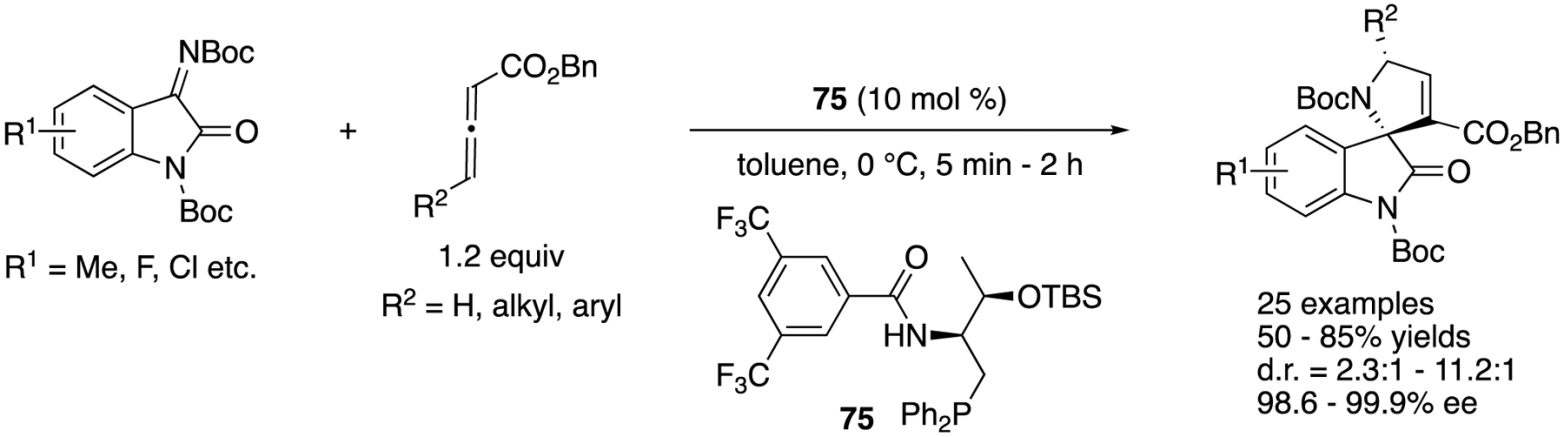
A chiral phosphine-catalyzed [3+2] cycloaddition reaction of allenoates and isatin-derived ketimines.

In 2016, Han, Lu, and coworkers reported an enantioselective phosphine-catalyzed [3+2] annulation reaction of allenoates and isatin-derived ketimines for the first time (Scheme 59) [148]. The method tolerated allenes with both alkyl and aryl substituents at the γ-positions and afforded various 3,2′-pyrrolidinyl spirooxindoles in 50–85% yields with 2.3:1–11.2:1 diastereoselectivities and 98.6–99.9% ee.

**Scheme 60.**
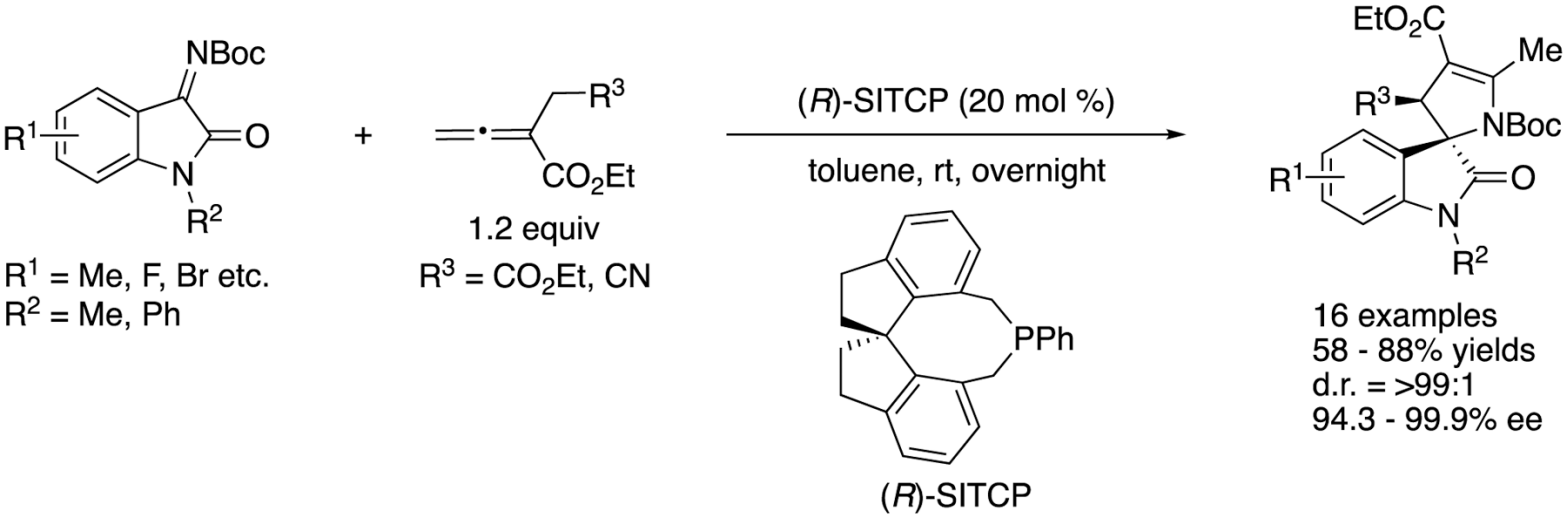
A chiral phosphine-catalyzed [3+2] annulation reaction of α-substituted allenoates and isatin-derived ketimines.

In 2016, Kumar and coworkers disclosed the first diastereo- and enantio-selective [3+2] annulation reaction of α-substituted allenoates with isatin-derived ketimines catalyzed by a chiral phosphine (Scheme 60) [149]. They screened various chiral phosphine-based catalysts and found that SITCP provided a desired 3,2′-pyrrolidinyl spiro-oxindole with high selectivities. Under the optimized reaction conditions, the catalyst gave the products in 58–88% yields with >99:1 diastereoselectivity and 94.3–99.9% ee.

## 7. Summary and Outlook

Because of the notorious structural properties of simple ketimines, chemists in general are under the impression that even ketimines with electron-withdrawing substituents are hard to utilize for catalytic asymmetric reactions. Driven by the clear benefits of Mannich reactions, various ketimines have been studied, and many notable achievements are reported in the literature. As such, we hope that these important contributions show that chemists now can readily build α-tertiary amine stereogenic centers into molecules of their interest by choosing activated ketimines adequate for their needs. On the other hand, the Mannich and its related reactions of unmodified ketimines currently remain as some of the most important persisting problems in asymmetric catalysis and synthesis, providing intellectually stimulating research opportunities.
